# Taxonomic profiling of individual nematodes isolated from copse soils using deep amplicon sequencing of four distinct regions of the 18S ribosomal RNA gene

**DOI:** 10.1371/journal.pone.0240336

**Published:** 2020-10-07

**Authors:** Harutaro Kenmotsu, Kiichi Uchida, Yuu Hirose, Toshihiko Eki

**Affiliations:** Department of Applied Chemistry and Life Science, Molecular Genetics Laboratory, Toyohashi University of Technology, Toyohashi, Aichi, Japan; Universitat Konstanz, GERMANY

## Abstract

Nematodes are representative soil metazoans with diverged species that play crucial roles in nutrient recycling in the pedosphere. Qualitative and quantitative information on nematode communities is useful for assessing soil quality, and DNA barcode-mediated taxonomic analysis is a powerful tool to investigate taxonomic compositions and changes in nematode communities. Here, we investigated four regions (regions 1–4) of the 18S small subunit ribosomal RNA (SSU) gene as PCR targets of deep amplicon sequencing for the taxonomic profiling of individual soil nematodes. We determined the sequence variants (SVs) of 4 SSU regions for 96 nematodes (total 384 amplicons) isolated from copse soils and assigned their taxonomy using the QIIME2 software with dada2 or deblur algorithm and the SILVA database. Dada2 detected approximately 2-fold more nematode-derived SVs than deblur, and a larger number of SVs were obtained in regions 1 and 4 than those in other regions. These results and sufficient reference sequence coverage in region 4 indicated that DNA barcoding using a primer set for region 4 followed by dada2-based analysis would be most suitable for soil nematode taxonomic analysis. Eighteen SSU-derived operational taxonomic units (rOTUs) were obtained from 68 isolates, and their orders were determined based on the phylogenetic trees built by four regional sequences of rOTUs and 116 nematode reference species as well as the BLASTN search. The majority of the isolates were derived from three major orders Dorylaimida (6 rOTUs, 51.5% in 68 isolates), Rhabditida (4 rOTUs, 29.4%), and Triplonchida (7 rOTUs, 17.6%). The predicted feeding types of the isolates were fungivores (38.2% in total nematodes), plant feeders (32.4%), and 14.7% for both bacterivores and omnivores/predators. Additionally, we attempted to improve the branch structure of phylogenetic trees by using long nucleotide sequences artificially prepared by connecting regional sequences, but the effect was limited.

## Introduction

A recent worldwide survey for nematodes has confirmed that nematodes are one of the most abundant metazoans in the pedosphere [1, 2]. Nematodes are widely distributed in freshwater, terrestrial, and marine environments on Earth and exhibit a variety of different feeding habits such as bacterial and fungal feeding, predation, and animal or plant parasitism [3]. Nematodes play crucial roles in the nutrient cycling of soil biota and influence plant growth [4, 5]. Nematode taxonomic compositions vary by ecosystem type [6] and are influenced by various factors such as food, temperature, humidity, pH and nutrients in soils [7], soil properties [7, 8], cultivated plants [9], tillage [10], and fertilizers [11, 12]. These characteristics strongly indicate that the taxonomic composition of nematodes can be used as an excellent indicator for assessing biological, chemical, and nutrient status in soils [13, 14]. Many previous studies have conducted morphology-based taxonomic classification on soil nematodes; however, this method is low-throughput and can produce vague results. Morphology-based classification also requires careful observation under the microscope by highly skilled and experienced experts since the anatomical structure of most nematodes is very similar. These challenges have required and accelerated the development of efficient alternative methods for analyzing soil nematode communities [15].

In order to efficiently utilize nematode community data for soil assessment, accurate, conventional, and high-throughput methods for the taxonomic analysis of soil nematodes are required. One of the most suitable methods is DNA barcode-based taxonomic analysis, which is an accurate and high-throughput sequence-based assignment of a tested organism to taxon and has been applied in the taxonomic studies of various organisms [16, 17]. This approach can be used to identify not only the taxonomic rank of an individual organism but also the taxonomic compositions of an organism’s community [17]. Recently, metagenomic approaches have been extensively used for the quantitative analysis of the microbiome of various environments (e.g., human guts) using next-generation sequencing (NGS). In the DNA barcoding of nematodes, the nucleotide sequences of DNA barcode from individual nematodes were determined by PCR amplification followed by DNA sequencing, and then the nematodes were classified by their sequences to taxonomic groups sharing the identical DNA barcode sequences, which are called operational taxonomic units (OTUs) [18]. The number of OTUs and abundance of nematodes in each OTU provides us with important quantitative and qualitative information on the nematode community: the former indicates the number of taxonomic groups (i.e., nematode species diversity), and the latter shows the proportion of each taxonomic group in the nematode community.

We previously analyzed nucleotide sequences of the 18S small subunit ribosomal RNA (SSU) and mitochondrial cytochrome oxidase subunit I (COI) genes as DNA barcodes from individual nematodes isolated from two different environmental soils (i.e., an agricultural field and an unmanaged flowerbed) by Sanger sequencing [19]. These analyses successfully confirmed that the nematode community in each soil had distinct feeding habits: bacterial feeding in the field soil, and animal predation and plant feeding in the flowerbed soil, respectively. Thus, DNA sequence-based taxonomic analysis is a powerful tool for studying soil nematodes in various ecosystems. In the past decade, the extensive advancement of DNA sequencing technology has transformed from the “one-by-one DNA barcoding” method using Sanger sequencing of a PCR amplicon from an individual organism, to the “DNA metabarcoding” method using NGS-assisted massive sequencing of an amplicon from a complex community. Currently, several studies concerning the DNA metabarcoding of terrestrial [20–25] and marine nematodes [26–28] have been conducted using deep sequencing of SSU-derived amplicons. However, potential regions of the SSU gene that would be most suitable for NGS-based DNA metabarcoding have not been investigated in detail yet.

Here, we performed NGS-assisted DNA barcoding of individual soil nematodes using primer sets that amplify four regions of the 18S rRNA gene to find optimal regions for the Illumina MiSeq-assisted amplicon sequencing. In the experiment, we analyzed the nucleotide sequences of 384 amplicons from 4 SSU regions of 96 individual soil nematodes isolated from copse soils in Japan. We detected sequence variants (SVs), including polymorphic alleles in each region, using two computer algorithms dada2 and deblur. The 18S ribosomal small subunit RNA gene-derived OTUs (rOTUs) were further identified by clustering isolates using the resultant nematode-derived SVs. We further investigated their taxonomic ranks using SILVA-based classification, the BLASTN search, and phylogenetic trees with 116 nematode reference species. Additionally, we have examined the effects of artificially combined long sequences in the preparation of these phylogenetic trees by comparing them with trees built by regional short sequences to examine whether or not the use of artificial long sequences could improve the structure of phylogenetic trees.

## Materials and methods

### Soil sampling

**Fig 1:**
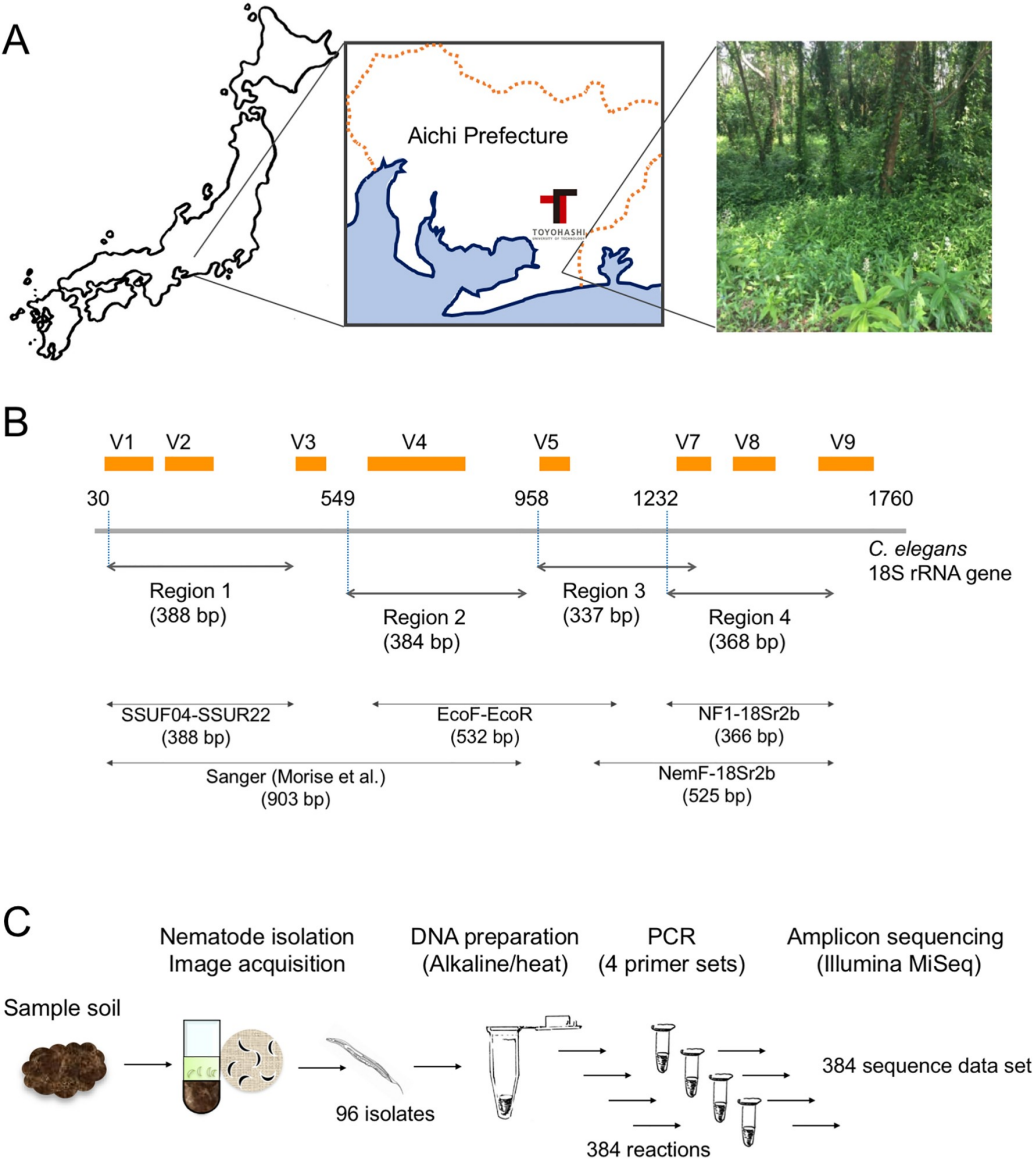
Soil sampling site, PCR target regions of 18S rRNA gene, and experimental scheme of this study. Sample soils for nematode isolation were obtained from the copse (picture) at the campus of Toyohashi University of Technology, Aichi Prefecture, Japan (A). Four PCR target regions (regions 1–4, with amplicon sizes shown) used in this study are indicated by double-headed arrows (B). The numbers indicating the nucleotide positions of the 5’-end of forward primers are shown on the entire SSU gene prepared from the nucleotide sequence of the *C*. *elegans* ribosomal RNA gene cluster (X03680). Orange boxes correspond to the hypervariable regions of the eukaryotic SSU genes reported by Hugerth et al. [38] and Hadziavdic et al. [45]. The regions that were amplified by the four indicated published primer sets (SSUF04-SSUR22 [29], EcoF-EcoR [24], NF1-18Sr2b [20], and NemF-18Sr2b [21]), as well as the amplified region from our previous study [19], are indicated by double-headed arrows with amplicon sizes shown. The experimental scheme of the amplicon sequencing that was prepared from the four SSU regions using 96 nematode isolates is shown (C). Fig 1 was produced by the authors specifically for this manuscript.

Soil samples were collected between July and August 2018 under clear climatic conditions (temperature: 28 °C–35 °C; humidity: 64%–82%) from a copse at the campus of Toyohashi University of Technology, Toyohashi, Japan (137°24’E, 34°42’N [longitude: 137.4086, latitude: 34.7017]) located in the temperate zone (Fig 1A). The soil at the site was sampled to a depth of 15–20 cm using a 2.5 cm-diameter soil sampling auger (Fujiwara Scientific Co., Tokyo, Japan), and more than three independent soil samples were taken and mixed from each site. Following the removal of over-sized contaminants (e.g., stones) by filtering soil samples through a 0.7 mm sieve, approximately 10 g of fresh weight soil was immediately used for nematode isolation within a day of collection.

### Nematode isolation and DNA preparation

The isolation of nematodes from fresh soil samples was performed using the improved flotation sieving method with colloidal silica, as previously described [19]. Nematodes trapped on both sieves were eluted into water in a watch glass, and digital images of the individual nematodes were acquired using an IX71 inverted microscope (Olympus, Tokyo, Japan) equipped with a PD71 CCD camera (Olympus). Each nematode was picked up using a micropipette (P-20, Gilson, Middleton, WI, USA) with a cut tip while being observed under an SZX16 stereomicroscope (Olympus), and they were then transferred into a DNA LoBind tube (Eppendorf, Hamburg, Germany). The nematode in the tube was heated at 99 °C for 3 min in 20 μL of 0.25 M NaOH and then neutralized by adding 4 μL of 1 M HCl, 10 μL of 0.5 M Tris-HCl (pH 8.0), and 5 μL of 2% Triton X-100. DNA samples were stored at −20 °C until use.

### PCR and DNA sequencing

Four sets of PCR primers with tail sequences for Illumina MiSeq sequencing were obtained from Fasmac (Atsugi, Japan) (Table 1) and used for amplification of the 18S small subunit ribosomal RNA (SSU) gene fragments of the corresponding regions 1 to 4 (Fig 1B). The PCR reaction mixture (20 μL) contained 10 μL of 2 × PCR buffer for KOD FX Neo, 4 μL of 2 mM dNTPs, 0.4 unit of KOD FX Neo DNA polymerase (Toyobo, Tokyo, Japan), 2 μL of individual nematode DNA, and 0.3 μM each of the forward and reverse primers. The PCR product was purified with 0.8 volumes of AMPure XP beads (Beckman-Coulter, Indianapolis, IN, USA), according to the manufacturer’s instructions, and eluted with 10 mM Tris-HCl (pH 8.5). Index PCR was performed in a thermocycler for eight cycles using a Nextera XT Index Kit v2 (Illumina, San Diego, CA, USA), according to the manufacturer’s instructions. The independent index was used for the 18S rRNA amplicon that was obtained from each reaction. The amplified libraries were purified by the addition of 1.12 volumes of AMPure XP beads, according to the manufacturer’s instructions, and eluted with 10 mM Tris-HCl (pH 8.5). The concentration of each library was quantified using a spectrophotometer, and equal amounts of the libraries were pooled and quantified using a Qubit dsDNA HS Assay Kit (Thermo Fisher Scientific, Waltham, MA, USA). Each 300 base pair (bp) end of the pooled library was sequenced using a MiSeq Reagent Kit v3 (600 cycles; Illumina) on a MiSeq instrument (Illumina). The sequences were deposited in the DDBJ Sequence Read Archive database under the accession number DRA010544 with BioProject ID PRJDB10241 and BioSample IDs SAMD00236598 to SAMD00236693.

**Table 1:** PCR primers used for amplification of four regions of 18S rRNA gene.

Target region	Primer	Nucleotide sequence (5’–3’)
Region 1	SSU18A-4F3_MiseqF	tcgtcggcagcgtcagatgtgtataagagacagGCTTRTCTCAAAGATTAAGCCATGCATG
	SSU_R22_MiseqR	gtctcgtgggctcggagatgtgtataagagacagGCCTGCTGCCTTCCTTGGA
Region 2	SSUconsF1_MiseqF	tcgtcggcagcgtcagatgtgtataagagacagAGCAGCCGCGGTAATTCCAGCTC
	SSU26Rplus4_MiseqR	gtctcgtgggctcggagatgtgtataagagacagAAGACATTCTTGGCAAATGCTTTCG
Region 3	Nem_18SR_ExtF_MiseqF	tcgtcggcagcgtcagatgtgtataagagacagGTTCGAAGGCGATYAGATACCGCC
	SSU_R23plus7_MiseqR	gtctcgtgggctcggagatgtgtataagagacagTCGYTCGTTATCGGAATWAACCAGAC
Region 4	NF1_MiseqF	tcgtcggcagcgtcagatgtgtataagagacagGGTGGTGCATGGCCGTTCTTAGTT
	18Sr2b_ExtR_MiseqR	gtctcgtgggctcggagatgtgtataagagacagGGTGTGTACAAAKSGCAGGGACGTA

Nucleotide sequences in lower-case letters indicate the tail sequence required for Illumina MiSeq sequencing. The primers for regions 1–3 were modified from the primers by Blaxter et al. [29] for PCR using tailed primer sets. The primer set for region 4 was derived from the previous primers reported by Porazinska et al. [20] with some modifications.

### DNA sequence data analysis

The sequence reads were imported into QIIME2 version 2019.10 (https://qiime2.org) [30]. The SVs were obtained using the dada2 [31] and deblur [32] packages. For sequence analysis using the deblur algorithm, the forward and reverse reads were joined, denoised, and chimera-checked using the deblur plugin with—p-trim-length options of 350 (region 1), 320 (region 2), 280 (region 3), and 310 (region 4), respectively. For sequence analysis using the dada2 algorithm, the forward and reverse reads were joined, denoised, and chimera-checked using the dada2 plugin with—p-trunc-len-f and—p-trunc-len-r options of 220, 220 (region 1), 225, 200 (region 2), 220, 220 (region 3), and 220, 220 (region 4), respectively. The default parameters in other options were used for each analysis. The primer sequences were removed by Cutadapt (version 2.4) [33]. The taxonomy of the SVs was assigned using a feature-classifier plugin that was trained with the taxonomy information in majority_taxonomy_all_levels.txt of 99% clustering in SILVA version 132 (https://www.arb-silva.de/download/archive/) [34]. Histograms of the four regional SVs with taxonomic information were drawn using the *R* phyloseq package [35]. In brief, taxonomic rank data for the SVs was obtained from the SILVA database, confirmed, and correctly assigned to phylum or nematode order by manual inspection. Then the frequency of the SVs in each sample was changed to relative abundance using phyloseq. For the preparation of the phylum level of the histograms, SVs derived from the same phylum were combined into a single phylum fraction in every sample, and then phylum fractions with less than 1% of relative abundance were removed. The relative abundance of each remaining phylum was indicated in the histogram of each sample by color. The order level of the histogram of nematode-derived SVs was prepared, as described below. The samples which had more than 1,000 reads and more than 65% of relative abundance of nematode-derived SVs (including over 7% of polymorphic nematode-derived SVs) were selected. Following the removal of non-nematode-derived SVs, the histogram of relative abundance of nematode-derived SVs were drawn together with their orders by color.

The nematode isolates sharing identical SVs in each region (regional SVs) were collected, and the resultant groups were defined as SSU (18S ribosomal RNA)-derived operational taxonomic units (rOTUs). The regional SVs corresponding to the rOTU in each region were re-named as regional rOTUs that were designated as “rOTU_number_R plus region number” such as rOTU01_R1 (i.e., the SV in region 1 that corresponds to rOTU01). Taxonomic analysis of the rOTUs was performed in three ways. First, the taxonomic ranks of the regional rOTUs were determined based on the SILVA database. Second, the taxonomic assignment of the regional rOTUs was investigated according to the closest species hit using the BLAST search of the National Center for Biotechnology Information (NCBI) website (https://www.ncbi.nlm.nih.gov) in April and July 2020. Third, phylogenetic trees of the reference nematode species with or without regional rOTUs were constructed to assign their nematode taxon, as described below. Briefly, the full-length nucleotide sequences of SSU genes and the taxonomic information of the nematode species were obtained from the NCBI website in April 2020. We attempted to select publicly available complete nucleotide sequences from one nematode species per family (Note: 8 of 57 families were limited in the suborder Spirurina) and successfully covered 17 orders in the phylum Nematoda, except for two orders: Muspiceida and Rhaptothyreida. Truncated or incomplete nucleotide sequences were removed; however, the nearly complete sequences of three species (*Ceramonema altogolfi*, *Proplatycoma* sp., and *Tarvaia* sp.) with partially truncated 5’-terminus were used in this study. These species were indicated as “Q_scientific name” in the phylogenetic trees. The 117 nucleotide sequences from the resultant 116 nematode species representing each family, and *Halobiotus crispae* (phylum Tardigrada) as the outgroup, were used for further phylogenetic analyses (S1 Table). The nucleotide sequences of each SSU region from the reference species were distinguished by assembling PCR primer sequences and the nucleotide sequence of the SSU gene using ATGC software (version 6, Genetyx Co., Tokyo, Japan). They were then cut out from the full-length sequence to prepare files of each of the regional sequences of the reference species using Genetyx-MAC software (version 19, Genetyx Co.). The nucleotide sequences were aligned, and phylogenetic trees were constructed using the BOOTSTRAP N-J TREE algorithm (bootstrap: 1000 replicates) with the ClustalX (version 2.1) package (http://www.clustal.org/clustal2/) [36]. The resultant tree files were used to draw the phylogenetic trees as cladograms using the Genetyx-Tree software (version 2.2.6, Genetyx Co.). The nucleotide sequences of the regional rOTUs were deposited in the DDBJ under the following accession numbers: LC570440–LC570747.

## Results

### Experimental design and nematode isolation from copse soils

**Fig 2:**
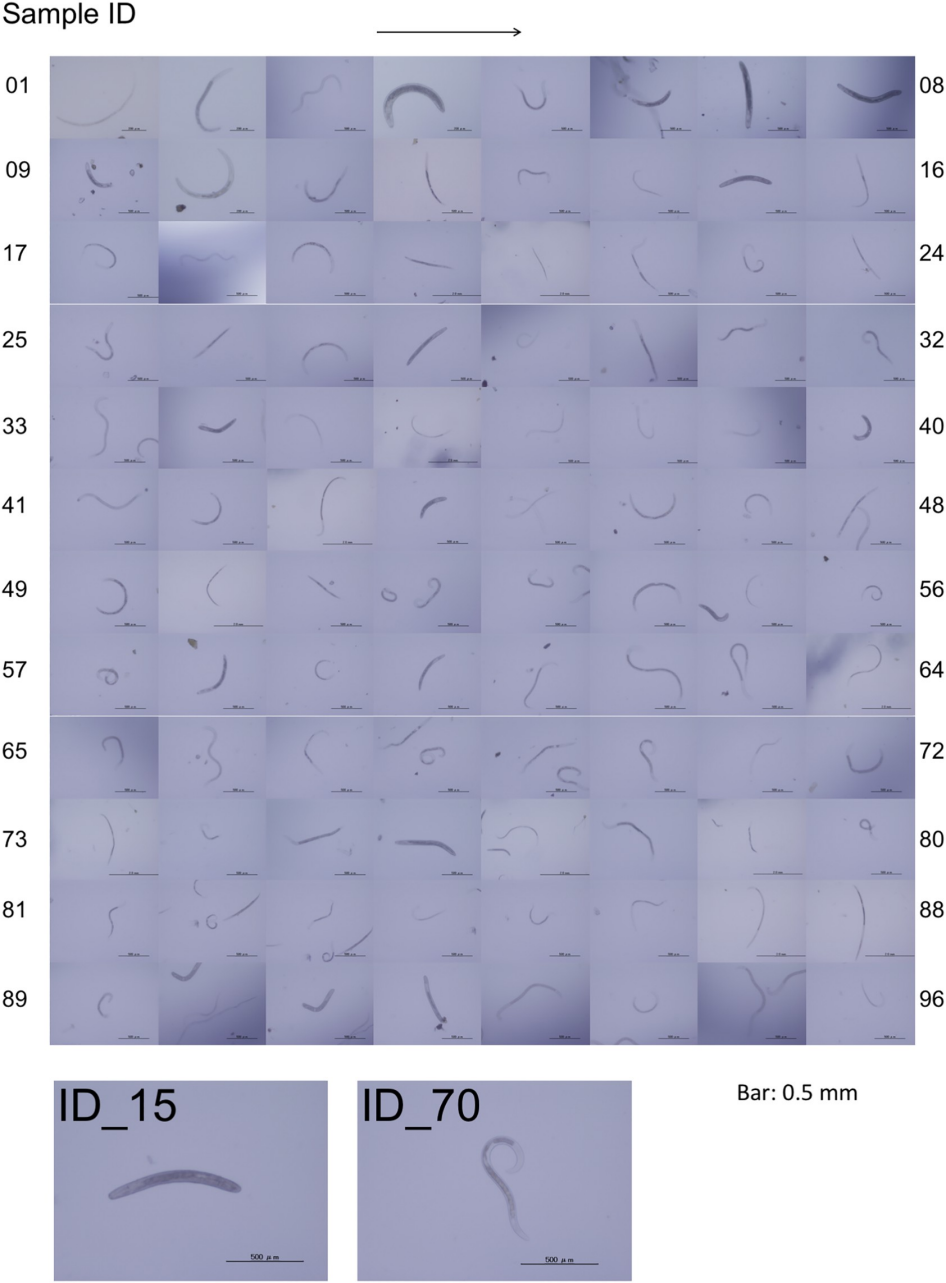
Images of 96 nematode isolates. Ninety-six images of nematodes are shown with their sample IDs. The sample ID numbers increase from left to right (as indicated by an arrow) and from top to bottom. Typical images for two morphologically distinct nematodes (sample ID_15 and ID_70) are shown below. Bars: 0.5 mm.

In this study, we performed DNA barcoding analysis using individual nematode isolates from copse soils using amplicon sequencing on the Illumina MiSeq platform to assess the most suitable regions of the SSU gene for DNA metabarcoding of soil nematodes (Fig 1C). Using the centrifugal flotation and sieving methods, we have randomly isolated 96 nematodes from the copse soils at the campus of Toyohashi University of Technology (Toyohashi, Aichi Prefecture in Japan) (Fig 1A) during July and August 2018. After acquiring their images, 96 sample DNAs were prepared from individual nematodes. Upon isolation of the nematodes, we attached the sample ID (ID_01–96) to each nematode and acquired their images (Fig 2). Although most nematodes had typical slender shapes (e.g., Fig 2, ID_70), 15 nematodes (ID_04, 06, 07, 08, 09, 15, 28, 34, 40, 44, 58, 60, 76, 91, and 92) were morphologically distinct with characteristic shapes of ring nematodes belonging to the family Criconematidae (Fig 2, ID_15) [37].

We investigated four PCR target regions of the SSU gene for assessing optimal regions for DNA barcoding, which were designated as region 1 to region 4 from the 5’-to -3’ direction of the gene (Fig 1B). Regions 1 and 4 were identical to those amplified by the primer sets of SSUF04 and SSUR22 [29], and NF1 and 18Sr2b [20], respectively. Region 1 covered both V1 and V2 hypervariable regions, and region 2 spanned the V4 regions of the eukaryotic 18S rDNA sequences reported by Hugerth et al. [38]. Both PCR target regions have been used in previous DNA barcode analyses of nematode communities using the next-generation sequencer Roche 454 [20, 21, 26, 39] or Illumina MiSeq [22, 23, 25, 27, 28, 40, 41]. Regions 2 and 3 were newly examined in this study and spanned the V4 region and the V5–V7 regions, respectively (Fig 1B). Regions 1 and 2 are separated by 131 nucleotides and regions 3 and 4 overlap on a 63 bp-region on the *C*. *elegans* SSU gene. The length of the amplicon generated from each region was adjusted to approximately 300–400 bp for the Illumina sequencing reaction. Four primer sets containing MiSeq-tail sequences were generated by referring to previous studies and adding sequence modifications for increasing PCR efficiency (Table 1).

### Amplicon sequencing and identification of SVs in four SSU regions

**Fig 3:**
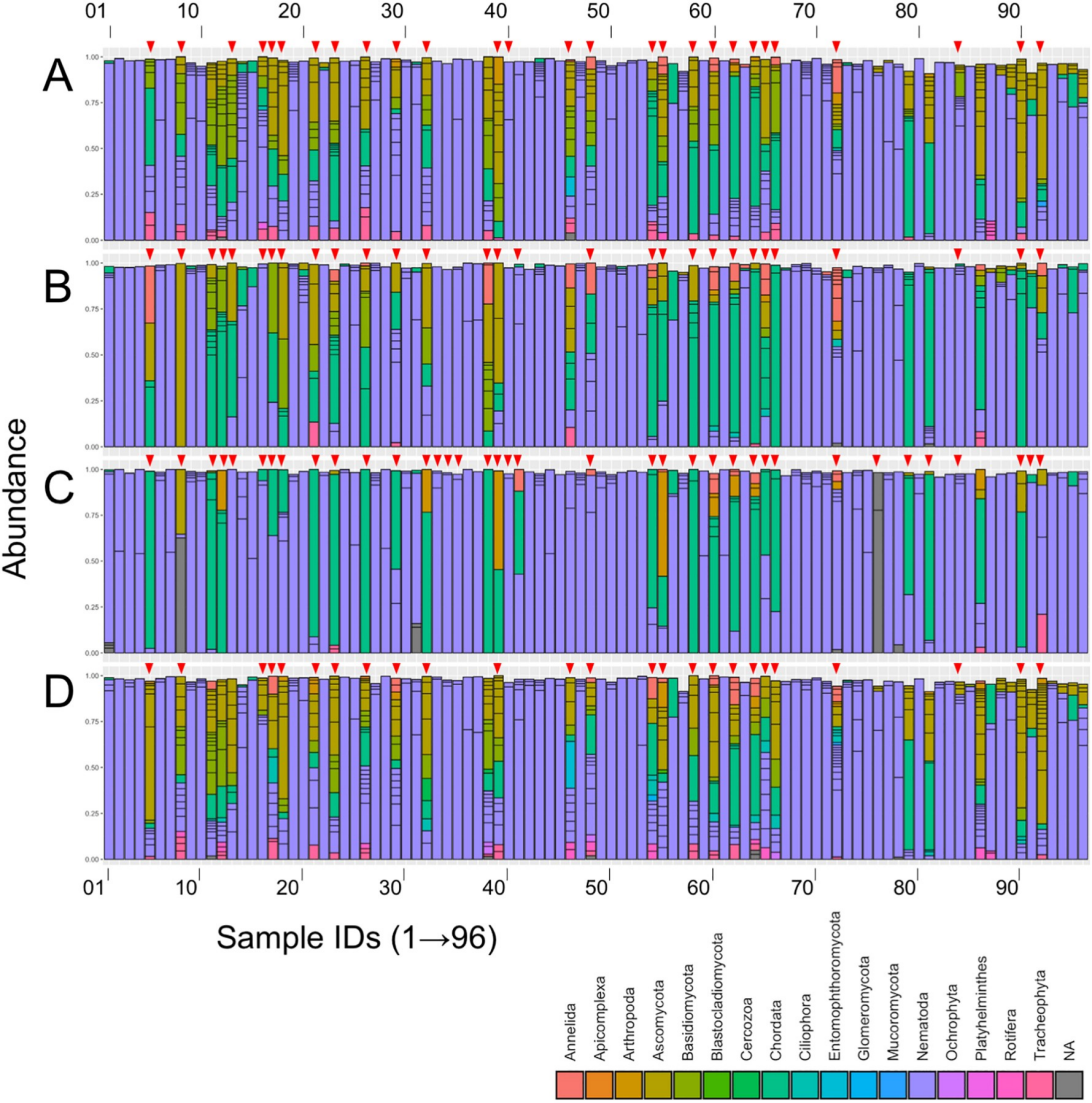
Histograms of sequence variants (SVs) at the phylum level in the samples. Relative abundance and phylum of SVs obtained from regions 1 (A), 2 (B), 3 (C), and 4 (D) in each sample are indicated in the histogram by color. The sample ID numbers are aligned from left to right, and the colors corresponding to phylum are shown in a legend box. Red arrow heads at the top of the histograms indicate sample IDs exhibiting poor PCR amplifications. NA: Not assigned.

Four PCRs in every 96 nematode DNA samples were performed, and the resultant 384 PCR products were independently tagged with sequences. Then, the nucleotide sequences of four DNA barcodes were determined using Illumina MiSeq. All PCR products were fractionated in a 1% agarose gel electrophoresis and visualized using successive ethidium bromide staining (S1 Fig). Based on visual inspection of the gel images, PCR products were poorly observed in approximately one-quarter of the reactions: 27, 29, 37, and 25 reactions for regions 1, 2, 3, and 4, and the corresponding PCR success rate was 71.8%, 70.0%, 61.5%, and 74.0%, respectively. Poor amplifications were commonly found in the 24 reactions indicated by red triangles on top of the corresponding histograms in all four regions (Fig 3). The nucleotide sequence data obtained by subsequent sequencing of 384 PCR products were processed as described in Materials and methods, and the SVs in each region were identified using two different algorithms, dada2 [31] and deblur [32]. To identify the nematode-derived SVs, the taxonomic ranks of the resultant SVs were successively determined using the SILVA database [34]. 203, 131, 97, and 185 nematode-derived SVs were identified by dada2 from 554, 352, 158, and 511 of the total SVs in regions 1–4 (Table 2). In contrast, approximately half of the number of nematode-derived and total SVs were identified by deblur: 108, 61, 43, and 96 nematode-derived SVs out of 286, 177, 86, and 281 of total SVs in the above regions. The number of nematode-derived SVs obtained from region 3 was less than half of those from regions 1 and 4. However, it should be noted that the proportion of nematode-derived SVs in the total SVs was 50% (deblur) and 61% (dada2), but approximately 34%–37% in other regions. We decided to use the dada2 program for SV extraction for further analysis because dada2 identified a larger number of SVs than deblur, and some of the polymorphic nematode-derived SVs could be detected with dada2, but not with deblur.

**Table 2:** Number of nematode- and non-nematode-derived SVs detected by dada2 and deblur.

Method	Region	Number of nematode-derived SV (% in total SVs)	Number of non-nematode-derived SV	Total number of SVs
Dada2	Region 1	203 (36.6)	351	554
	Region 2	131 (37.2)	221	352
	Region 3	97 (61.4)	61	158
	Region 4	185 (36.2)	326	511
Deblur	Region 1	108 (37.8)	178	286
	Region 2	61 (34.4)	116	177
	Region 3	43 (50.0)	43	86
	Region 4	96 (34.2)	185	281

The phylum of the SVs was determined using the SILVA database in each nematode sample (sample ID in Fig 3) and shown in the histograms in regions 1–4 (Fig 3A–3D). The numbers of samples containing major SVs derived from the phylum Nematoda (over 50% in total SVs) were 67 (70.0% in total samples), 70 (72.9%), 72 (75.0%), and 68 (70.8%) out of the 96 samples from the four regions (Fig 3, purple fractions in the histograms). The major phyla derived from non-nematode organisms were “Ascomycota” (Fig 3, dark yellow fractions) and “Chordata” (Fig 3, dark green fractions). Interestingly, many of the samples that exhibited poor PCR amplifications contained SVs derived from non-nematode phylum. For example, 24 out of 27 samples that exhibited poor PCR amplifications (sample ID with red triangles in Fig 3), contained non-nematode-derived SVs (Fig 3). Additionally, it should be noted that fungi (the phyla Ascomycota and Basidiomycota)-derived DNAs were preferentially amplified in regions 1, 2, and 4 (Fig 3A, 3B and 3D), but not in region 3 (Fig 3C).

### Analysis of nematode-derived SVs in four regions and identification of the SSU-derived operational taxonomic units (rOTUs)

**Fig 4:**
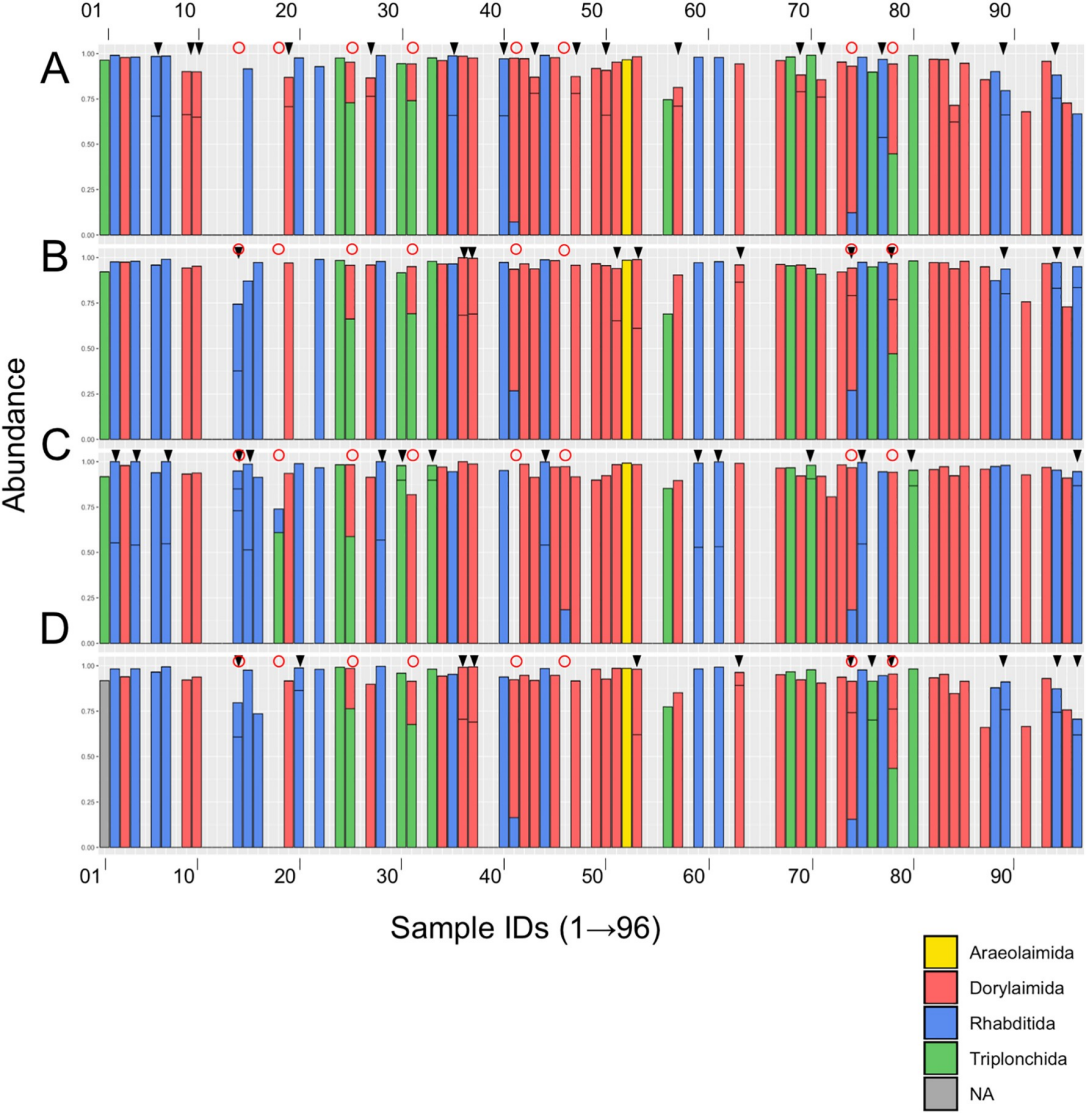
Histograms of nematode-derived SVs at the order level in the samples. Relative abundance and orders of nematode-derived SVs obtained from regions 1 (A), 2 (B), 3 (C), and 4 (D) in each sample are shown in the histogram by color. Classification of the nematode orders in each SV was based on the SILVA database. The sample ID numbers are aligned from left to right, and the colors corresponding to order are shown in a legend box. Black arrow heads and red circles at the top of the histograms indicate sample IDs containing possible polymorphic nematode-derived SVs and two different nematodes, respectively. NA: Not assigned.

Next, we selected the samples containing nematode-derived SVs according to the two following criteria: 1) the samples contained more than 1,000 nematode-derived sequence reads and 2) the relative abundance of nematode-derived SVs including polymorphic SVs was over 65% in total SVs. The number of the resultant independent samples with nematode-derived SVs was 68 (i.e., 64 samples in region 1, 65 in region 2, 67 in region 3, and 66 in region 4) (Fig 4), and the numbers of nematode-derived SVs were 21 in region 1, 23 in region 2, 21 in region 3, and 24 in region 4, respectively (S2 Table). A lower number of SVs contain more identical sequence reads (S2 Table). Since each sample contained a single nematode, the most abundant nematode-derived SVs should come from the nematode in the sample. Thus, we assigned regional SVs in each sample to a single nematode order using the SILVA database in order to identify the taxonomic rank of the nematode. We found that the majority of nematode-derived SVs were classified into the three major orders Dorylamida, Rhabditida, and Triplonchida (Fig 4). We also found that some of the samples (17 samples in region 1; 11 in region 2; 15 in region 3; 12 in region 4) contained two or three nematode-derived SVs as indicated by an arrow head at the top of the histograms (Fig 4). Seven samples (i.e., sample ID_18, 25, 31, 41, 46, 74, and 78), indicated by red circles, clearly contained two SVs derived from different orders. The second most abundant nematode-derived SVs (minor SVs) associated with the most abundant SVs (major SVs) were presumably derived from predation or contamination of the nematodes (or their debris). Regarding these seven samples, major SVs were used for the subsequent identification of the rOTUs. In contrast, the minor SVs in other samples were likely derived from polymorphic alleles in the 18S rRNA gene clusters, because the nucleotide sequences of a paired SVs contained only a few nucleotide-differences, and also the same pairs of major and minor SVs were found in multiple samples. For example, sample ID_09, 10, 19, 27, 43, 47, 50, 57, 69, 71 and 84 commonly contained R1_SV_3 and R1_SV_12 in region 1 (S2 Table) and these SVs shared the nucleotide sequences with one base-mismatch (A in SV_3, and G in SV_12) (Table 3). The sample ID_74 and 78 contained SVs from two different nematodes, and one of them contained a pair of polymorphic SVs in regions 2 and 4. Additionally, sample ID_14 contained two (regions 2 and 4) and three (region 3) SVs with distinct nucleotide sequences derived from the order Rhabditida. Since it was difficult to distinguish if this sample contained two related nematodes and/or polymorphic SVs, we removed sample ID_14 for further identification of the rOTUs. The major SV in each region was named as regional SV and designated by “the R region number_SV_number” such as R1_SV_1 (i.e., SV_1 isolated in region 1) (Table 3).

**Table 3:** Summary of nematode-derived rOTUs identified from deep amplicon sequencing in four SSU gene regions.

rOTU^a^	Regional rOTU^b^	Regional SV^c^	Allele^d^	No. of isolates	Sample ID belonging to rOTU^e^	Order^f^	Feeding types^g^
Z01rOTU01	Z01rOTU01_R1	R1_SV_2		13	SnTUT_z01_02, 04, 06, 07, 15, 28, 35, 40, 44, 59, 61, 75, 77	Rhabditida	Plant feeding (1d)
	Z01rOTU01_R2	R2_SV_2					
	Z01rOTU01_R3	R3_SV_4					
	Z01rOTU01_R4	R4_SV_1					
	Z01rOTU01_R1a^d^	R1_SV_13	A/G	4	SnTUT_z01_06, 35, 40, 77		
	Z01rOTU01_R3a	R3_SV_5	G/A	8	SnTUT_z01__02, 04, 07, 15, 28, 59, 61, 75		
Z01rOTU02	Z01rOTU02_R1	R1_SV_3		11	SnTUT_z01__09, 10, 19, 27, 43, 47, 50, 57, 69, 71, 84	Dorylaimida	Hyphal feeding (2)
	Z01rOTU02_R2	R2_SV_3					
	Z01rOTU02_R3	R3_SV_3					
	Z01rOTU02_R4	R4_SV_3					
	Z01rOTU02_R1a	R1_SV_12	A/G	11	SnTUT_z01__09, 10, 19, 27, 43, 47, 50, 57, 69, 71, 84		
Z01rOTU03	Z01rOTU03_R1	R1_SV_1		11	SnTUT_z01_03, 34, 42, 45, 67, 73, 82, 83, 85, 87, 93	Dorylaimida	Hyphal feeding (2)
	Z01rOTU03_R2	R2_SV_1					
	Z01rOTU03_R3	R3_SV_1					
	Z01rOTU03_R4	R4_SV_2					
Z01rOTU04	Z01rOTU04_R1	R1_SV_4		7	SnTUT_z01_36, 37, 46^h^, 53, 63, 74^h^, 78^h^	Dorylaimida	Animal predation (5) or Omunivorous (8)
	Z01rOTU04_R2	R2_SV_6					
	Z01rOTU04_R3	R3_SV_2					
	Z01rOTU04_R4	R4_SV_6					
	Z01rOTU04_R2a	R2_SV_11	T/A, G/A	6	SnTUT_z01_36, 37, 53, 63, 74^h^, 78^h^		
	Z01rOTU04_R4a	R4_SV_16	T/G	6	SnTUT_z01_36, 37, 53, 63, 74^h^, 78^h^		
Z01rOTU05	Z01rOTU05_R1	R1_SV_6		4	SnTUT_z01_30, 33, 70, 80	Triplonchida	Plant feeding (1d)
	Z01rOTU05_R2	R2_SV_5					
	Z01rOTU05_R3	R3_SV_6					
	Z01rOTU05_R4	R4_SV_5					
	Z01rOTU05_R3a	R3_SV_18	G/T	4	SnTUT_z01_30, 33, 70, 80		
Z01rOTU06	Z01rOTU06_R1	R1_SV_10		3	SnTUT_z01_89, 94, 96	Rhabditida	Plant feeding (1e)
	Z01rOTU06_R2	R2_SV_7					
	Z01rOTU06_R3	R3_SV_7					
	Z01rOTU06_R4	R4_SV_8					
	Z01rOTU06_R1a	R1_SV_31	T/C (2 sites), A/C	2	SnTUT_z01_89, 94		
	Z01rOTU06_R2a	R2_SV_22	T/C (4 sites)	3	SnTUT_z01_89, 94, 96		
	Z01rOTU06_R3a	R3_SV_23	C/T	1	SnTUT_z01_96		
	Z01rOTU06_R4a	R4_SV_31	A/T, GG insertion	3	SnTUT_z01_89, 94, 96		
Z01rOTU07	Z01rOTU07_R1	R1_SV_11		3	SnTUT_z01_72, 91, 95	Dorylaimida	Hyphal feeding (2)
	Z01rOTU07_R2	R2_SV_10					
	Z01rOTU07_R3	R3_SV_8					
	Z01rOTU07_R4	R4_SV_10					
Z01rOTU08	Z01rOTU08_R1	R1_SV_7		2	SnTUT_z01_41^h^, 51	Dorylaimida	Animal predation (5) or Omunivorous (8)
	Z01rOTU08_R2	R2_SV_9					
	Z01rOTU08_R3	R3_SV_2					
	Z01rOTU08_R4	R4_SV_12					
	Z01rOTU08_R2a	R2_SV_23	C/T, C deletion	1	SnTUT_z01_51		
Z01rOTU09	Z01rOTU09_R1	R1_SV_8		2	SnTUT_z01_31^h^, 76	Triplonchida	Bacterial feeding (3) or Unicellular eukaryote feeding? (6?)
	Z01rOTU09_R2	R2_SV_8					
	Z01rOTU09_R4	R4_SV_13					
	Z01rOTU09_R4a	R4_SV_35	C/A	1	SnTUT_z01_76		
Z01rOTU10	Z01rOTU10_R1	R1_SV_9		2	SnTUT_z01_20, 88	Rhabditida	Bacterial feeding (3)
	Z01rOTU10_R2	R2_SV_27					
	Z01rOTU10_R3	R3_SV_9					
	Z01rOTU10_R4	R4_SV_15					
	Z01rOTU10_R4a	R4_SV_64	C/T	1	SnTUT_z01_20		
Z01rOTU11	Z01rOTU11_R1	R1_SV_23		2	SnTUT_z01_16, 22	Rhabditida	Plant feeding (1b)
	Z01rOTU11_R2	R2_SV_13					
	Z01rOTU11_R3	R3_SV_11					
	Z01rOTU11_R4	R4_SV_23					
Z01rOTU12	Z01rOTU12_R1	R1_SV_25		2	SnTUT_z01_18^h^, 24	Triplonchida	Bacterial feeding? (3?)
	Z01rOTU12_R2	R2_SV_18					
	Z01rOTU12_R3	R3_SV_14					
	Z01rOTU12_R4	R4_SV_19					
Z01rOTU13	Z01rOTU13_R1	R1_SV_15		1	SnTUT_z01_49	Dorylaimida	Omunivorous? (8?)
	Z01rOTU13_R2	R2_SV_15					
	Z01rOTU13_R3	R3_SV_2					
	Z01rOTU13_R4	R4_SV_21					
Z01rOTU14	Z01rOTU14_R1	R1_SV_16		1	SnTUT_z01_68	Triplonchida	Hyphal feeding (2)
	Z01rOTU14_R2	R2_SV_14					
	Z01rOTU14_R3	R3_SV_13					
	Z01rOTU14_R4	R4_SV_18					
Z01rOTU15	Z01rOTU15_R1	R1_SV_18		1	SnTUT_z01_52	Plectida	Bacterial feeding (3)
	Z01rOTU15_R2	R2_SV_16					
	Z01rOTU15_R3	R3_SV_12					
	Z01rOTU15_R4	R4_SV_22					
Z01rOTU16	Z01rOTU16_R1	R1_SV_19		1	SnTUT_z01_01	Triplonchida	Bacterial feeding (3) or Unicellular eukaryote feeding? (6?)
	Z01rOTU16_R2	R2_SV_17					
	Z01rOTU16_R3	R3_SV_16					
	Z01rOTU16_R4	R4_SV_24					
Z01rOTU17	Z01rOTU17_R1	R1_SV_20		1	SnTUT_z01_56	Triplonchida	Bacterial feeding? (3?)
	Z01rOTU17_R2	R2_SV_19					
	Z01rOTU17_R3	R3_SV_15					
	Z01rOTU17_R4	R4_SV_25					
Z01rOTU18	Z01rOTU18_R1	R1_SV_27		1	SnTUT_z01_25^h^	Triplonchida	Bacterial feeding (3) or Unicellular eukaryote feeding? (6?)
	Z01rOTU18_R2	R2_SV_20					
	Z01rOTU18_R3	R3_SV_17					
	Z01rOTU18_R4	R4_SV_29					

^a^Code “Z01” represents the experimental code of this study. Two-digit serial numbers were assigned in the order of the number of nematodes assigned to the rOTU.

^b^Regional rOTU is defined as the rOTU in each region and described as “rOTU with the R region number such as R1”.

^c^Regional SV is the sequence variant corresponding to the regional rOTU and described as “the R region number_SV number” such as R1_SV_2.

^d^The regional OTUs with polymorphic alleles were also shown as the corresponding rOTU name with an alphabet such as Z01rOTU01_R1a. Different nucleotide sequences between the corresponding regional rOTU and the polymorphic regional rOTU were shown, such as A/G (A in the corresponding regional rOTU and G in the polymorphic allele).

^e^Sample ID was named as SnTUT_z01_a two-digit serial number.

^f^Order of each rOTU was assigned based on the phylogenetic trees in Figs 5–8.

^g^Feeding types were derived from those of the closest species to the rOTU except for Z01rOTU09 in the phylogenetic tree R1_2_3_4 (Fig 9), according to the reference in Yeats et al. (1993) [42]. The assignment of feeding type for Z01rOTU09 was based on the tree R1_2 (S5 Fig). Numbers in parentheses indicated feeding types in the reference.

^h^Isolates containing two taxonomically different nematodes.

The nematode isolates sharing the identical nematode-derived SVs were clustered in each region, and the resultant 18 rOTUs were obtained (Table 3 and S2 Table). These rOTUs were named Z01rOTU01–Z01rOTU18 in the order of the numbers of isolates belonging to each rOTU. Z01 represents our experimental code for this study and each nematode isolate in each rOTU was named SnTUT_z01_sample ID according to the nomenclature system in our previous study [19]. The largest Z01rOTU01 included 13 nematodes and the six smallest rOTUs, Z01rOTU13–Z01rOTU18, were derived from a single isolate. The regional SVs and their associated polymorphic SVs belonging to each rOTU were also defined as regional rOTUs and regional polymorphic rOTUs, and indicated as “rOTU name_R region number (plus an alphabet for a polymorphic allele)” such as Z01rOTU01_R1 and Z01rOTU_R1a, respectively. Thus, a rOTU consisted of four regional rOTUs and a few polymorphic regional rOTUs (Table 3). The numbers of polymorphic rOTUs found in each region were almost comparable: 3, 3, 3, and 4 in regions 1–4 (Table 3).

### Phylogenetic analysis of nematodes belonging to Z01rOTUs in four regions of SSU gene

Although taxonomic rank information for the Z01rOTUs was obtained from the SILVA database, we found that lower taxonomic ranks under the order were markedly poor (S3 Table). Therefore, we next performed the BLASTN search against the non-redundant nucleotide sequence database using regional Z01rOTUs as query sequences to obtain taxonomic information for the Z01rOTUs in detail. The orders, families, and genera of the most homologous nematode species for each Z01rOTU were identified (S4 Table). Single orders were consistently determined by four regional OTUs of 13 Z01rOTUs except for Z01rOTU09, 14, 15, 16, and 18. Because the inconsistent order Dorylamida in 3 of 5 Z01rOTUs (i.e., Z01rOTU09, 14 and 16) came from *Diphterophora communis*, which should be classified into Triplonchida as described in the next section, the consistent orders for 16 out of 18 Z01rOTUs were finally determined. The orders assigned to the regional Z01rOTUs based on the SILVA database almost agreed with those detected from the BLASTN search except for Z01rOTU15, which was assigned to a different order (Araeolamida versus Plectida) by each analysis (S3 and S4 Tables). Next, we assigned the families and genera of Z01rOTUs based on the BLAST-best hit species. Single families were determined in 9 Z01rOTUs (01, 05, 06, 07, 09, 10, 11, 12, and 17) and 7 of them, except for Z01rOTU10 and 11, were successfully assigned to single genera in 4 regions (3 in Z01rOTU09) (S4 Table). Three Z01rOTUs (14, 16, and 18) were assigned to two families, and four regional rOTUs of Z01rOTU15 commonly shared the family Plectidae despite the occasional inclusion of two other families (Dorylamida and Rhabditida); however, it was difficult to assign the remaining 5 Z01rOTUs (02, 03, 04, 08, and 13) to defined families (S4 Table). Additionally, we tried to increase the sensitivity and specificity of the BLAST search using long sequences that were artificially generated by connecting four regional rOTU sequences. However, the results were similar to those from the homology search using regional short sequences; or instead, more variable orders, families, and genera were identified in some Z01rOTUs from the search using long sequences than those from the search using short sequences (S4 Table). Additionally, throughout the BLAST search, Z01rOTU03 and Z01rOTU16 turned to be identical to K01rOTU12 (AB728436) and H01rOTU10 (AB728367 and AB728366), respectively, which were derived from nematodes isolated from the flower bed and field soils in our previous study [19].

Furthermore, we have assigned the taxonomic status of nematodes belonging to Z01rOTUs by using the phylogenetic tree built by the SSU sequences from the reference species and Z01rOTUs, because the phylogenetic trees of the phylum Nematoda have been already built and modified by several groups [29, 43, 44]. We selected 116 reference nematode species and *Halobiotus crippae* (phylum Tardigrada) as the outgroup (S1 Table) and prepared their nucleotide sequences corresponding to regions 1–4 of the SSU gene. In principle, one reference species per nematode family, whose full-length sequences of SSU genes are publicly available in GenBank, was selected for phylogenetic analysis. Ten families were omitted due to unavailable full-length sequences or unsuccessful clustering, and 8 out of 57 families were used from the suborder Spirurina.

First, we prepared the reference phylogenetic tree with the neighbor-joining method using full-length sequences of the 18S rRNA gene from 116 taxonomically known nematode species, whose orders were classified by colored dots (S4 Fig). According to the taxonomy of the phylum Nematoda [29], Nematoda consists of two classes Chromadorea and Enoplea, and the class Enoplea contains two subclasses Enoplia and Dorylamina. The resultant cladogram contains species belonging to 17 orders (Araeolaimida [3 species], Chromadorida [4 species], Monhysterida [5 species], Desmodorida [5 species], Desmoscolecida, Plectida [10 species], Rhabditida [37 species], and Strongylida [4 species] in the class Chromadorea; Dioctophymatida, Enoplida [13 species], Isolaimida, Mermithida, and Triplonchida [8 species] in the subclass Enoplia; Dorylaimida [13 species], Mononchida [5 species] and Trichinellida [4 species] in the subclass Dorylamina). Species belonging to the order Rhabditida were further classified into two suborders: Spirurina (8 species) and Tylenchina (16 species), and other Rhabditida (13 species). Four orders (Desmodorida, Dioctophymatida, Isolaimida, and Mermithida) contained a single species. Two nematode orders of Muspiceida and Rhaptothyreida were omitted due to unavailable full-length sequences. The order Rhabditida contains the largest number of 37 species, coming from the same number of families. Species belonging to each order have clustered to single groups in the tree; however, a few of the members in the orders Araeolaimida, Desmodorida, Monhysterida, and Plectida were distributed to separated clusters (S4 Fig). Additionally, *Diphterophora communis* was classified into the order Dorylaimida (taxonomy ID: 288632) in current the NCBI database (April 2020); however, the nucleotide sequence (AY593955) was assigned to a cluster of Triplonchida in our phylogenetic tree (S4 Fig), and the BLASTN search using this sequence also hit many nematode species belonging to the order Triplonchida. In addition, van Megen et al. assigned this species to a cluster of Triplonchida in their recent SSU-based phylogenetic tree [44]. Thus, we counted this species as Triplonchida in this study.

**Fig 5:**
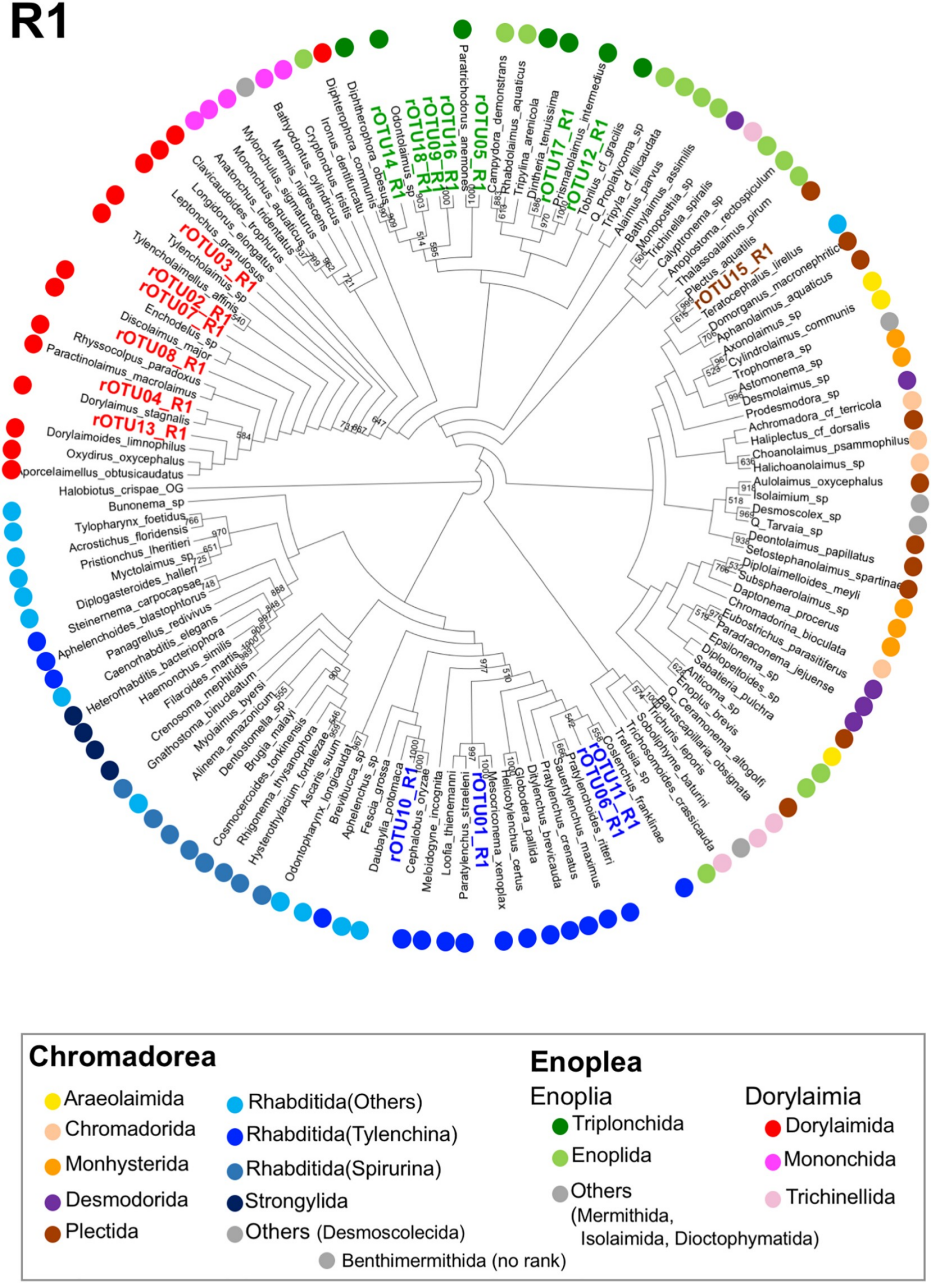
A cladogram of phylogenetic tree generated by regional SSU nucleotide sequences from reference nematode species and regional rOTUs corresponding to region 1. The phylogenetic tree was prepared using regional sequences of the reference nematode species and the regional rOTUs corresponding to region 1 (R1) as described in Materials and methods. Orders of the reference species are indicated by colored dots, as shown in the legend box. Each regional rOTU in the cladograms is indicated by colored letters corresponding to the order’s color.

**Fig 6:**
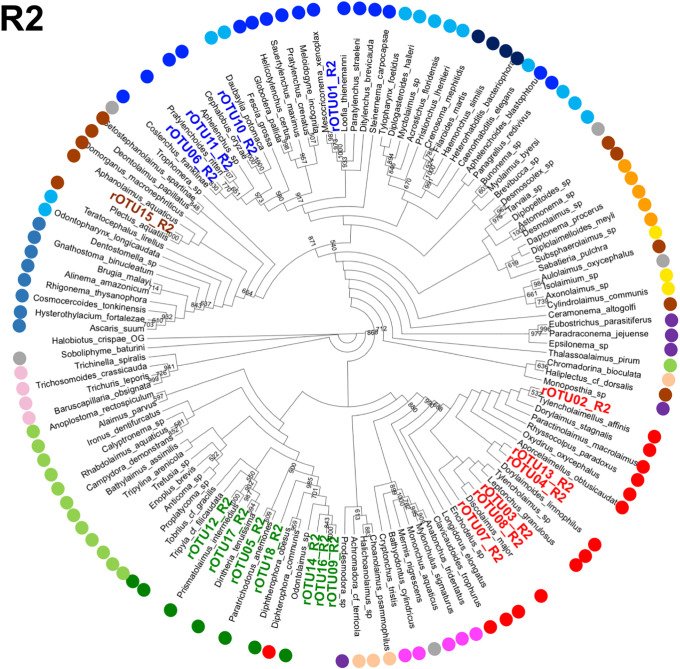
A cladogram of phylogenetic tree generated by regional SSU nucleotide sequences from reference nematode species and regional rOTUs corresponding to region 2. The phylogenetic trees were prepared using regional sequences of the reference nematode species and the regional rOTUs corresponding to region 2 (R2) as described in Materials and methods. Orders of the reference species are indicated by colored dots, as shown in the legend for Fig 5.

**Fig 7:**
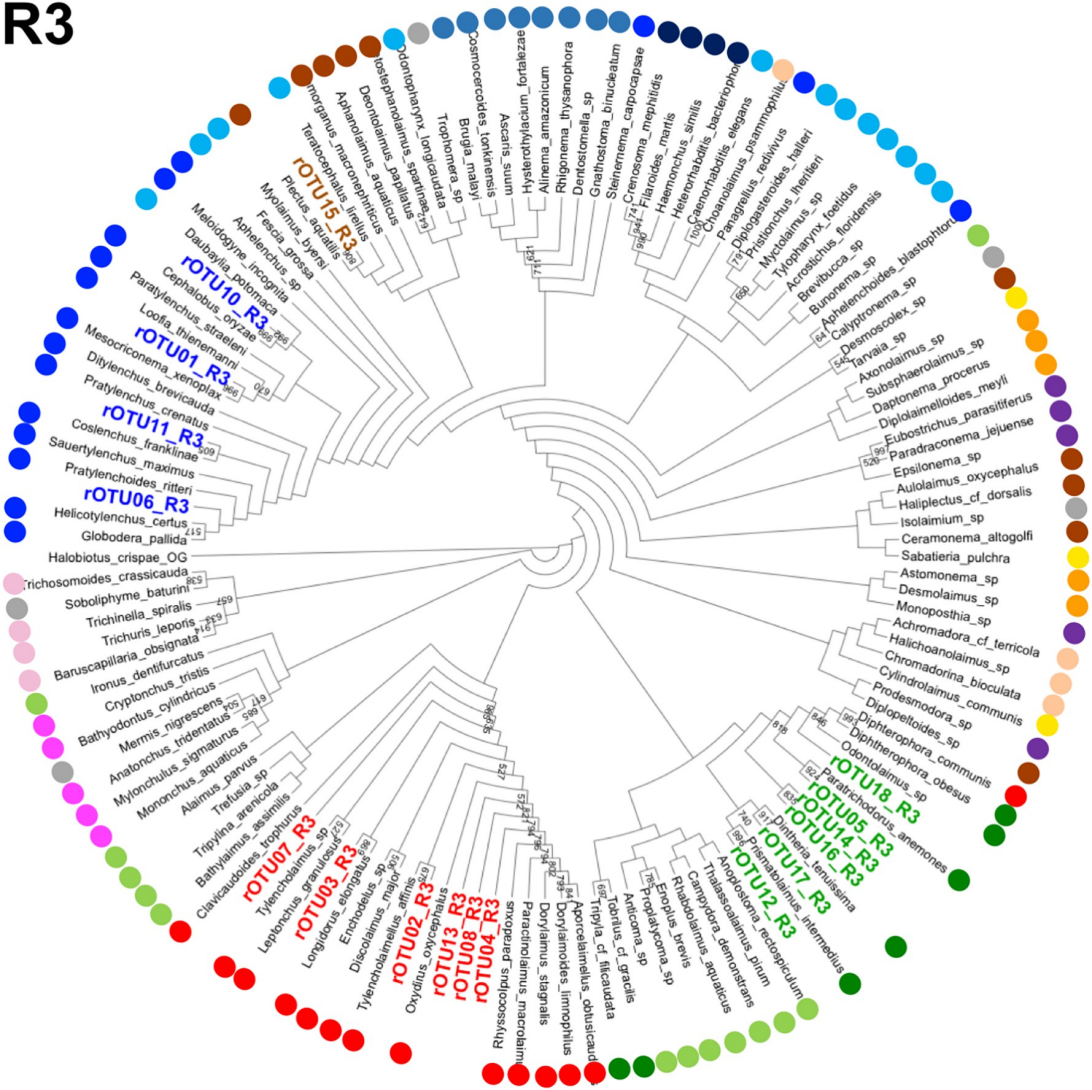
A cladogram of phylogenetic tree generated by regional (region 3) SSU nucleotide sequences from reference nematode species and regional rOTUs. The phylogenetic trees were prepared using regional sequences of the reference nematode species and the regional rOTUs corresponding to region 3 (R3) as described in Materials and methods. Orders of the reference species are indicated by colored dots, as shown in the legend for Fig 5.

**Fig 8:**
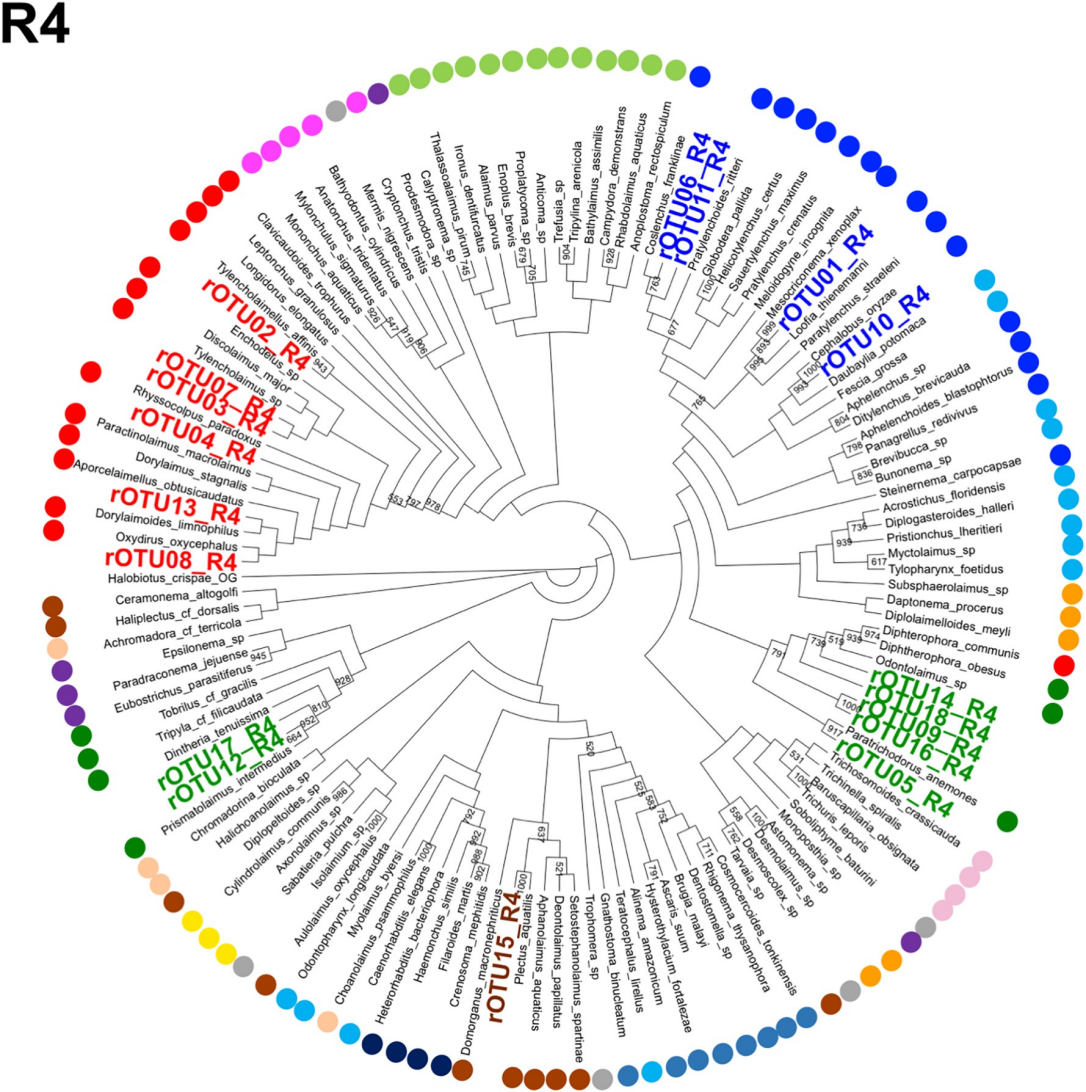
A cladogram of phylogenetic tree generated by regional (region 4) SSU nucleotide sequences from reference nematode species and regional rOTUs. The phylogenetic trees were prepared using regional sequences of the reference nematode species and the regional rOTUs corresponding to region 4 (R4) as described in Materials and methods. Orders of the reference species are indicated by colored dots, as shown in the legend for Fig 5.

Next, we assigned the Z01rOTUs on the phylogenetic tree using each regional Z01rOTU and the corresponding nucleotide sequences from the reference species. The resultant four cladograms in the regions (designated as R plus the region number, such as R1) indicated that 18 regional Z01rOTUs were commonly clustered into four taxonomic groups (R1–R4 in Figs 5–8): 35 isolates (51.5% in total isolates) derived from Z01rOTU02, 03, 04, 07, 08, and 13 were assigned to the order Dorylaimida, 20 nematodes (29.4%) from Z01rOTU01, 06, 10, and 11 were to the order Rhabditida, 12 isolates (17.6%) from Z01rOTU05, 09, 12, 14, 16, 17, and 18 were to the order Triplonchida, and one isolate (1.5%) from Z01rOTU15 was to the order Plectida (Table 3). Although similar structures of taxonomic clusters were obtained in the resultant trees, different branch structures among the cladograms were found in the orders Enoplida and Rhabditida. For instance, species from the order Enoplida were separated into 6, 2, 4, and 1 clusters in the tree of regions 1–4, respectively.

**Fig 9:**
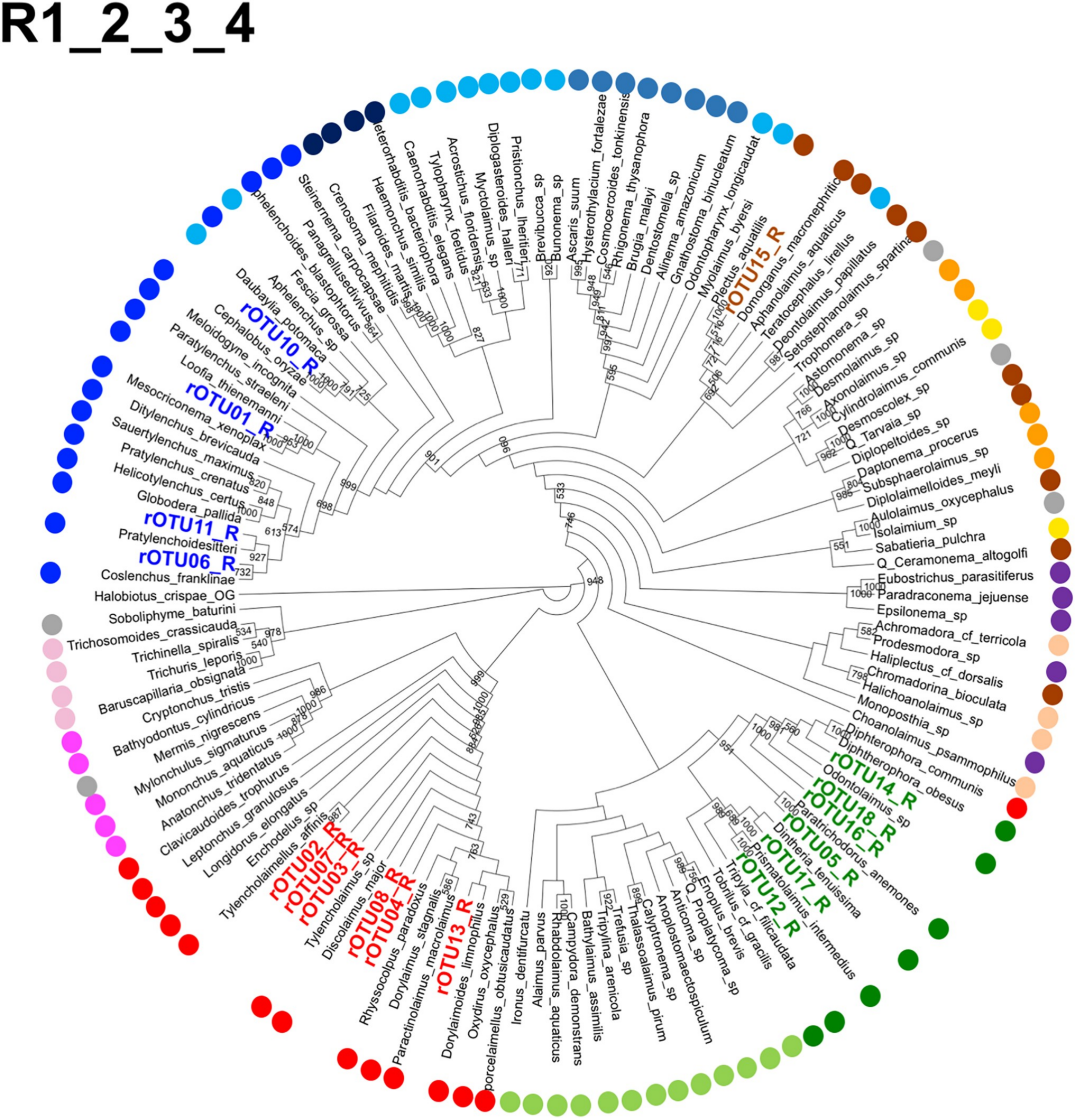
A cladogram of phylogenetic tree generated by combined SSU nucleotide sequences from reference nematode species and rOTUs. The phylogenetic tree was prepared using regional sequences of the reference nematode species and the regional rOTUs corresponding to the combined sequences of the four regions in numerical order (R1_2_3_4), as described in Materials and methods. Orders of the reference species are indicated by colored dots, as shown in the legend for Fig 5.

Therefore, we counted the numbers of order clusters (Table 4) as well as the largest number of species in the clusters (S5 Table) in the tree to evaluate the resultant phylogenetic trees in detail. Nematode species derived from four orders (Dorylaimida, Mononchida, Strongylida, and Trichinellida) gathered nicely into single clusters in the trees except for Trichinellida in region 1. However, species belonging to the other orders were distributed into multiple branches (Table 4). Since the number of branches in the trees built by regional short sequences trended to slightly increase by comparing them to those in the tree with full-length sequences (R1–R4 in Figs 5–8 and S4 Fig), we have presumed potential improvement of branch structures of the phylogenetic tree by using long sequences. Thus, we prepared long nucleotide sequences of the SSU gene by connecting regional sequences to make phylogenetic trees. The four cladograms (R1_2, R3_4, R2_3_4, and R1_2_3_4) were prepared using artificially combined sequences of the indicated region numbers (S5 and S6 Figs and Fig 9). For example, R2_3_4 means the sequence connected the region 2, region 3, and region 4 sequences in order. In these trees, the structures of the clusters of species from the orders Enoplida and Triplonchida were clearly improved by using long SSU gene sequences. For example, although 13 Enoplida species were distributed into 6 clusters in the tree of region 1 (R1), they clustered to a single group in the phylogenetic tree of R1_2_3_4 (Fig 9). In contrast, the species belonging to the other six orders (Araeolaimida, Chromadorida, Desmodorida, Monhysterida, Plectida, and Rhabditida) barely gathered to single clusters in the trees. In addition, we further investigated the phylogenetic trees using reference sequences alone in order to examine the possible interference in the presence of Z01rOTU sequences to the preparation of phylogenetic trees using short or long SSU sequences (S7–S10 Figs). The similar results of cluster numbers were obtained from these phylogenetic trees only containing regional or their connected sequences from the reference species (Table 4). The largest numbers of clusters in each tree also supported this result (S5 Table).

**Table 4:** Numbers of clusters in the phylogenetic trees built by regional and artificially combined SSU sequences.

Order^a^	Total number^b^	R1^c^	R2	R3	R4	R1_2^c^	R3_4	R2_3_4	R1_2_3_4	Reference (Full length)^d^
**A. Trees built by regional reference and Z01rOTU sequences**										
Araeolaimida	3	2	2	3	1	2	2	2	2	2
Chromadorida	4	3	2	2	3	2	2	3	3	2
Desmodorida	5	3	3	3	3	3	2	3	3	3
Dorylaimida	13	1	1	1	1	1	1	1	1	1
Enoplida	13	6	2	4	1	2	2	2	1	1
Monhysterida	5	2	1	2	2	2	3	1	2	3
Mononchida	5	1	1	1	1	1	1	1	1	1
Plectida	10	6	5	5	5	5	5	3	6	4
Rhabditida	37	3	3	6	4	2	4	3	3	3
Strongylida	4	1	1	1	1	1	1	1	1	1
Trichinellida	4	2	1	1	1	1	1	1	1	1
Triplonchida	8	2	1	2	2	1	1	1	1	1
**B. Trees built by regional reference sequences alone**										
Araeolaimida	3	2	2	3	1	2	2	2	2	2
Chromadorida	4	2	2	3	4	2	3	3	3	2
Desmodorida	5	3	3	2	3	3	2	3	3	3
Dorylaimida	13	1	1	1	1	1	1	1	1	1
Enoplida	13	5	3	4	1	2	3	1	2	1
Monhysterida	5	2	2	2	3	2	3	1	1	3
Mononchida	5	1	1	2	1	1	1	1	1	1
Plectida	10	4	5	4	4	5	4	4	5	4
Rhabditida	37	2	3	4	4	3	4	4	3	3
Strongylida	4	1	1	1	1	1	1	1	1	1
Trichinellida	4	2	1	1	1	1	1	1	1	1
Triplonchida	8	3	1	3	1	1	2	1	1	1

The number of clusters of species belonging to each order was counted in the phylogenetic tree obtained from regional reference and Z01rOTU sequences (A) as well as regional reference sequences alone (B) (Figs 5–9, S5–S10 Figs).

^a^Orders of the reference species were derived from the taxonomic data of the NCBI database. Five orders containing single reference species (Benthimermithida, Desmoscolecida, Dioctophymatida, Isolaimida, and Mermithida) were omitted. *Diphterophora communisis* was assigned to the order Triplonchida, as described in the text.

^b^The total number of species in each order was indicated.

^c^The SSU regions of the regional and combined sequences used for preparing phylogenetic trees were shown as R plus the region numbers. For example, R2_3_4 represents the phylogenetic tree built by the nucleotide sequence connected to the regional sequences in the order of regions 2, 3, and 4.

^d^Full-length sequences from the reference nematode species (S1 Table) were used for the preparation of the phylogenetic tree as a control (S4 Fig).

Finally, we predicted the feeding types of isolates belonging to the Z01rOTUs based on the reference nematode species, which have the closest sequence similarities in the phylogenetic trees by referring to the previous study [42]. The feeding types of isolates belonging to 17 Z01rOTUs were predicted using the phylogenetic tree of R1_2_3_4 (Fig 9), and the tree of R1_2 was used for Z01rOTU09 due to the lack of regional rOTU_R3 (S5 Fig). The predicted feeding types of nematode isolates corresponding to 18 Z01rOTUs were hyphal feeders (fungivores) (26 isolates [38.2% in total 68 nematodes] of Z01rOTU02, 03, 07, and 14), plant feeders (plant parasites) (22 isolates [32.4%] of Z01rOTU01, 05, 06, and 11), bacterial feeders (bacterivores) (10 isolates [14.7%] of Z01rOTU09, 10, 12, 15–18), and predators or omnivores (10 isolates [14.7%] of Z01rOTU04, 08, and 13), respectively.

## Discussion

### NGS-based DNA barcoding of nematodes in four SSU regions

We investigated four target regions (regions 1–4) of the SSU gene for DNA barcoding of nematodes using deep amplicon sequencing on the Illumina MiSeq platform. In our previous study [19], we used a long SSU-derived amplicon of approximately 900-bp spanning regions 1 and 2 (V1–V4 regions) via Sanger sequencing (Fig 1B) and the resultant long sequences provided us with sufficient taxonomic resolutions. However, in this study, we needed to generate four tailed and sequence-improved primer sets by amplifying relatively short (300–400 bp) DNAs in order to adapt to the Illumina sequencing reaction. Despite its high-throughput sequence analysis, decreased PCR success rate and taxonomic resolution were potential concerns that could be caused using long tailed primers and short DNA barcode sequences. These four regions almost covered the entire SSU gene and spanned at least one hypervariable sequence region (region 1, V1–V2; region 2, V4; region 3, V5–V7; and region 4, V7–V8) [38] (Fig 1B). Previous studies of NGS-based nematode barcoding preferentially used PCR amplicons generated from the 5’-region of the SSU gene (region 1) with a primer set of SSUF04 and SSUR22 [26–28, 41] and/or the 3’-SSU region (region 4) with NF1 and 18Sr2b primer sets [20–23, 25, 39]. Although the comparable numbers of nematode-derived SVs were obtained in both regions from our dada2-mediated analysis (i.e., 203 in region 1 and 185 in region 4) (Table 2), we found insufficient sequence coverage of nematode reference species in region 1 in the public database upon selection of the reference nematode species for phylogenetic analysis. Indeed, we could not find complete SSU sequences in at least 12 out of 126 nematode families that we initially selected due to the partially truncated region 1. Recently, Ahmed et al. also showed that the coverage of nematode species in the SSUF04-SSUR22 region (region 1) was much less than that in the NF1-18Sr2b region (region 4), suggesting that region 4 offers wide coverage and excellent taxonomic resolution for characterizing soil nematodes [40]. In contrast, the small numbers of nematode-derived SVs were detected in regions 2 and 3 by dada2 and deblur algorithms (Table 2); particularly, half of the numbers of SVs found in regions 1 and 4 were only obtained in region 3. The PCR success rate of region 3 (61.5%) was also lower than those in the other three regions (70–74%); however, the relative abundance of nematode-derived SVs occupied in the total SVs was significantly high (61.4 and 50.0% in dada2 and deblur, respectively) compared with other regions (approximately 35%). Additionally, high abundance of fungi-derived SVs in non-nematode-derived SVs was observed in regions 1, 2, and 4; however, fungi-derived SVs were significantly decreased in region 3 (Fig 3). These data indicate that the primer set for region 3 can preferentially amplify nematode DNAs rather than PCR primer sets for other regions. Hugerth and colleagues investigated suitable hypervariable regions of the 18S rRNA gene for PCR amplicon-based eukaryotic community analysis and showed that the V4 and V5 regions are the most informative [38]. Hadziavdic et al. also characterized PCR primer sets of the 18S rRNA gene and suggested that the V2, V4, and V9 regions are best suited for biodiversity assessment [45]. Although both studies commonly indicated the informative V4 region in amplicon-based community analysis, the numbers of nematode-derived SVs detected in region 2 spanning the V4 region, were less than those in regions 1 and 4 (Table 2). Finally, the largest number of 24 nematode-derived SVs were obtained from region 4. Taken together, our results clearly suggest that a primer set of region 4 is most suitable for DNA barcode-based taxonomic classification of individual nematodes.

Progress of DNA metabarcoding for complex eukaryotic communities requires improved PCR primer sets for the amplification of SSU genes, which can cover a broad amount of species and provide rich taxonomic information. Although the DNA barcodes of the internal transcribed spacer (ITS)-2 [46], large subunit 28S rRNA gene [20], and cytochrome oxidase *c* subunit 1 gene [47] have been used, these have technical issues to be resolved. Some obstacles include insufficient numbers of reference sequences or biased PCR amplifications due to diverged mitochondrial DNA sequences [19, 40]. So far, two SSU primer sets (NemF-18Sr2b and EcoF-EcoR) have been reported for NGS-based DNA metabarcoding of nematode communities (Fig 1B). The former is an improved version of NF1-18Sr2b and can preferentially amplify nematode-derived SSU fragments (525 bp) from soil DNAs [21]. Waeyenberge et al. generated the latter primers covering the V4 and V5 regions of the SSU gene (532 bp in length); however, it showed less taxonomic coverage comparing with the adopted primer set of NemFopt-18Sr2bopt [24]. Future steps could test the four primer sets for DNA metabarcoding of nematodes using whole DNAs prepared from various types of soils.

### Detection of SVs and taxonomic analyses of nematode isolates from copse soils

We isolated 96 nematodes from copse soils and analyzed their taxonomic status by deep sequencing amplicons from four different SSU regions (regions 1–4). Throughout the analysis of SVs, we found that 27 out of 96 samples contained non-nematode-derived SVs mainly from the phyla Ascomycota and Chordata (Fig 3), and poor PCR amplifications were commonly observed in most of these samples (24 samples) (Fig 3 and S1 Fig). These poor quality PCRs may have been caused by the loss of nematode samples or nematode DNAs, and the resultant side-amplifications could have been contaminated with the DNAs of soil fungi and/or humans during sample manipulation.

We identified 21–24 of the major nematode-derived SVs, which included 3–4 polymorphic nematode-SVs from 64–67 samples in the four regions. The resultant 18 rOTUs were identified by clustering 68 nematode isolates using their nematode-derived SVs (Table 3). Throughout the analysis of nematode-derived SVs, we successfully identified polymorphic alleles in four regions. In our previous study, we determined the nucleotide sequences of cloned 900-bp DNAs via Sanger sequencing, and we identified 6 and 3 rOTUs containing polymorphic alleles out of 15 and 13 rOTUs derived from nematodes in an unmanaged flower bed and agricultural field soils, respectively [19]. A comparable number of 5 polymorphic rOTUs were identified in 18 Z01rOTUs in regions 1 and 2, which corresponded to our previous DNA barcode region (Fig 1B). We tried to detect these alleles using Sanger sequencing of PCR amplicons and found minor peaks derived from a few alleles in the corresponding nucleotide positions; however, several allelic sites failed to be clearly detected, suggesting the usefulness of NGS-based DNA barcoding for its sensitive and comprehensive detections of allelic sequences. Additionally, we found seven samples containing two taxonomically different nematodes (Fig 4). Interestingly, four samples (i.e., sample ID_41, 46, 74, and 78) contained nematodes belonging to Z01rOTU04 or Z01rOTU08 as major SVs, whose feeding types were predicted to be predacious or omnivorous (Table 3). Since this observation likely indicates that minor SVs were derived from the nematodes (i.e., 3 nematodes in Z01rOTU06 and 1 in Z01rOTU09) (S2 Table) predated by animal predators represented by major SVs, NGS-based DNA barcoding of individual nematodes is also a powerful tool to study nematode predation [48].

Upon isolation of the nematodes, we found morphologically distinctive ring nematodes during the microscopic observations (Fig 2). Seven of 11 ring nematodes belonged to members of Z01rOTU01 in our study (Table 3), and the BLAST search queried by the corresponding SV sequences hit the SSU gene from *Mesocriconema xenoplax* in the family Circonematidae (the order Rhabditida) at the highest e-value (S4 Table). This result was consistent with their morphological observation. *M*. *xenoplax* has been known to be widely distributed throughout the world and is known as one of the representative plant-parasitic nematodes that damage trees in the orchards and cause severe reductions in fruit production [49]. Our observation indicates that this species is abundant in copse soil in Japan as well.

In this study, we found that the nematode community in copse soils was mainly composed of nematodes derived from three orders, Dorylaimida, Rhabditida, and Triplonchida; particularly, approximately 50% of isolates were derived from the order Dorylaimida. Previous studies in various ecosystems have indicated a close relationship between nematode trophic groups and soil environments [5–7, 14]. The predicted feeding types of nematodes belonging to 18 Z01rOTUs from the copse soils were fungivores (4 Z01rOTUs, 38.2% in total nematodes), plant parasites (4 Z01rOTUs, 32.4%), bacterivores (7 Z01rOTUs, 14.7%), and predators or omnivores (3 Z01rOTUs, 14.7%), respectively (Table 3), indicating abundant proportions of fungivores and plant feeders in the nematode community in the copse soils of our campus. This result is markedly different from our previous observations obtained from cultivated field and unmanaged flower bed soils on our campus [19]: we found abundant bacterivores in the field soils, and plant feeders and predators dominated the flower bed soils. These observations clearly indicate different environmental and biological conditions of copse soils from those of the field and flower bed soils. Pothula and colleagues have examined the impact of agricultural intensification and urbanization on soil nematode communities using 111 published articles and examined the richness and abundance of 5 trophic groups in 5 different environmental soils including forest [50], indicating that the order of abundance was herbivores (plant feeders) > bacterivores > fungivores and omnivores > predators despite high variation. So far, several taxonomic studies on soil nematode communities in forest soils have been known and were performed using morphogenic and sequence-based approaches over the world (Brazil [51], Canada [52], China [53–56], Germany [57], Japan [58, 59], Slovakia [60], Sweden [61], USA [62]). These studies have shown that bacterial feeders, plant feeders, and fungivores often occupied significant fractions of the nematode communities in forest soils. A relatively high abundance of bacterial feeders has been commonly found; however, their proportions varied by the sampling sites and periods [58, 62], age [56], and environmental status of the forests [25, 52, 55] or treatments such as clear-cut harvesting and fertilization [61, 63]. Omnivores and predators were found in relatively minor fractions of soil nematodes in forest soils except in natural re-establishing subtropical forest [54] and coastal fir and pine forests [51, 58]. In contrast, our results showed that fungal and plant feeders dominated and the abundance of bacterial feeders significantly decreased in the nematode community in copse soils. It has been known that the taxonomic compositions of the soil nematode community were changed by environmental and nutrient factors including soil moisture, pH and/or food. Particularly, food is a very crucial factor governing the abundance of nematode trophic groups via complex food webs in various soils [64], for example, bacterivores are abundant in bacteria-rich soils such as cultivated fields and plant feeders dominate in grassland soils. Thus, plant feeder abundance in the copse soils can be explained by the plant-rich condition in the copse (Fig 1A). A surface of the sampling site was covered by weeds and litter, where the soils could maintain relatively high moisture content. Under this condition, fungi could propagate abundantly and occupy a significant fraction of the eukaryotic community, which may account for the abundance of fungal feeders in the copse soils. In addition, low abundance of bacterivores may suggest small amounts of bacteria due to poor nutrients in the soils because the abundance of bacteria trends to reflect soil nutrient status. These interpretations could be confirmed by future metagenomic and chemical analyses using copse soils.

Finally, we prepared phylogenetic trees using short sequences of the four SSU regions to assign Z01rOTUs to taxonomic groups (R1–R4 in Figs 5–8). Here, since short sequences from amplicon analysis have been believed to be less informative than long sequences for nucleotide variations-based taxonomic analyses, we tried to increase the taxonomic correctness of phylogenetic analysis of Z01rOTUs by using artificially generated long SSU sequences from the reference nematode species and rOTUs (S5 and S6 Figs). We evaluated the resultant phylogenetic trees by counting the numbers of clusters (Table 4) as well as the maximum numbers of species in the clusters in each order (S5 Table). Although the clustering of Enoplida species clearly improved, the clusters of the other orders’ species were not significantly changed in both criteria (Table 4 and S5 Table), which may suggest the limitations of this approach. In addition, we tested this approach to taxonomic assignments using the SILVA database as well as the BLAST search; however, the former analysis gave poor taxonomic resolution (S3 Table), and the results from the latter homology search were comparable or became more complicated due to increased (or contaminated) hit species (S4 Table).

## Conclusions

In this study, we investigated four regions of the SSU gene to find PCR target regions for DNA barcoding of individual soil nematodes. Region 4, located at the 3’-region of the SSU gene, was most suitable among the tested regions due to the identification of the most abundant nematode-derived SVs and sufficient reference sequence coverage. In contrast, the primer set for region 3 relatively amplified DNAs in a nematode-specific manner. For the extraction of SVs, we tested the dada2 and deblur algorithms, and a larger number of SVs, including SSU variant sequences, were obtained using dada2. These observations indicate that the Illumina MiSeq-assisted DNA barcoding using the PCR primer set amplifying region 4 of the SSU gene followed by sequence data analysis using dada2 is most informative for the taxonomic analysis of individual nematodes. We tried to improve branch structures in the phylogenetic trees using long sequences in place of regional short sequences; however, the effect was not significant. Finally, we succeeded in clarifying the taxonomically biased nematode community in the copse soils: over 50% of nematodes belonging to the order Dorylaimida followed by Rhabditida and Triplonchida. Additionally, approximately 70% of the total isolates were classified as plant feeders and fungal feeders, which suggested a plant and fungi-rich environment in the copse soils. It will be interesting to further investigate which of the four regions of the SSU gene provide the most useful and accurate information via DNA metabarcoding in other biomes, especially in relation to nematodes.

## Supporting information

S1 Table Nematode species used in the phylogenetic analyses and accession numbers. (DOCX)

S2 Table Sequence Variants (SVs) and sample IDs in each Z01rOTU. (DOCX)

S3 Table Taxonomic classifications of regional Z01rOTUs based on the SILVA database. (DOCX)

S4 Table Taxonomic assignments of regional and combined Z01rOTUs using BLASTN search. (DOCX)

S5 Table The largest numbers of reference species in the cluster in the phylogenetic trees. (DOCX)

S1 Fig PCR products amplified from 96 nematode DNAs in four target SSU regions. Five microliter aliquots of each reaction mixture containing nematode DNA with the indicated sample ID number were subjected to 1% agarose gel electrophoresis. The PCR products from the indicated target regions in the gels were visualized using successive ethidium bromide staining. Gene Ladder Wide 1 (Nippon Gene, Toyama, Japan) was used as a size marker.
(PDF)

S2 Fig Abundance and taxonomic assignment of nematode-derived SVs to orders using the dada2 algorithm. The abundance of nematode-derived SVs in each sample ID is shown in the histograms (A, region 1; B, region 2; C, region 3; and D, region 4). The orders of the SVs are also shown by the colors indicated above. Lines in the histogram indicate distinct SVs. NA: Not assigned.
(PDF)

S3 Fig Abundance and taxonomic assignment of nematode-derived SVs to orders using the deblur algorithm. The abundance of nematode-derived SVs in each sample ID is shown in the histograms (A, region 1; B, region 2; C, region 3; and D, region 4). The orders of the SVs were also shown by the colors indicated above. Lines in the histogram indicate distinct SVs. NA: Not assigned.
(PDF)

S4 Fig Phylogenetic tree of full-length SSU sequences from 116 reference nematode species. Nucleotide sequences of the SSU gene from 116 reference nematodes and *Halobiotus crispae* (phylum Tardigrada) were aligned, and the phylogenetic tree was prepared using the BOOTSTRAP N-J TREE algorithm (bootstrap: 1000 replicates) with the ClustalX package. In the resultant cladogram, a colored circle corresponding to the scientific name indicates the species’ order, as shown in the box below. The orders were classified into two classes of phylum Nematoda (Chromadorea and Enoplea), and two subclasses of class Enoplea (Enoplia and Dorylaimia). The species that belong to the order Rhabditida were further classified into three groups (i.e., two suborders Tylenchina and Spirurina, and others) in parentheses. The order Benthimermithida belongs to the phylum Nematoda with no rank. Bootstrap numbers of over 500 per 1000 were indicated at the nodes of the cladogram. The abbreviation “Ref_FL” means the phylogenetic tree built by full-length sequences of nematode reference species.
(PDF)

S5 Fig Phylogenetic trees built by combined two regional sequences of the SSU gene from 116 reference nematode species and Z01rOTUs. Two combined SSU regions are indicated in the upper left. R1_2 means the phylogenetic tree (cladogram) that was built using the combined nucleotide sequences of two SSU regions (regions 1 and 2) from nematode species, and the combined regional Z01rOTU_R1 and _R2 in this order. See the legend in S4 Fig for other descriptions on the phylogenetic tree.
(PDF)

S6 Fig Phylogenetic tree built by combined three regional sequences of the SSU gene from 116 reference nematode species and Z01rOTUs. R2_3_4 means the phylogenetic tree (cladogram) was built using the combined nucleotide sequences of three SSU regions (regions 2, 3 and 4) from nematode species, and the combined regional Z01rOTU_R2, R3 and _R4 in this order. See the legend in S4 Fig for other descriptions on the phylogenetic tree.
(PDF)

S7 Fig Phylogenetic trees built by regional SSU sequences (regions 1 and 2) from reference nematode species alone. The SSU region of reference nematode species used for the preparation of the cladograms is indicated in the upper left. See the legend in S4 Fig for other descriptions on the phylogenetic tree.
(PDF)

S8 Fig Phylogenetic trees built by regional SSU sequences (regions 3 and 4) from reference nematode species alone. See the legend in S7 Fig for descriptions on the phylogenetic tree.
(PDF)

S9 Fig Phylogenetic trees built by combined two regional SSU sequences from reference nematode species alone. Two combined SSU regions used for the preparation of the cladograms are indicated in the upper left. Ref_R1_2 means the phylogenetic tree was built using the combined nucleotide sequences of the SSU regions 1 and 2 from nematode species in this order. See the legend in S4 Fig for other descriptions on the phylogenetic tree.
(PDF)

S10 Fig Phylogenetic trees built by combined three and four regional SSU sequences from reference nematode species alone. Combined SSU regions used for the preparation of the cladograms are indicated in the upper left. Ref_R2_3_4 means the phylogenetic tree built using the combined nucleotide sequences of the SSU regions 2, 3 and 4 from nematode species in this order. See the legend in S4 Fig for other descriptions on the phylogenetic tree.
(PDF)

S1 Raw images Aliquots of each reaction mixture containing nematode DNA with the indicated sample ID number were subjected to 1% agarose gel electrophoresis (eight samples per group, loading samples from left to right to the indicated lanes). The PCR products from the indicated target regions in the gels were visualized using successive ethidium bromide staining. Fluorescent images of agarose gels were acquired using the FAS-III gel imaging system (Nippon Genetics Co., Tokyo, Japan), and original TIFF images shown in each file were used to prepare four regional combined figures of S1 Fig after removing unrelated area. The sample ID in each lane was indicated on top of a gel image and correspond to the sample ID numbers in parentheses in S1 Fig. The amplified region and sample IDs contained in each gel were also indicated at the upper left in each file. M: lane with a size marker (Gene Ladder Wide 1).
(PDF)
