# Supplementary material for: Taxonomic profiling of individual nematodes isolated from copse soils using deep amplicon sequencing of four distinct regions of the 18S ribosomal RNA gene
Source: PLoS One. 2020 Oct 7;15(10):e0240336. doi: 10.1371/journal.pone.0240336 (PMC7540906; doi:10.1371/journal.pone.0240336)
Supplement: S1 Table — (DOCX) [file pone.0240336.s001.docx]

**S1 Table. Nematode species used in the phylogenetic analyses and accession numbers.**

Names of species, order, family, and GenBank accession number of nucleotide sequences of 18S ribosomal small subunit RNA (SSU) genes of 117 reference species are shown and were used for phylogenetic analysis. The SSU reference sequences from 116 nematode species were derived from one species per family; however, 8 of 57 families were selected from the suborder Spirurina. Additionally, the following families were excluded due to either lacking available full-length sequences or unsuccessful clustering: Agfidae and Angiostomatidae (order Rhabditida), Dioctophymatidae (order Dioctophymatida), Muspiceidae and Robertdollfusidae (order Muspiceida), Rhaptothyreidae (order Rhaptothyreida), Rhabdodemaniidae (order Triplonchida), and Phanodermatidae, Oncholaimidae, and Thoracostomopsidae (order Enoplida). *Halobiotus crispae* (phylum Tardigrada) was used as the outgroup. Colors represent the orders or suborders of the reference species and are used to indicate (sub-)order-levels of taxonomic ranks in the resultant phylogenetic trees.

| Species | Order | Family | Accession no. | Color |
| --- | --- | --- | --- | --- |
| *Achromadora cf terricola* | Chromadorida | Cyatholaimidae | AY593940 |  |
| *Acrostichus floridensis* | Rhabditida (Rhabditina) | Diplogasteridae | LC210624 |  |
| *Alaimus parvus* | Enoplida | Alaimidae | AY284738 |  |
| *Alinema amazonicum* | Rhabditida (Spirurina) | Philometridae | DQ442672 |  |
| *Anatonchus tridentatus* | Mononchida | Anatonchidae | AJ966474 |  |
| *Anoplostoma rectospiculum* | Enoplida | Anoplostomatidae | AY590149 |  |
| *Anticoma* sp. | Enoplida | Anticomidae | MN250100 |  |
| *Aphanolaimus aquaticus* | Plectida | Aphanolaimidae | AY593932 |  |
| *Aphelenchoides blastophtorus* | Rhabditida (Tylenchina) | Aphelenchoididae | AY284644 |  |
| *Aphelenchus* sp. | Rhabditida (Tylenchina) | Aphelenchidae | KU180667 |  |
| *Aporcelaimellus obtusicaudatus* | Dorylaimida (Dorylaimina) | Aporcelaimidae | AY284811 |  |
| *Ascaris suum* | Rhabditida (Spirurina) | Ascarididae | U94367 |  |
| *Astomonema* sp. | Monhysterida | Siphonolaimidae | DQ408761 |  |
| *Aulolaimus oxycephalus* | Plectida | Aulolaimidae | KJ636344 |  |
| *Axonolaimus* sp. | Araeolaimida | Axonolaimidae | EF591331 |  |
| *Baruscapillaria obsignata* | Trichinellida | Capillariidae | LC425003 |  |
| *Bathylaimus assimilis* | Enoplida | Tripyloididae | AJ966476 |  |
| *Bathyodontus cylindricus* | Mononchida | Bathyodontidae | AY552964 |  |
| *Brevibucca* sp. | Rhabditida | Brevibuccidae | AF202163 |  |
| *Brugia malayi* | Rhabditida (Spirurina) | Onchocercidae | AF036588 |  |
| *Bunonema* sp. | Rhabditida (Rhabditina) | Bunonematidae | U81582 |  |
| *Caenorhabditis elegans* | Rhabditida (Rhabditina) | Rhabditidae | X03680 |  |
| *Calyptronema* sp. | Enoplida | Enchelidiidae | FJ040503 |  |
| *Campydora demonstrans* | Enoplida | Campydoridae | FJ969118 |  |
| *Cephalobus oryzae* | Rhabditida (Tylenchina) | Cephalobidae | AF034390 |  |
| *Ceramonema altogolfi* | Plectida | Ceramonematidae | JN815320 |  |
| *Choanolaimus psammophilus* | Chromadorida | Choanolaimidae | AY284716 |  |
| *Chromadorina bioculata* | Chromadorida | Chromadoridae | KJ636221 |  |
| *Clavicaudoides trophurus* | Dorylaimida (Nygolaimina) | Nygolaimidae | AY284772 |  |
| *Coslenchus franklinae* | Rhabditida (Tylenchina) | Tylenchidae | AY284583 |  |
| *Cosmocercoides tonkinensis* | Rhabditida (Spirurina) | Cosmocercidae | AB908160 |  |
| *Crenosoma mephitidis* | Strongylida | Crenosomatidae | AY295805 |  |
| *Cryptonchus tristis* | Mononchida | Cryptonchidae | EF207244 |  |
| *Cylindrolaimus communis* | Araeolaimida | Diplopeltidae | AY593939 |  |
| *Daptonema procerus* | Monhysterida | Xyalidae | AF047889 |  |
| *Daubaylia potomaca* | Rhabditida (Rhabditina) | Daubayliidae | KU180669 |  |
| *Dentostomella* sp. | Rhabditida (Spirurina) | Heteroxynematidae | AF036590 |  |
| *Deontolaimus papillatus* | Plectida | Leptolaimidae | EF591322 |  |
| *Desmolaimus* sp. | Monhysterida | Linhomoeidae | EF591332 |  |
| *Desmoscolex* sp. | Desmoscolecida | Desmoscolecidae | MN250116 |  |
| *Dintheria tenuissima* | Triplonchida | Bastianiidae | KJ636403 |  |
| *Diphterophora communis* | Dorylaimida (Dorylaimina) | Diphterophoridae | AY593955 |  |
| *Diphtherophora obesus* | Triplonchida | Diphtherophoridae | AY552968 |  |
| *Diplogasteroides halleri* | Rhabditida (Rhabditina) | Diplogasteroididae | KJ877227 |  |
| *Diplolaimelloides meyli* | Monhysterida | Monhysteridae | AF036644 |  |
| *Diplopeltoides* sp. | Plectida | Diplopeltoididae | MN250115 |  |
| *Discolaimus major* | Dorylaimida (Dorylaimina) | Qudsianematidae | AY284828 |  |
| *Ditylenchus brevicauda* | Rhabditida (Tylenchina) | Anguinidae | AY284635 |  |
| *Domorganus macronephriticus* | Plectida | Ohridiidae | FJ969122 |  |
| *Dorylaimoides limnophilus* | Dorylaimida (Dorylaimina) | Mydonomidae | AY593950 |  |
| *Dorylaimus stagnalis* | Dorylaimida (Dorylaimina) | Dorylaimidae | AY284776 |  |
| *Enchodelus* sp. | Dorylaimida (Dorylaimina) | Nordiidae | AY284792 |  |
| *Enoplus brevis* | Enoplida | Enoplidae | U88336 |  |
| *Epsilonema* sp. | Desmodorida | Epsilonematidae | FJ182218 |  |
| *Eubostrichus parasitiferus* | Desmodorida | Desmodoridae | Y16916 |  |
| *Fescia grossa* | Rhabditida | Chambersiellidae | KU180670 |  |
| *Filaroides martis* | Strongylida | Filaroididae | AY295807 |  |
| *Globodera pallida* | Rhabditida (Tylenchina) | Heteroderidae | AF036592 |  |
| *Gnathostoma binucleatum* | Rhabditida (Spirurina) | Gnathostomatidae | Z96946 |  |
| *Haemonchus similis* | Strongylida | Haemonchidae | L04152 |  |
| *Halichoanolaimus* sp. | Chromadorida | Selachinematidae | MN250043 |  |
| *Haliplectus cf. dorsalis* | Plectida | Haliplectidae | FJ969123 |  |
| *Helicotylenchus certus* | Rhabditida (Tylenchina) | Hoplolaimidae | KJ869372 |  |
| *Heterorhabditis bacteriophora* | Strongylida | Heterorhabditidae | AF036593 |  |
| *Hysterothylacium fortalezae* | Rhabditida (Spirurina) | Raphidascarididae | U94374 |  |
| *Ironus dentifurcatus* | Enoplida | Ironidae | AJ966487 |  |
| *Isolaimium* sp. | Isolaimida | Isolaimiidae | AY552971 |  |
| *Leptonchus granulosus* | Dorylaimida (Dorylaimina) | Leptonchidae | AY284831 |  |
| *Longidorus elongatus* | Dorylaimida (Dorylaimina) | Longidoridae | AF036594 |  |
| *Loofia thienemanni* | Rhabditida (Tylenchina) | Hemicycliophoridae | AY284629 |  |
| *Meloidogyne incognita* | Rhabditida (Tylenchina) | Meloidogynidae | AY284621 |  |
| *Mermis nigrescens* | Mermithida | Mermithidae | AF036641 |  |
| *Mesocriconema xenoplax* | Rhabditida (Tylenchina) | Criconematidae | AY284625 |  |
| *Mononchus aquaticus* | Mononchida | Mononchidae | AY284764 |  |
| *Monoposthia* sp. | Desmodorida | Monoposthiidae | FJ040505 |  |
| *Myctolaimus* sp. | Rhabditida (Rhabditina) | Cylindrocorporidae | AB478638 |  |
| *Mylonchulus sigmaturus* | Mononchida | Mylonchulidae | AY284755 |  |
| *Myolaimus byersi* | Rhabditida | Myolaimidae | KU180665 |  |
| *Odontolaimus* sp. | Triplonchida | Odontolaimidae | FJ969131 |  |
| *Odontopharynx longicaudata* | Rhabditida (Rhabditina) | Odontopharyngidae | FJ040449 |  |
| *Oxydirus oxycephalus* | Dorylaimida (Dorylaimina) | Belondiridae | AY284824 |  |
| *Panagrellus redivivus* | Rhabditida (Tylenchina) | Panagrolaimidae | AF083007 |  |
| *Paractinolaimus macrolaimus* | Dorylaimida (Dorylaimina) | Actinolaimidae | AY284826 |  |
| *Paradraconema jejuense* | Desmodorida | Draconematidae | FJ182220 |  |
| *Paratrichodorus anemones* | Triplonchida | Trichodoridae | KJ636333 |  |
| *Paratylenchus straeleni* | Rhabditida (Tylenchina) | Paratylenchidae | AY284630 |  |
| *Plectus aquatilis* | Plectida | Plectidae | AF036602 |  |
| *Pratylenchoides ritteri* | Rhabditida (Tylenchina) | Merliniidae | AJ966497 |  |
| *Pratylenchus crenatus* | Rhabditida (Tylenchina) | Pratylenchidae | AY284610 |  |
| *Prismatolaimus intermedius* | Triplonchida | Prismatolaimidae | AY284729 |  |
| *Pristionchus lheritieri* | Rhabditida (Rhabditina) | Neodiplogasteridae | AF036640 |  |
| *Prodesmodora* sp. | Desmodorida | Microlaimidae | FJ040477 |  |
| *Proplatycoma* sp. | Enoplida | Leptosomatidae | LC495490 |  |
| *Rhabdolaimus aquaticus* | Enoplida | Rhabdolaimidae | FJ969139 |  |
| *Rhigonema thysanophora* | Rhabditida (Spirurina) | Rhigonematidae | EF180067 |  |
| *Rhyssocolpus paradoxus* | Dorylaimida (Dorylaimina) | Pararhyssocolpidae | KM092521 |  |
| *Sabatieria pulchra* | Araeolaimida | Comesomatidae | EF591335 |  |
| *Sauertylenchus maximus* | Rhabditida (Tylenchina) | Telotylenchidae | AY284602 |  |
| *Setostephanolaimus spartinae* | Plectida | Camacolaimidae | EF591321 |  |
| *Soboliphyme baturini* | Dioctophymatida | Soboliphymatidae | AY277895 |  |
| *Steinernema carpocapsae* | Rhabditida (Tylenchina) | Steinernematidae | AF036604 |  |
| *Subsphaerolaimus* sp. | Monhysterida | Sphaerolaimidae | MN250098 |  |
| *Tarvaia* sp. | Plectida | Tarvaiidae | JN815318 |  |
| *Teratocephalus lirellus* | Rhabditida | Teratocephalidae | AF036607 |  |
| *Thalassoalaimus pirum* | Enoplida | Oxystominidae | FJ040500 |  |
| *Tobrilus cf. gracilis* | Triplonchida | Tobrilidae | KJ636228 |  |
| *Trefusia* sp. | Enoplida | Trefusiidae | HM564576 |  |
| *Trichinella spiralis* | Trichinellida | Trichinellidae | TSU60231 |  |
| *Trichosomoides crassicauda* | Trichinellida | Trichosomoididae | LC425007 |  |
| *Trichuris leporis* | Trichinellida | Trichuridae | HF586913 |  |
| *Tripyla cf. filicaudata* | Triplonchida | Tripylidae | AY284730 |  |
| *Tripylina arenicola* | Enoplida | Trischistomatidae | KJ636243 |  |
| *Trophomera* sp. | Benthimermithida | Benthimermithidae | JN625216 |  |
| *Tylencholaimellus affinis* | Dorylaimida (Dorylaimina) | Tylencholaimellidae | AY552978 |  |
| *Tylencholaimus* sp. | Dorylaimida (Dorylaimina) | Tylencholaimidae | AY284834 |  |
| *Tylopharynx foetidus* | Rhabditida (Rhabditina) | Tylopharyngidae | EU306343 |  |
| *Halobiotus crispae* | Tardigrada (phylum) | Hypsibiidae | EF620401 |  |
