# Supplementary material for: Taxonomic profiling of individual nematodes isolated from copse soils using deep amplicon sequencing of four distinct regions of the 18S ribosomal RNA gene
Source: PLoS One. 2020 Oct 7;15(10):e0240336. doi: 10.1371/journal.pone.0240336 (PMC7540906; doi:10.1371/journal.pone.0240336)
Supplement: S2 Table — (DOCX) [file pone.0240336.s002.docx]

**S2 Table. Sequence variants (SVs) and sample IDs in each Z01rOTU.**

The nematode isolates (sample IDs) with identical regional SVs were collected to generate rOTUs. The resultant rOTUs were named as Z01rOTU plus a number in the order of the numbers of isolates (e.g., Z01rOTU01). Major SV and minor SV in each rOTU indicate the most abundant SVs and the second most abundant SVs, respectively. The sample IDs indicated by bold italics contain two taxonomically different nematodes, and the SVs derived from contaminated or predated nematodes were indicated in red. ND: No sequence data.

| rOTU name | Sample ID | Region 1 | | Region 2 | | Region 3 | | Region 4 | |
| --- | --- | --- | --- | --- | --- | --- | --- | --- | --- |
|  |  | Major SV | Minor SV | Major SV | Minor SV | Major SV | Minor SV | Major SV | Minor SV |
| Z01rOTU01 | 02 | SV_2 |  | SV_2 |  | SV_4 | SV_5 | SV_1 |  |
|  | 04 | SV_2 |  | SV_2 |  | SV_4 | SV_5 | SV_1 |  |
|  | 06 | SV_2 | SV_13 | SV_2 |  | SV_4 |  | SV_1 |  |
|  | 07 | SV_2 |  | SV_2 |  | SV_4 | SV_5 | SV_1 |  |
|  | 15 | SV_2 |  | SV_2 |  | SV_4 | SV_5 | SV_1 |  |
|  | 28 | SV_2 |  | SV_2 |  | SV_4 | SV_5 | SV_1 |  |
|  | 35 | SV_2 | SV_13 | SV_2 |  | SV_4 |  | SV_1 |  |
|  | 40 | SV_2 | SV_13 | SV_2 |  | SV_4 |  | SV_1 |  |
|  | 44 | SV_2 |  | SV_2 |  | SV_4 | SV_5 | SV_1 |  |
|  | 59 | SV_2 |  | SV_2 |  | SV_4 | SV_5 | SV_1 |  |
|  | 61 | SV_2 |  | SV_2 |  | SV_4 | SV_5 | SV_1 |  |
|  | 75 | SV_2 |  | SV_2 |  | SV_4 | SV_5 | SV_1 |  |
|  | 77 | SV_2 | SV_13 | SV_2 |  | SV_4 |  | SV_1 |  |
| Z01rOTU02 | 09 | SV_3 | SV_12 | ND |  | SV_3 |  | SV_3 |  |
|  | 10 | SV_3 | SV_12 | SV_3 |  | SV_3 |  | SV_3 |  |
|  | 19 | SV_3 | SV_12 | SV_3 |  | SV_3 |  | SV_3 |  |
|  | 27 | SV_3 | SV_12 | SV_3 |  | SV_3 |  | SV_3 |  |
|  | 43 | SV_3 | SV_12 | SV_3 |  | SV_3 |  | SV_3 |  |
|  | 47 | SV_3 | SV_12 | SV_3 |  | SV_3 |  | SV_3 |  |
|  | 50 | SV_3 | SV_12 | SV_3 |  | SV_3 |  | SV_3 |  |
|  | 57 | SV_3 | SV_12 | SV_3 |  | SV_3 |  | SV_3 |  |
|  | 69 | SV_3 | SV_12 | SV_3 |  | SV_3 |  | SV_3 |  |
|  | 71 | SV_3 | SV_12 | SV_3 |  | SV_3 |  | SV_3 |  |
|  | 84 | SV_3 | SV_12 | SV_3 |  | SV_3 |  | SV_3 |  |
| Z01rOTU03 | 03 | SV_1 |  | SV_1 |  | SV_1 |  | SV_2 |  |
|  | 34 | SV_1 |  | SV_1 |  | SV_1 |  | SV_2 |  |
|  | 42 | SV_1 |  | SV_1 |  | SV_1 |  | SV_2 |  |
|  | 45 | SV_1 |  | SV_1 |  | SV_1 |  | SV_2 |  |
|  | 67 | SV_1 |  | SV_1 |  | SV_1 |  | SV_2 |  |
|  | 73 | SV_1 |  | SV_1 |  | SV_1 |  | SV_2 |  |
|  | 82 | SV_1 |  | SV_1 |  | SV_1 |  | SV_2 |  |
|  | 83 | SV_1 |  | SV_1 |  | SV_1 |  | SV_2 |  |
|  | 85 | SV_1 |  | SV_1 |  | SV_1 |  | SV_2 |  |
|  | 87 | SV_1 |  | SV_1 |  | SV_1 |  | SV_2 |  |
|  | 93 | SV_1 |  | SV_1 |  | SV_1 |  | SV_2 |  |
| Z01rOTU04 | 36 | SV_4 |  | SV_6 | SV_11 | SV_2 |  | SV_6 | SV_16 |
|  | 37 | SV_4 |  | SV_6 | SV_11 | SV_2 |  | SV_6 | SV_16 |
|  | ***46*** | ND |  | ND |  | SV_2 | SV_7 | ND |  |
|  | 53 | SV_4 |  | SV_6 | SV_11 | SV_2 |  | SV_6 | SV_16 |
|  | 63 | SV_4 |  | SV_6 | SV_11 | SV_2 |  | SV_6 | SV_16 |
|  | ***74*** | SV_4 | SV_10 | SV_6 | SV_11/SV_7 | SV_2 | SV_7 | SV_6 | SV_16 |
|  | ***78*** | SV_4 | SV_8 | SV_8 | SV_11/SV_6 | SV_2 |  | SV_13 | SV_16/SV_6 |
| Z01rOTU05 | 30 | SV_6 |  | SV_5 |  | SV_6 | SV_18 | SV_5 |  |
|  | 33 | SV_6 |  | SV_5 |  | SV_6 | SV_18 | SV_5 |  |
|  | 70 | SV_6 |  | SV_5 |  | SV_6 | SV_18 | SV_5 |  |
|  | 80 | SV_6 |  | SV_5 |  | SV_6 | SV_18 | SV_5 |  |
| Z01rOTU06 | 89 | SV_10 | SV_31 | SV_7 | SV_22 | SV_7 |  | SV_8 | SV_31 |
|  | 94 | SV_10 | SV_31 | SV_7 | SV_22 | SV_7 |  | SV_8 | SV_31 |
|  | 96 | SV_10 |  | SV_7 | SV_22 | SV_7 | SV_23 | SV_8 | SV_31 |
| Z01rOTU07 | 72 | ND |  | ND |  | SV_8 |  | ND |  |
|  | 91 | SV_11 |  | SV_10 |  | SV_8 |  | SV_10 |  |
|  | 95 | SV_11 |  | SV_10 |  | SV_8 |  | SV_10 |  |
| Z01rOTU08 | ***41*** | SV_7 | SV_10 | SV_9 | SV_7 | ND |  | SV_12 | SV_8 |
|  | 51 | SV_7 |  | SV_9 | SV_23 | SV_2 |  | SV_12 |  |
| Z01rOTU09 | ***31*** | SV_8 | SV_11 | SV_8 | SV_10 | SV_8 |  | SV_13 | SV_10 |
|  | 76 | SV_8 |  | SV_8 |  | ND |  | SV_13 | SV_35 |
| Z01rOTU10 | 20 | SV_9 |  | ND |  | SV_9 |  | SV_15 | SV_64 |
|  | 88 | SV_9 |  | SV_27 |  | SV_9 |  | SV_15 |  |
| Z01rOTU11 | 16 | ND |  | SV_13 |  | SV_11 |  | SV_23 |  |
|  | 22 | SV_23 |  | SV_13 |  | SV_11 |  | SV_23 |  |
| Z01rOTU12 | ***18*** | ND |  | ND |  | SV_14 | SV_4 | ND |  |
|  | 24 | SV_25 |  | SV_18 |  | SV_14 |  | SV_19 |  |
| Z01rOTU13 | 49 | SV_15 |  | SV_15 |  | SV_2 |  | SV_21 |  |
| Z01rOTU14 | 68 | SV_16 |  | SV_14 |  | SV_13 |  | SV_18 |  |
| Z01rOTU15 | 52 | SV_18 |  | SV_16 |  | SV_12 |  | SV_22 |  |
| Z01rOTU16 | 01 | SV_19 |  | SV_17 |  | SV_16 |  | SV_24 |  |
| Z01rOTU17 | 56 | SV_20 |  | SV_19 |  | SV_15 |  | SV_25 |  |
| Z01rOTU18 | ***25*** | SV_27 | SV_3 | SV_20 | SV_3 | SV_17 | SV_3 | SV_29 | SV_3 |
