# Supplementary material for: Taxonomic profiling of individual nematodes isolated from copse soils using deep amplicon sequencing of four distinct regions of the 18S ribosomal RNA gene
Source: PLoS One. 2020 Oct 7;15(10):e0240336. doi: 10.1371/journal.pone.0240336 (PMC7540906; doi:10.1371/journal.pone.0240336)
Supplement: S3 Table — (DOCX) [file pone.0240336.s003.docx]

**S3 Table. Taxonomic classifications of regional Z01rOTUs based on the SILVA database.**

Orders of the four regional sequences of each Z01rOTU were based on the ranks D8 and D9 of the SILVA database. Any taxonomic information of lower rank than “order” was indicated in the columns of “Taxonomic data from ranks D10 and D11”. The suborder Tylenchida appeared in rank D8 and was translated to the order Rhabditida. ND: No sequence data. NA: Not assigned.

|  | **Order from ranks D8 and D9 in SILVA database** | | | |
| --- | --- | --- | --- | --- |
| rOTU name | Region 1 | Region 2 | Region 3 | Region 4 |
| Z01rOTU01 | Rhabditida | Rhabditida | Rhabditida | Rhabditida |
| Z01rOTU02 | Dorylaimida | Dorylaimida | Dorylaimida | Dorylaimida |
| Z01rOTU03 | Dorylaimida | Dorylaimida | Dorylaimida | Dorylaimida |
| Z01rOTU04 | Dorylaimida | Dorylaimida | Dorylaimida | Dorylaimida |
| Z01rOTU05 | Triplonchida | Triplonchida | Triplonchida | Triplonchida |
| Z01rOTU06 | Rhabditida | Rhabditida | Rhabditida | Rhabditida |
| Z01rOTU07 | Dorylaimida | Dorylaimida | Dorylaimida | Dorylaimida |
| Z01rOTU08 | Dorylaimida | Dorylaimida | Dorylaimida | Dorylaimida |
| Z01rOTU09 | Triplonchida | Triplonchida | ND | Triplonchida |
| Z01rOTU10 | Rhabditida | Rhabditida | Rhabditida | Rhabditida |
| Z01rOTU11 | Rhabditida | Rhabditida | Rhabditida | Rhabditida |
| Z01rOTU12 | Triplonchida | Triplonchida | Triplonchida | Triplonchida |
| Z01rOTU13 | Dorylaimida | Dorylaimida | Dorylaimida | Dorylaimida |
| Z01rOTU14 | Triplonchida | Triplonchida | Triplonchida | Triplonchida |
| Z01rOTU15 | Araeolaimida | Araeolaimida | Araeolaimida | Araeolaimida |
| Z01rOTU16 | Triplonchida | Triplonchida | Triplonchida | NA |
| Z01rOTU17 | Triplonchida | Triplonchida | Triplonchida | Triplonchida |
| Z01rOTU18 | Triplonchida | Triplonchida | Triplonchida | Triplonchida |
|  | **Taxonomic data from ranks D10 and D11** | | | |
| rOTU name | Region 1 | Region 2 | Region 3 | Region 4 |
| Z01rOTU01 | NA | Mesocriconema xenoplax | NA | Mesocriconema xenoplax |
| Z01rOTU02 | Ambiguous_taxa | Ambiguous_taxa | Ambiguous_taxa | NA |
| Z01rOTU03 | NA | NA | Ambiguous_taxa | NA |
| Z01rOTU04 | Ambiguous_taxa | Nematoda environmental sample | Ambiguous_taxa | NA |
| Z01rOTU05 | Paratrichodorus allius | Paratrichodorus porosus | Paratrichodorus porosus | Paratrichodorus porosus |
| Z01rOTU06 | NA | Boleodorus thylactus | Boleodorus thylactus | Boleodorus thylactus |
| Z01rOTU07 | NA | Tylencholaimus mirabilis | NA | Proleptonchus weischeri |
| Z01rOTU08 | Ambiguous_taxa | NA | Ambiguous_taxa | NA |
| Z01rOTU09 | Diphtherophora obesus | NA | ND | NA |
| Z01rOTU10 | NA | Acrobeles ciliatus | NA | NA |
| Z01rOTU11 | NA | NA | NA | Basiria duplexa |
| Z01rOTU12 | Phaseoleae environmental sample | Schizomidae environmental sample | Schizomidae environmental sample | Ambiguous_taxa |
| Z01rOTU13 | Ambiguous_taxa | Nematoda environmental sample | Ambiguous_taxa | Ambiguous_taxa |
| Z01rOTU14 | NA | NA | Nematoda environmental sample | NA |
| Z01rOTU15 | NA | Plectus sp. | Ambiguous_taxa | Acrobeloides buetschlii |
| Z01rOTU16 | Diphtherophora obesus | NA | NA | NA |
| Z01rOTU17 | Ambiguous_taxa | Ambiguous_taxa | Ambiguous_taxa | Ambiguous_taxa |
| Z01rOTU18 | NA | NA | NA | Odontolaimus sp. OdLaSp1 |
