# Supplementary material for: Taxonomic profiling of individual nematodes isolated from copse soils using deep amplicon sequencing of four distinct regions of the 18S ribosomal RNA gene
Source: PLoS One. 2020 Oct 7;15(10):e0240336. doi: 10.1371/journal.pone.0240336 (PMC7540906; doi:10.1371/journal.pone.0240336)
Supplement: S4 Table — (DOCX) [file pone.0240336.s004.docx]

**S4 Table. Taxonomic assignments of regional and combined Z01rOTUs using BLASTN search.**

The orders, families, and genera of the closest species hit using the BLASTN search are shown. The BLASTN search was performed by querying the four regional and combined nucleotide sequences of the Z01rOTUs in the NCBI site in April 2020. Taxonomic information of the scientific name-known species with the lowest e-value and 100% query cover was used for the taxonomic assignment of the four regional Z01rOTUs. An artificial nucleotide sequence combined with four regional sequences (R1_2_3_4) was also queried to detect homologous nematode sequences by BLASTN search in July 2020. Because there were several sequences with e-values of zero, we used the taxonomic information of species with the following values of percent identity and query cover in each Z01rOTU: 100%/100% for Z01rOTU01, 05, and 10; 100%/99% for Z01rOTU15; 100%/45% for Z01rOTU08; 99%/100% for Z01rOTU04, 12 and 17; 99.3%/100% for Z01rOTU13; 99%/95% for Z01rOTU11; 98%/100% for Z01rOTU02, 03, 06 and 07; 94%/99% for Z01rOTU14 and 18; and 91%/44% for Z01eOTU16. ND: No sequence data.

|  | **Order** | | | | |
| --- | --- | --- | --- | --- | --- |
| rOTU name | Region 1 | Region 2 | Region 3 | Region 4 | R_1_2_3_4 |
| Z01rOTU01 | Rhabditida | Rhabditida | Rhabditida | Rhabditida | Rhabditida |
| Z01rOTU02 | Dorylaimida | Dorylaimida | Dorylaimida | Dorylaimida | Dorylaimida |
| Z01rOTU03 | Dorylaimida | Dorylaimida | Dorylaimida | Dorylaimida | Dorylaimida |
| Z01rOTU04 | Dorylaimida | Dorylaimida | Dorylaimida | Dorylaimida | Dorylaimida |
| Z01rOTU05 | Triplonchida | Triplonchida | Triplonchida | Triplonchida | Triplonchida |
| Z01rOTU06 | Rhabditida | Rhabditida | Rhabditida | Rhabditida | Rhabditida |
| Z01rOTU07 | Dorylaimida | Dorylaimida | Dorylaimida | Dorylaimida | Dorylaimida |
| Z01rOTU08 | Dorylaimida | Dorylaimida | Dorylaimida | Dorylaimida | Dorylaimida |
| Z01rOTU09 | Dorylaimida | Triplonchida | ND | Triplonchida | ND |
| Z01rOTU10 | Rhabditida | Rhabditida | Rhabditida | Rhabditida | Rhabditida |
| Z01rOTU11 | Rhabditida | Rhabditida | Rhabditida | Rhabditida | Rhabditida |
| Z01rOTU12 | Triplonchida | Triplonchida | Triplonchida | Triplonchida | Triplonchida |
| Z01rOTU13 | Dorylaimida | Dorylaimida | Dorylaimida | Dorylaimida | Dorylaimida |
| Z01rOTU14 | Dorylaimida | Triplonchida | Triplonchida | Triplonchida | Triplonchida  Dorylaimida  Rhabditida |
| Z01rOTU15 | Plectida  Dorylamida | Plectida | Plectida | Plectida  Dorylamida Rhabditida | Plectida  Dorylaimia |
| Z01rOTU16 | Dorylaimida | Triplonchida | Triplonchida | Triplonchida | Triplonchida |
| Z01rOTU17 | Triplonchida | Triplonchida | Triplonchida | Triplonchida | Triplonchida |
| Z01rOTU18 | Triplonchida  Dorylaimida | Triplonchida | Triplonchida | Triplonchida | Triplonchida |
|  | **Family** | | | | |
| Z01rOTU01 | Criconematidae | Criconematidae | Criconematidae | Criconematidae | Criconematidae |
| Z01rOTU02 | Belondiridae | Actinolaimidae | Tylencholaimellidae  Aporcelaimida  Mydonomidae | Tylencholaimellidae | Tylencholaimellidae  Belondiridae |
| Z01rOTU03 | Qudsianematidae  Dorylaimidae  Nordiidae | Tylencholaimidae | Longidoridae | Tylencholaimidae | Aporcelaimidae  Dorylaimoidea  Leptonchidae  Tylencholaimidae |
| Z01rOTU04 | Aporcelaimidae | Mydonomidae | Dorylaimidae  Aporcelaimidae  Qudsianematidae | Mydonomidae | Mydonomidae  Dorylaimidae  Pararhyssocolpidae  Qudsianematidae  Belondiridae |
| Z01rOTU05 | Trichodoridae | Trichodoridae | Trichodoridae | Trichodoridae | Trichodoridae |
| Z01rOTU06 | Tylenchidae | Tylenchidae | Tylenchidae | Tylenchidae | Tylenchidae |
| Z01rOTU07 | Belondiridae | Belondiridae | Belondiridae | Belondiridae | Belondiridae  Leptonchidae |
| Z01rOTU08 | Dorylaimidae | Tylencholaimidae | Dorylaimidae  Qudsianematidae  Aporcelaimidae | Dorylaimidae | Dorylaimidae |
| Z01rOTU09 | Diphterophoridae | Diphtherophoridae | ND | Diphtherophoridae | ND |
| Z01rOTU10 | Cephalobidae | Cephalobidae | Cephalobidae | Cephalobidae | Cephalobidae |
| Z01rOTU11 | Tylenchidae | Tylenchidae | Tylenchidae | Tylenchidae | Tylenchidae |
| Z01rOTU12 | Prismatolaimidae | Prismatolaimidae | Prismatolaimidae | Prismatolaimidae | Prismatolaimidae |
| Z01rOTU13 | Mydonomidae Aporcelaimidae | Mydonomidae | Dorylaimidae  Aporcelaimidae  Qudsianematidae | Mydonomidae  Dorylaimidae | Mydonomidae  Dorylaimidae  Qudsianematidae  Belondiridae |
| Z01rOTU14 | Diphterophoridae | Diphtherophoridae | Diphtherophoridae | Diphtherophoridae  Diphterophoridae | Diphtherophoridae  Diphterophoridae Odontolaimidae  Trichodoridae |
| Z01rOTU15 | Plectidae  Dorylaimidae | Plectidae | Plectidae | Plectidae  Dorylaimidae Cephalobidae | Plectidae  Dorylaimidae |
| Z01rOTU16 | Diphterophoridae | Diphtherophoridae | Trichodoridae | Diphtherophoridae | Diphtherophoridae  Trichodoridae |
| Z01rOTU17 | Prismatolaimidae | Prismatolaimidae | Prismatolaimidae | Prismatolaimidae | Prismatolaimidae |
| Z01rOTU18 | Odontolaimidae | Diphtherophoridae | Odontolaimidae | Diphtherophoridae | Diphtherophoridae Odontolaimidae |
|  | **Genus** | | | | |
| Z01rOTU01 | Mesocriconema | Mesocriconema | Mesocriconema | Mesocriconema | Mesocriconema |
| Z01rOTU02 | Dorylaimellus  Axonchium | Paractinolaimus | Tylencholaimellus  Dorylaimoides Paraxonchium | Tylencholaimellus | Tylencholaimellus  Belondira |
| Z01rOTU03 | Mesodorylaimus  Microdorylaimus  Longidorella | Tylencholaimus | Paralongidorus  Longidorus | Tylencholaimus | Aporcella  Mesodorylaimus  Proleptonchus  Tylencholaimus |
| Z01rOTU04 | Aporcelaimellus | Dorylaimoides | Aporcelaimellus Mesodorylaimus Ecumenicus | Dorylaimoides | Dorylaimoides  Mesodorylaimus  Opisthodorylaimus  Amblydorylaimus  Oxydirus  Pararhyssocolpus |
| Z01rOTU05 | Paratrichodorus | Paratrichodorus | Paratrichodorus | Paratrichodorus | Paratrichodorus |
| Z01rOTU06 | Boleodorus | Boleodorus | Boleodorus | Boleodorus | Boleodorus |
| Z01rOTU07 | Dorylaimellus | Dorylaimellus | Dorylaimellus | Dorylaimellus | Dorylaimellus  Proleptonchus |
| Z01rOTU08 | Opisthodorylaimus | Tylencholaimus | Mesodorylaimus  Aporcelaimellus  Ecumenicus | Mesodorylaimus | Mesodorylaimus |
| Z01rOTU09 | Diphterophora | Diphtherophora | ND | Diphtherophora | ND |
| Z01rOTU10 | Acrobeloides | Acrobeloides | Acrobeloides  Cephalobus | Acrobeloides | Acrobeloides |
| Z01rOTU11 | Basiria | Basiria | Discopersicus | Basiria Discopersicus | Basiria Discopersicus |
| Z01rOTU12 | Prismatolaimus | Prismatolaimus | Prismatolaimus | Prismatolaimus | Prismatolaimus |
| Z01rOTU13 | Dorylaimoides Aporcelaimellus | Dorylaimoides | Mesodorylaimus  Aporcelaimellus  Ecumenicus | Dorylaimoides  Mesodorylaimus | Dorylaimoides  Mesodorylaimus  Opisthodorylaimus  Amblydorylaimus  Oxydirus |
| Z01rOTU14 | Diphterophora | Diphtherophora | Diphtherophora | Diphtherophora  Diphterophora | Diphtherophora  Diphterophora  Odontolaimus  Trichodorus |
| Z01rOTU15 | Plectus  Wilsonema | Ceratoplectus | Plectus | Plectus  Ceratoplectus  Wilsonema  Acrobeloides | Plectus  Ceratoplectus  Wilsonema |
| Z01rOTU16 | Diphterophora | Diphtherophora | Paratrichodorus | Diphtherophora | Diphtherophora  Paratrichodorus |
| Z01rOTU17 | Prismatolaimus | Prismatolaimus | Prismatolaimus | Prismatolaimus | Prismatolaimus |
| Z01rOTU18 | Odontolaimus | Diphtherophora | Odontolaimus | Diphtherophora | Diphtherophora  Odontolaimus |
