# Supplementary material for: Taxonomic profiling of individual nematodes isolated from copse soils using deep amplicon sequencing of four distinct regions of the 18S ribosomal RNA gene
Source: PLoS One. 2020 Oct 7;15(10):e0240336. doi: 10.1371/journal.pone.0240336 (PMC7540906; doi:10.1371/journal.pone.0240336)
Supplement: S5 Table — (DOCX) [file pone.0240336.s005.docx]

**S5 Table. The largest numbers of reference species in the cluster in the phylogenetic trees.**

The largest numbers of reference species in clusters of the phylogenetic trees built by reference species and regional and combined Z01rOTUs (A), and reference species alone (B) are shown for each order. Total no. indicates the total number of species belonging to each order. *Diphterophora communisis* was counted as Triplonchida, as described in the text. Orders containing only a single species were omitted from the table.

| **A. Trees built by regional and combined sequences from reference species and Z01rOTUs** | | | | | | | | | | |  |  |
| --- | --- | --- | --- | --- | --- | --- | --- | --- | --- | --- | --- | --- |
| Order | Total no. | R1 | R2 | R3 | R4 | R1_2 | R3_4 | R2_3_4 | R1_2_3_4 | Reference (Full length) |  |  |
| Araeolaimida | 3 | 2 | 2 | 1 | 3 | 2 | 2 | 2 | 2 | 2 |  |  |
| Chromadorida | 4 | 2 | 3 | 3 | 2 | 2 | 3 | 2 | 2 | 2 |  |  |
| Desmodorida | 5 | 3 | 3 | 3 | 3 | 3 | 3 | 3 | 3 | 3 |  |  |
| Dorylaimida | 13 | 13 | 13 | 13 | 13 | 13 | 13 | 13 | 13 | 13 |  |  |
| Enoplida | 13 | 4 | 12 | 7 | 13 | 7 | 12 | 11 | 13 | 13 |  |  |
| Monhysterida | 5 | 3 | 5 | 3 | 3 | 3 | 2 | 5 | 3 | 2 |  |  |
| Mononchida | 5 | 5 | 5 | 5 | 5 | 5 | 5 | 5 | 5 | 5 |  |  |
| Plectida | 10 | 4 | 5 | 3 | 5 | 3 | 5 | 6 | 3 | 5 |  |  |
| Rhabditida | 37 | 26 | 21 | 16 | 25 | 36 | 24 | 25 | 18 | 25 |  |  |
| Strongylida | 4 | 4 | 4 | 4 | 4 | 4 | 4 | 4 | 4 | 4 |  |  |
| Trichinellida | 4 | 3 | 4 | 4 | 4 | 4 | 4 | 4 | 4 | 4 |  |  |
| Triplonchida | 8 | 4 | 8 | 6 | 4 | 8 | 8 | 8 | 8 | 8 |  |  |
| **B. Trees built by regional and combined sequences from reference species** | | | | | | | | | | |  |  |
| Order | Total no. | R1 | R2 | R3 | R4 | R1_2 | R3_4 | R2_3_4 | R1_2_3_4 | Reference (Full length) |  |  |
| Araeolaimida | 3 | 2 | 2 | 1 | 3 | 2 | 2 | 2 | 2 | 2 |  |  |
| Chromadorida | 4 | 3 | 3 | 2 | 1 | 3 | 2 | 2 | 2 | 2 |  |  |
| Desmodorida | 5 | 3 | 3 | 4 | 3 | 3 | 3 | 3 | 3 | 3 |  |  |
| Dorylaimida | 13 | 13 | 13 | 13 | 13 | 13 | 13 | 13 | 13 | 13 |  |  |
| Enoplida | 13 | 5 | 8 | 7 | 13 | 9 | 8 | 13 | 12 | 13 |  |  |
| Monhysterida | 5 | 3 | 3 | 4 | 2 | 3 | 2 | 5 | 5 | 2 |  |  |
| Mononchida | 5 | 5 | 5 | 4 | 5 | 5 | 5 | 5 | 5 | 5 |  |  |
| Plectida | 10 | 4 | 3 | 5 | 5 | 3 | 5 | 5 | 3 | 5 |  |  |
| Rhabditida | 37 | 36 | 23 | 17 | 25 | 36 | 24 | 25 | 18 | 25 |  |  |
| Strongylida | 4 | 4 | 4 | 4 | 4 | 4 | 4 | 4 | 4 | 4 |  |  |
| Trichinellida | 4 | 3 | 4 | 4 | 4 | 4 | 4 | 4 | 4 | 4 |  |  |
| Triplonchida | 8 | 5 | 8 | 4 | 8 | 8 | 4 | 8 | 8 | 8 |  |  |
