# Supplementary figures and images for: Taxonomic profiling of individual nematodes isolated from copse soils using deep amplicon sequencing of four distinct regions of the 18S ribosomal RNA gene
Source: PLoS One. 2020 Oct 7;15(10):e0240336. doi: 10.1371/journal.pone.0240336 (PMC7540906; doi:10.1371/journal.pone.0240336)

## Region 1

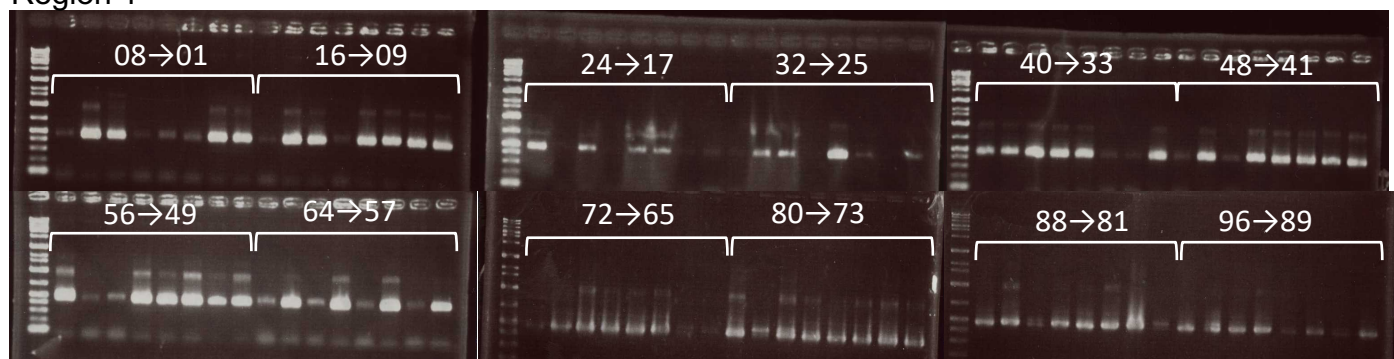

## Region 2

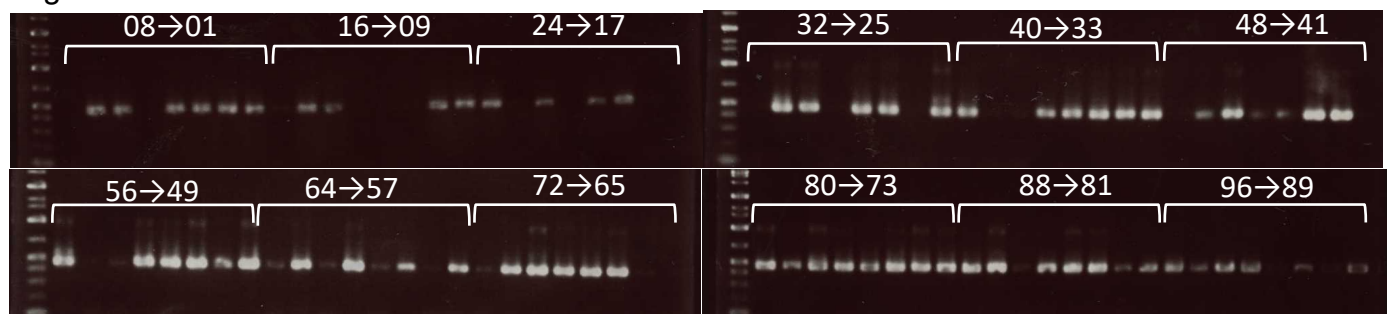

## Region 3

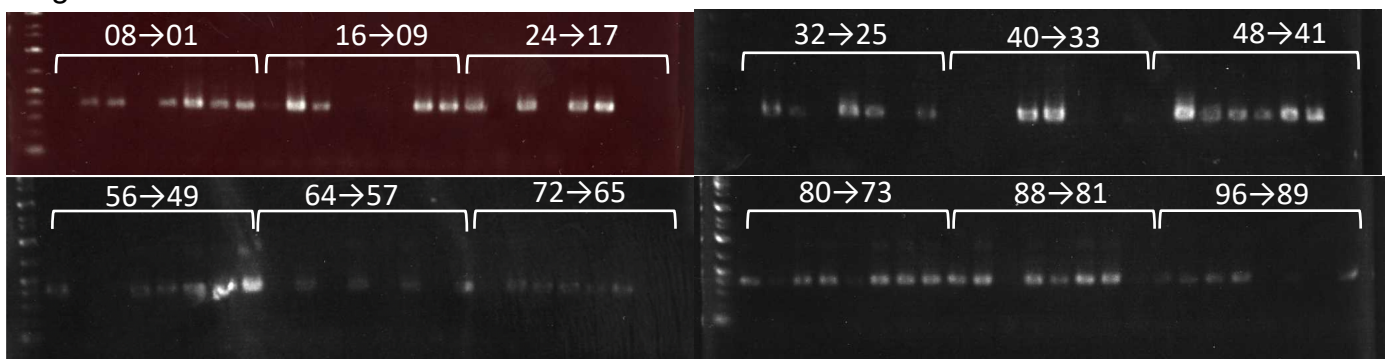

## Region 4

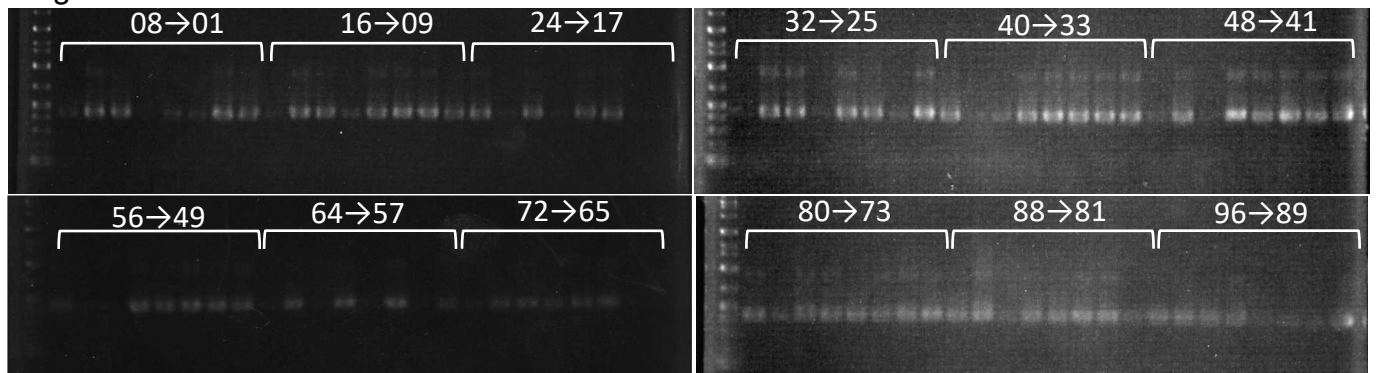

Supplement: S1 Fig — Five microliter aliquots of each reaction mixture containing nematode DNA with the indicated sample ID number were subjected to 1% agarose gel electrophoresis. The PCR products from the indicated target regions in the gels were visualized using successive ethidium bromide staining. Gene Ladder Wide 1 (Nippon Gene, Toyama, Japan) was used as a size marker. (PDF) [file pone.0240336.s006.pdf]

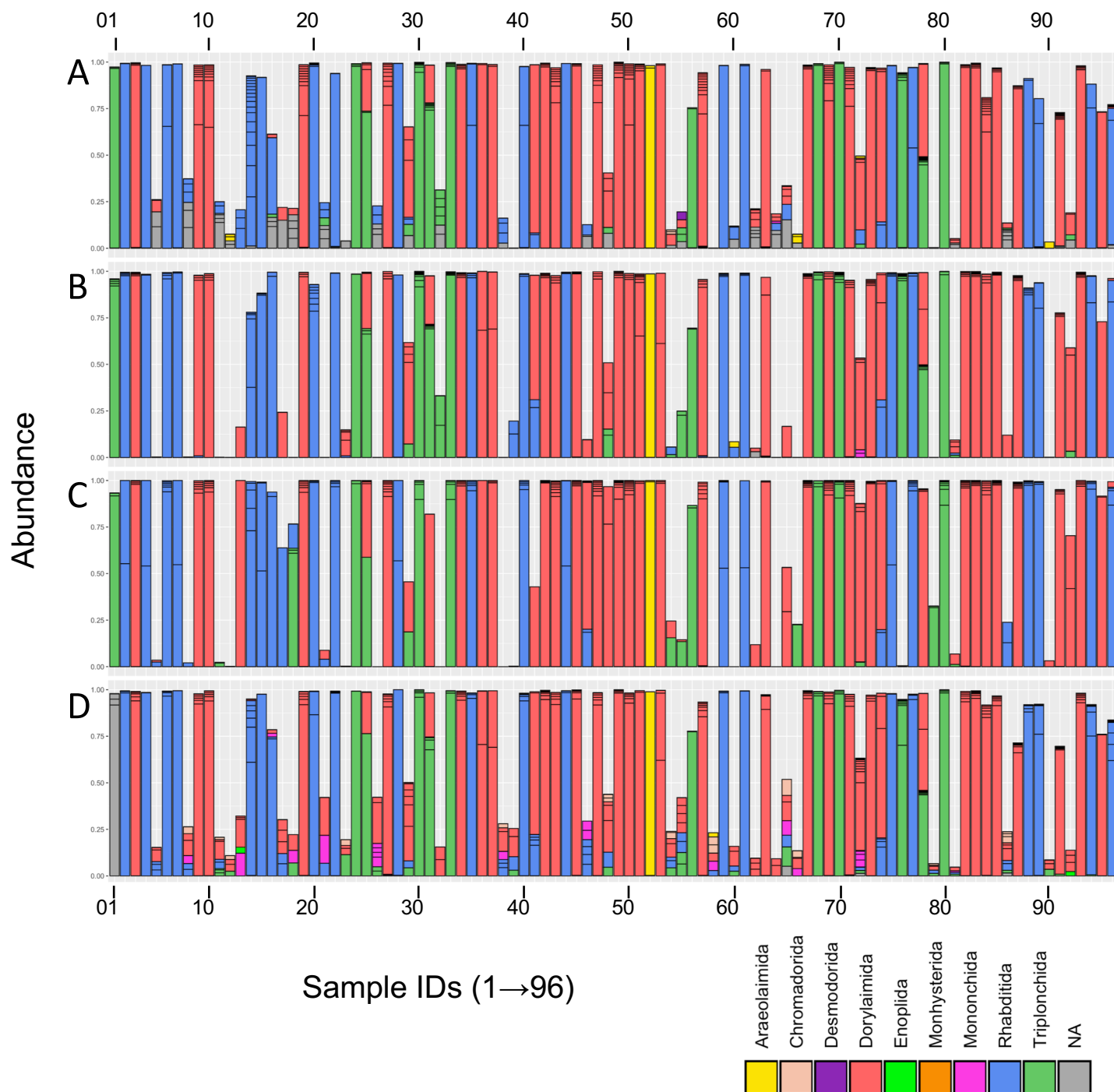

Supplement: S2 Fig — The abundance of nematode-derived SVs in each sample ID is shown in the histograms (A, region 1; B, region 2; C, region 3; and D, region 4). The orders of the SVs are also shown by the colors indicated above. Lines in the histogram indicate distinct SVs. NA: Not assigned. (PDF) [file pone.0240336.s007.pdf]

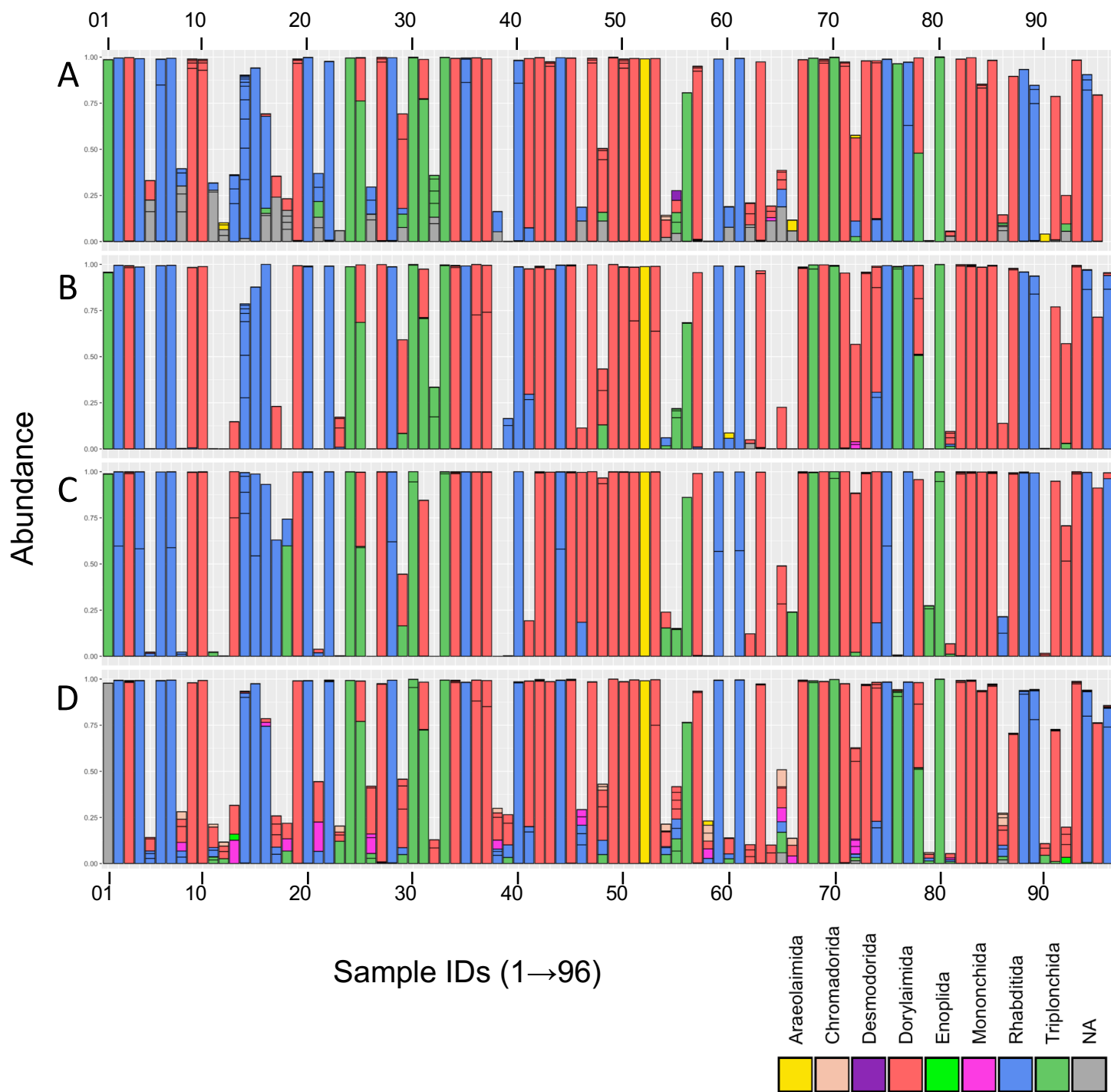

Supplement: S3 Fig — The abundance of nematode-derived SVs in each sample ID is shown in the histograms (A, region 1; B, region 2; C, region 3; and D, region 4). The orders of the SVs were also shown by the colors indicated above. Lines in the histogram indicate distinct SVs. NA: Not assigned. (PDF) [file pone.0240336.s008.pdf]

**R1\_2**

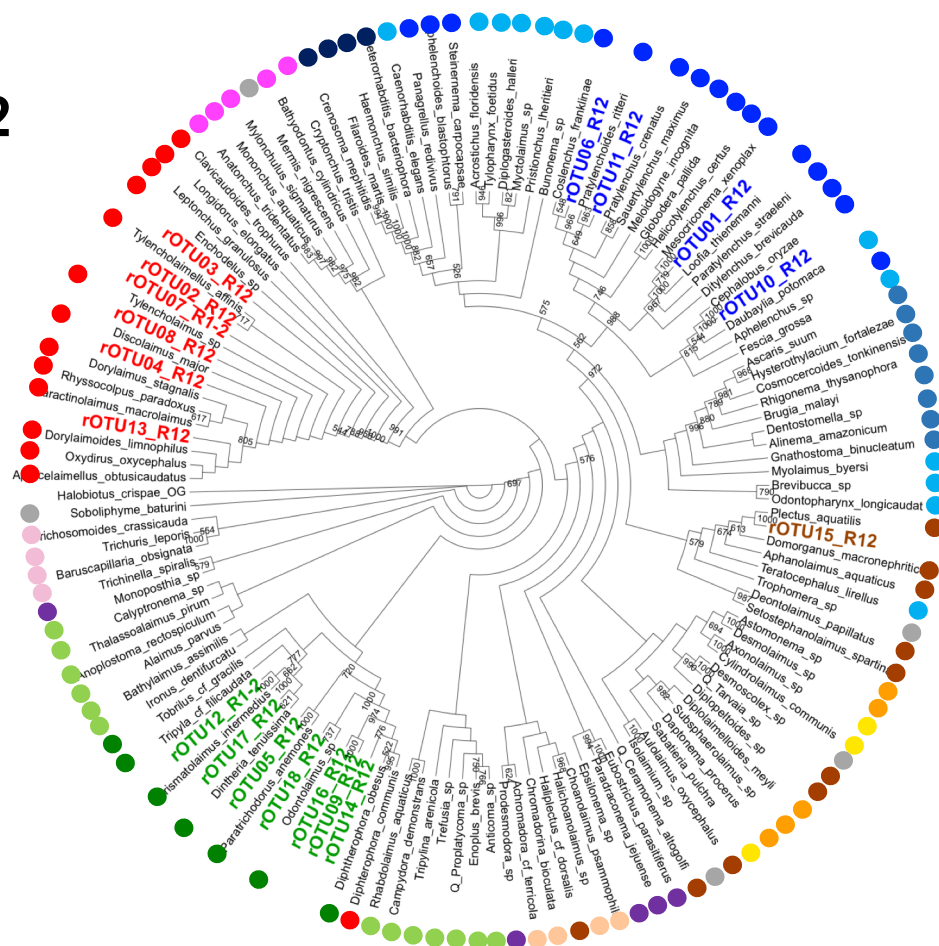

**R3\_4**

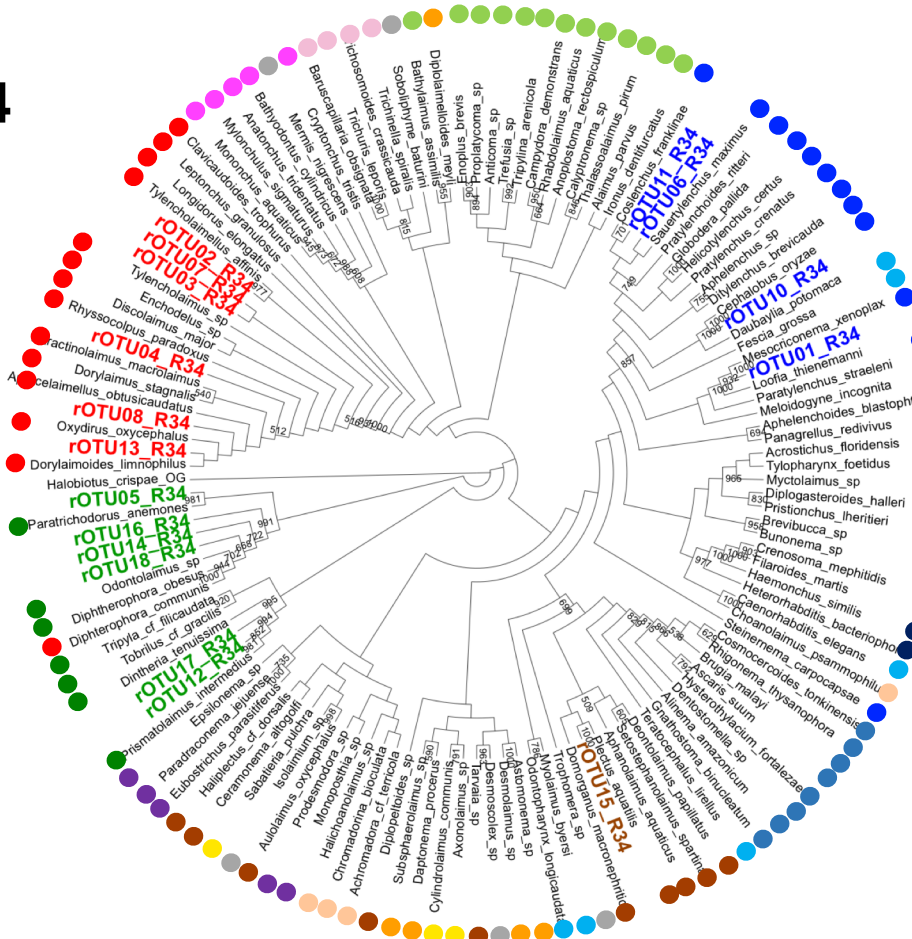

Supplement: S5 Fig — Two combined SSU regions are indicated in the upper left. R1_2 means the phylogenetic tree (cladogram) that was built using the combined nucleotide sequences of two SSU regions (regions 1 and 2) from nematode species, and the combined regional Z01rOTU_R1 and _R2 in this order. See the legend in S4 Fig for other descriptions on the phylogenetic tree. (PDF) [file pone.0240336.s010.pdf]

R2\_3\_4

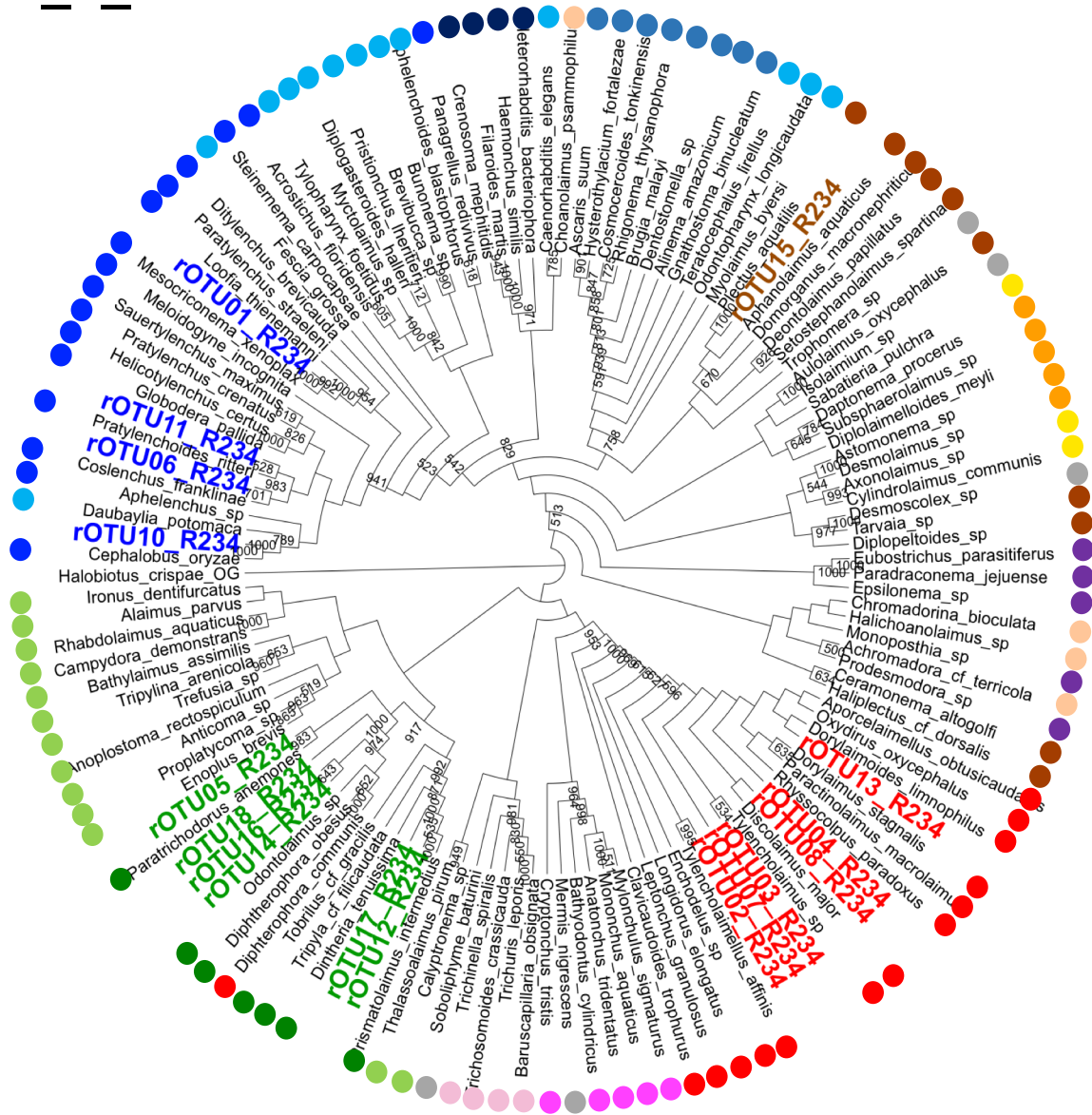

Supplement: S6 Fig — R2_3_4 means the phylogenetic tree (cladogram) was built using the combined nucleotide sequences of three SSU regions (regions 2, 3 and 4) from nematode species, and the combined regional Z01rOTU_R2, R3 and _R4 in this order. See the legend in S4 Fig for other descriptions on the phylogenetic tree. (PDF) [file pone.0240336.s011.pdf]

**Ref\_R1**

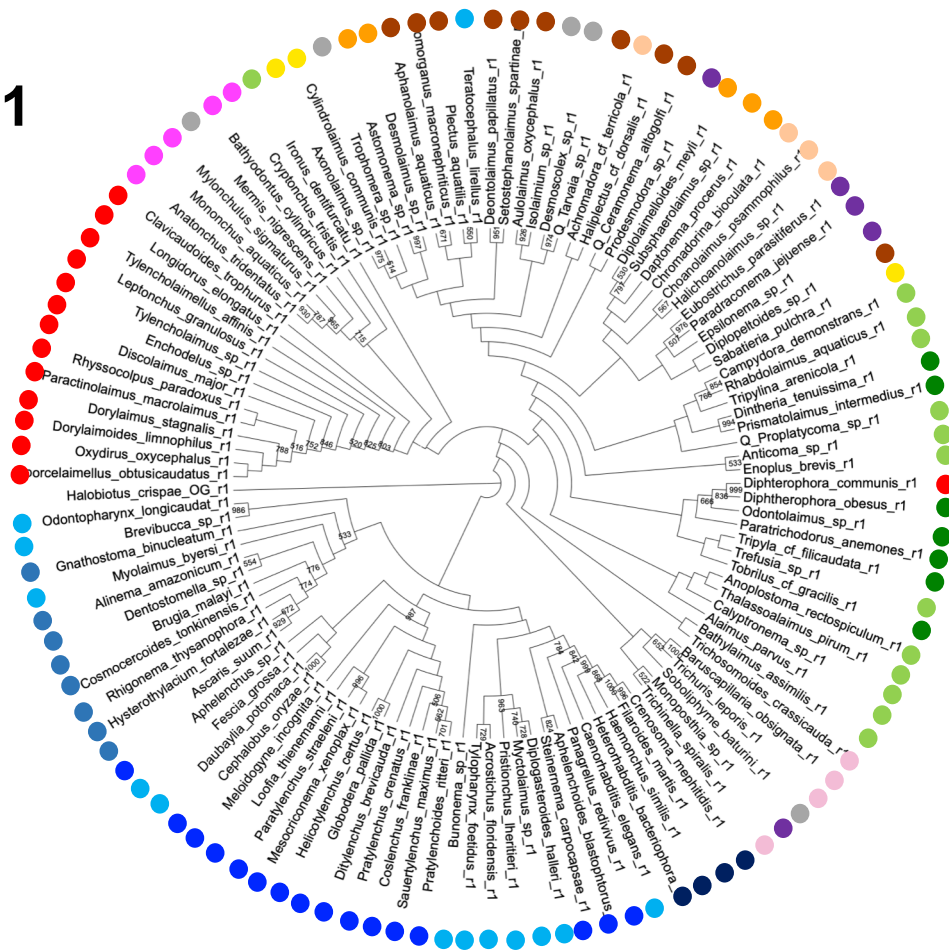

**Ref\_ R2**

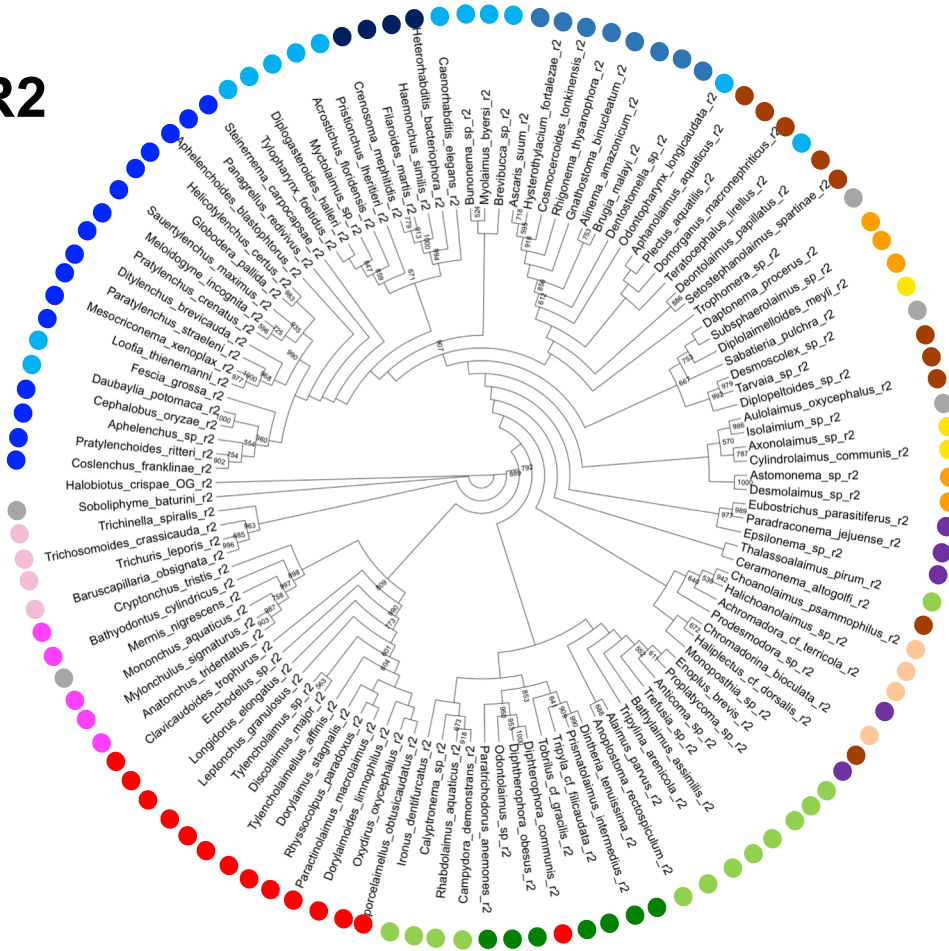

Supplement: S7 Fig — The SSU region of reference nematode species used for the preparation of the cladograms is indicated in the upper left. See the legend in S4 Fig for other descriptions on the phylogenetic tree. (PDF) [file pone.0240336.s012.pdf]

**Ref\_ R3**

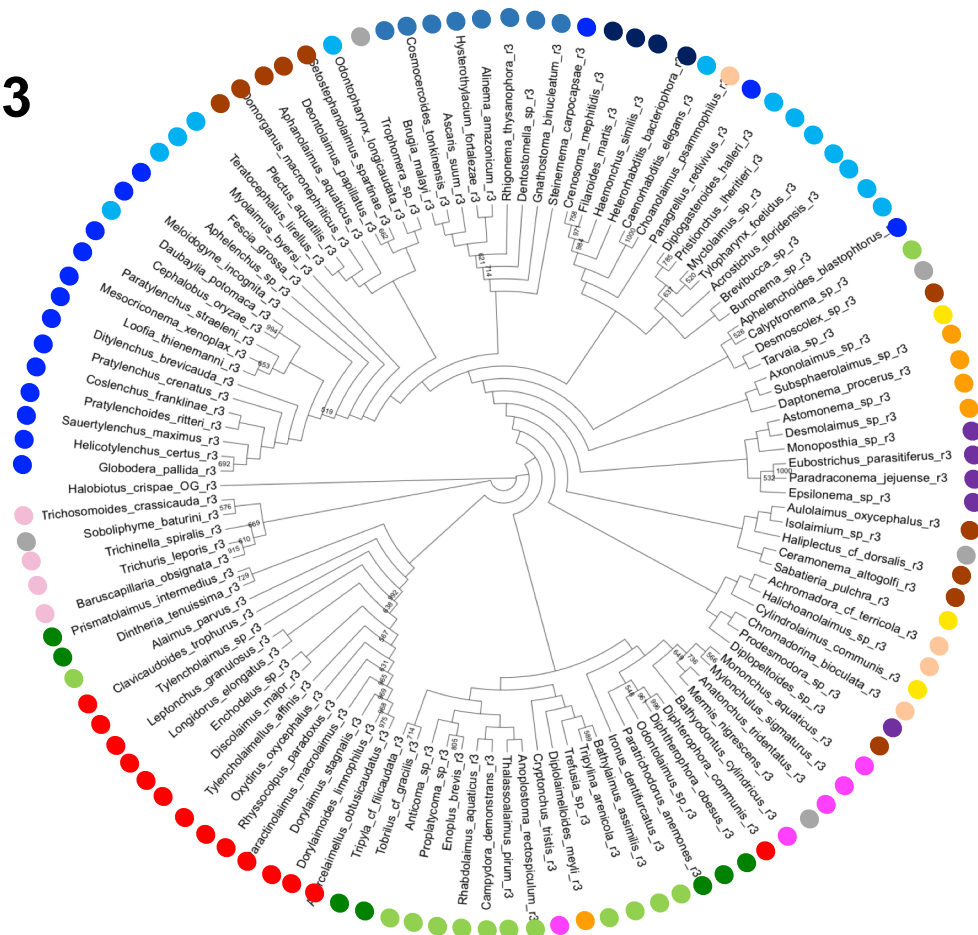

## Ref\_ R4

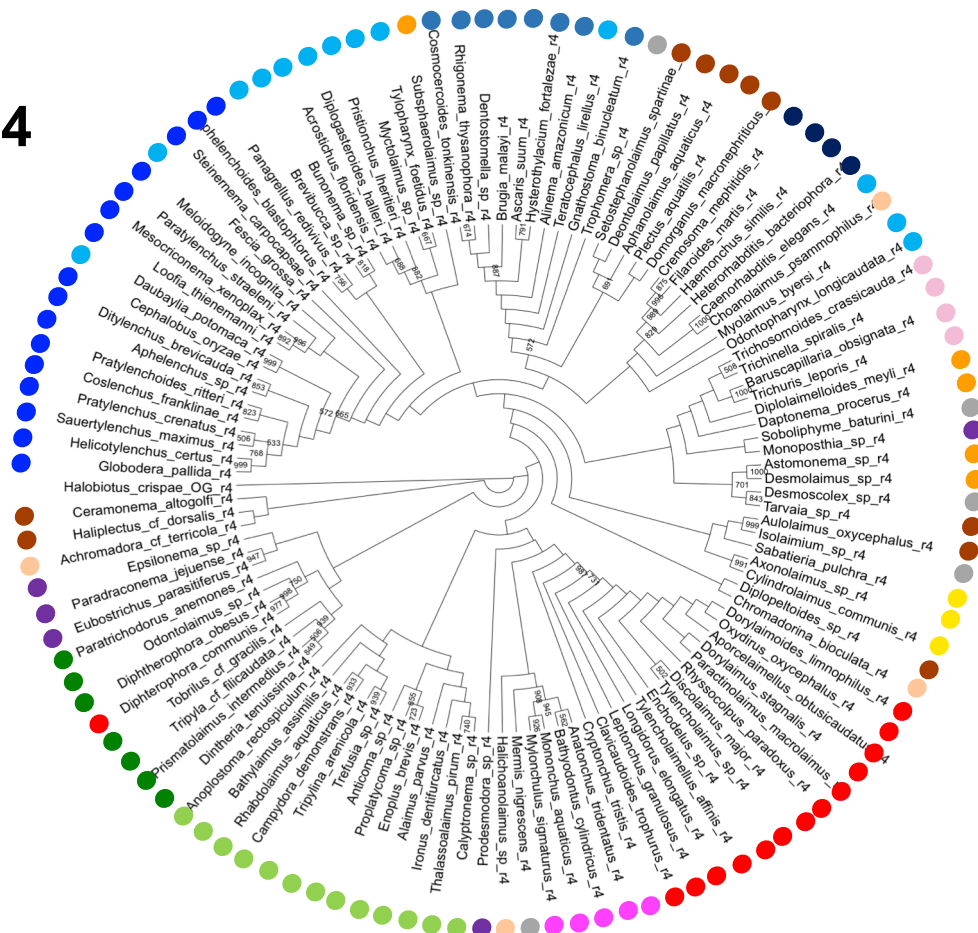

Supplement: S8 Fig — See the legend in S7 Fig for descriptions on the phylogenetic tree. (PDF) [file pone.0240336.s013.pdf]

**Ref\_ R3\_4**

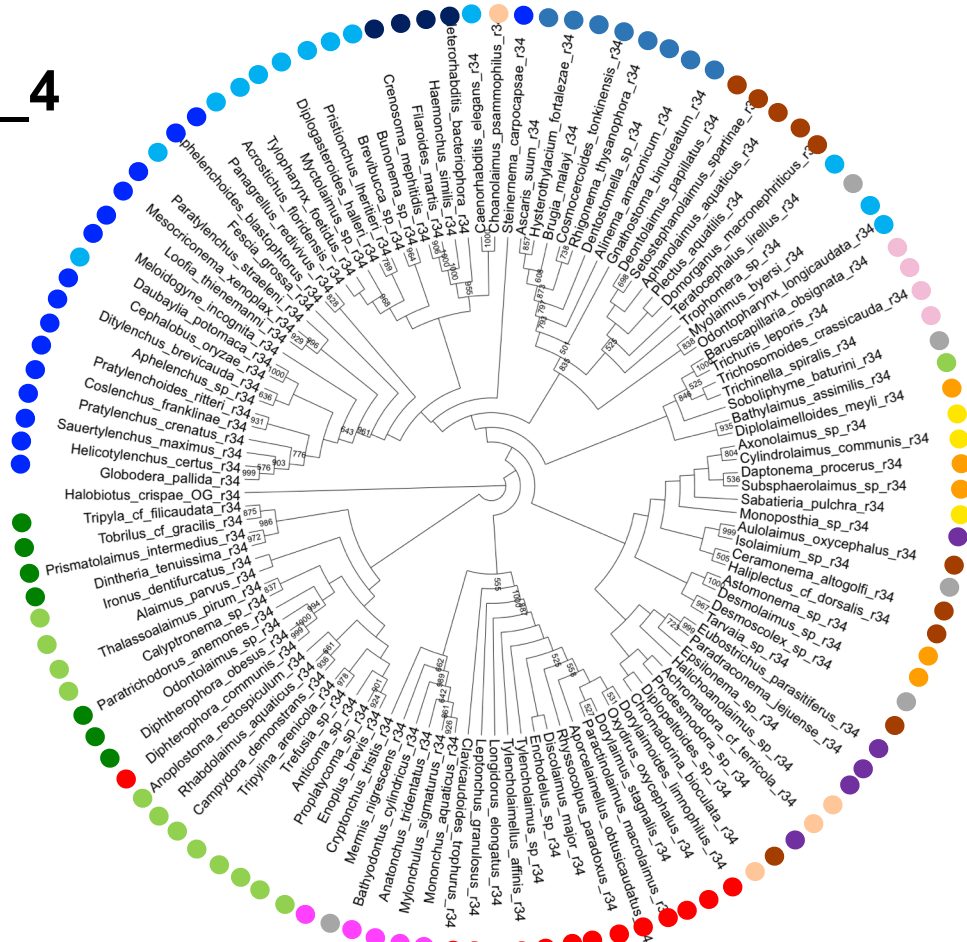

Supplement: S9 Fig — Two combined SSU regions used for the preparation of the cladograms are indicated in the upper left. Ref_R1_2 means the phylogenetic tree was built using the combined nucleotide sequences of the SSU regions 1 and 2 from nematode species in this order. See the legend in S4 Fig for other descriptions on the phylogenetic tree. (PDF) [file pone.0240336.s014.pdf]

**Ref\_ R2\_ 3\_ 4**

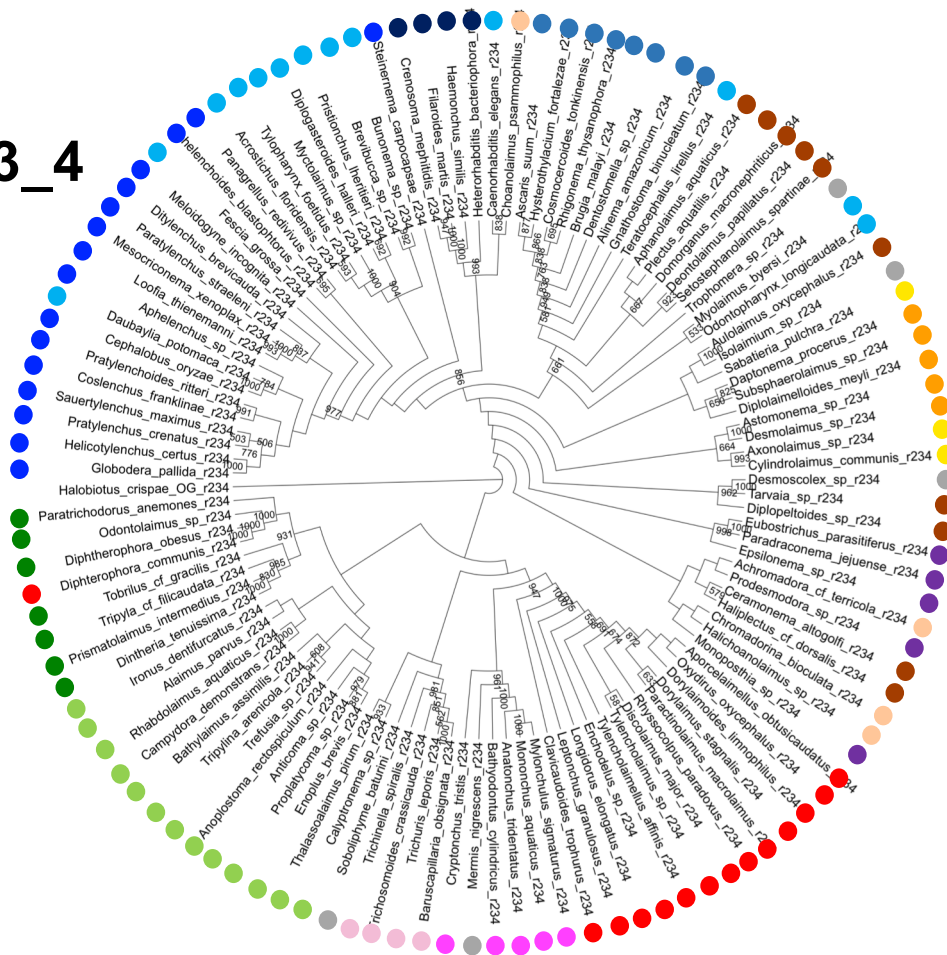

| Ref | R1 | 2 | 3 | 4 |
|-----|----|---|---|---|
|-----|----|---|---|---|

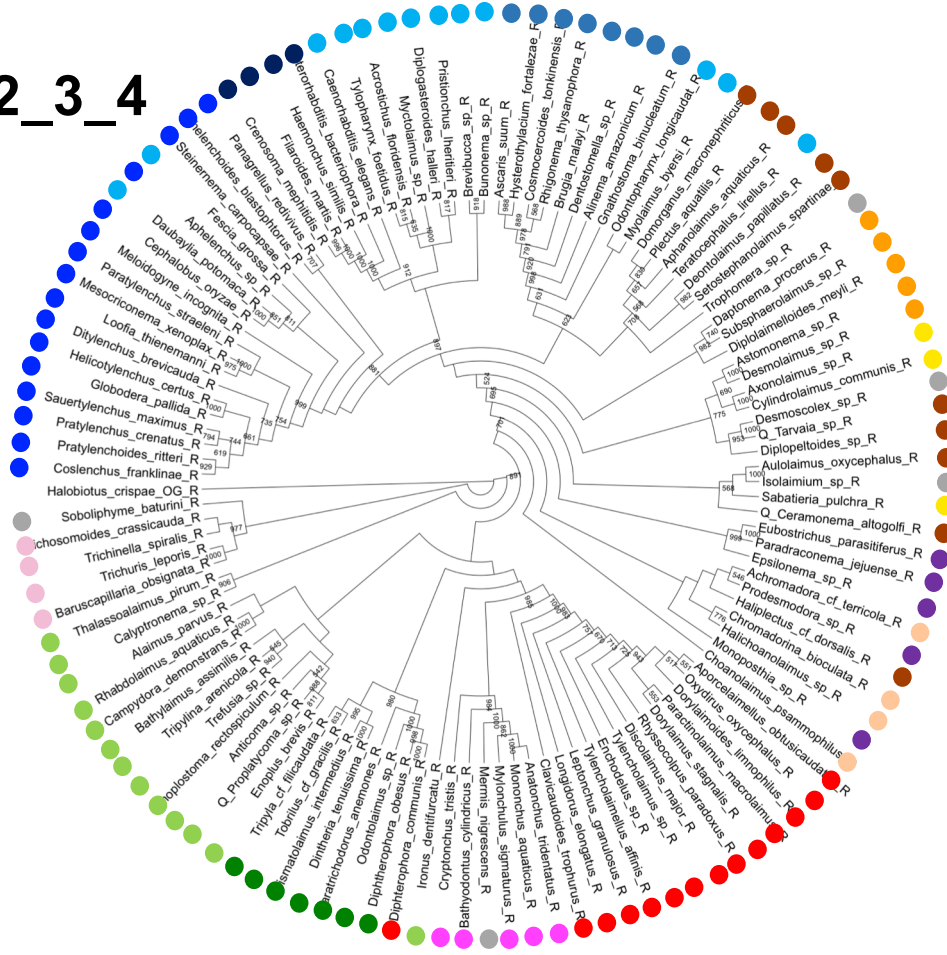

Supplement: S10 Fig — Combined SSU regions used for the preparation of the cladograms are indicated in the upper left. Ref_R2_3_4 means the phylogenetic tree built using the combined nucleotide sequences of the SSU regions 2, 3 and 4 from nematode species in this order. See the legend in S4 Fig for other descriptions on the phylogenetic tree. (PDF) [file pone.0240336.s015.pdf]
