# Supplementary material for: Taxonomic profiling of individual nematodes isolated from copse soils using deep amplicon sequencing of four distinct regions of the 18S ribosomal RNA gene
Source: PLoS One. 2020 Oct 7;15(10):e0240336. doi: 10.1371/journal.pone.0240336 (PMC7540906; doi:10.1371/journal.pone.0240336)
Supplement: S4 Fig — Nucleotide sequences of the SSU gene from 116 reference nematodes and Halobiotus crispae (phylum Tardigrada) were aligned, and the phylogenetic tree was prepared using the BOOTSTRAP N-J TREE algorithm (bootstrap: 1000 replicates) with the ClustalX package. In the resultant cladogram, a colored circle corresponding to the scientific name indicates the species’ order, as shown in the box below. The orders were classified into two classes of phylum Nematoda (Chromadorea and Enoplea), and two subclasses of class Enoplea (Enoplia and Dorylaimia). The species that belong to the order Rhabditida were further classified into three groups (i.e., two suborders Tylenchina and Spirurina, and others) in parentheses. The order Benthimermithida belongs to the phylum Nematoda with no rank. Bootstrap numbers of over 500 per 1000 were indicated at the nodes of the cladogram. The abbreviation “Ref_FL” means the phylogenetic tree built by full-length sequences of nematode reference species. (PDF) [file pone.0240336.s009.pdf]

Ref_FL

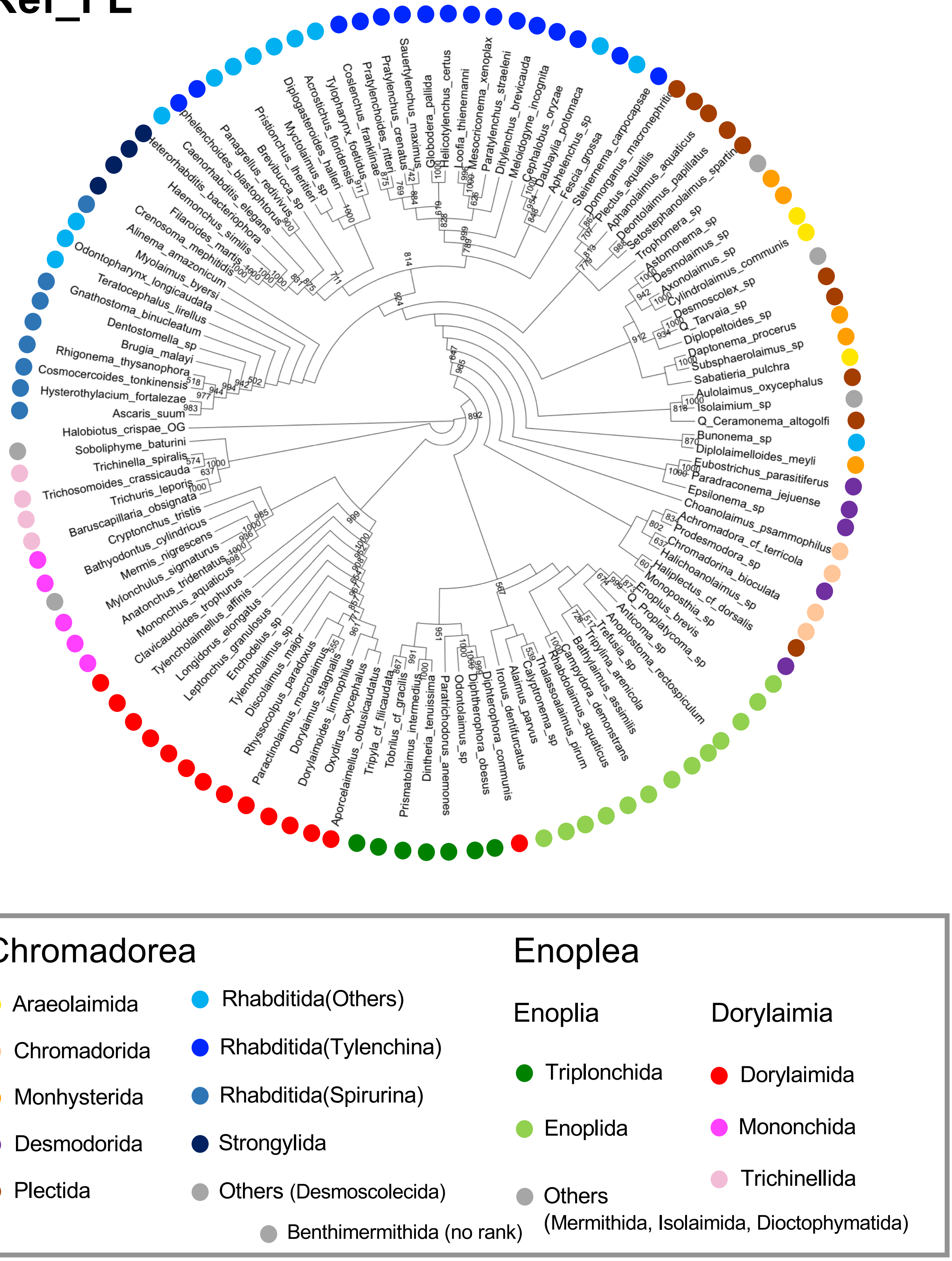

Chromadorea
Araeolaimida
Chromadorida
Monhysterida
Desmodorida
Plectida
Rhabditida(Others)
Rhabditida(Tylenchina)
Rhabditida(Spirurina)
Strongylida
Others (Desmoscolecida)
Benthimermithida (no rank)
Enoplea
Enoplia
Triplonchida
Enoplida
Others
(Mermithida, Isolaimida, Dioctophymatida)
Dorylaimia
Dorylaimida
Mononchida
Trichinellida
