# Supplementary material for: Taxonomic profiling of individual nematodes isolated from copse soils using deep amplicon sequencing of four distinct regions of the 18S ribosomal RNA gene
Source: PLoS One. 2020 Oct 7;15(10):e0240336. doi: 10.1371/journal.pone.0240336 (PMC7540906; doi:10.1371/journal.pone.0240336)
Supplement: S1 Raw images — The PCR products from the indicated target regions in the gels were visualized using successive ethidium bromide staining. Fluorescent images of agarose gels were acquired using the FAS-III gel imaging system (Nippon Genetics Co., Tokyo, Japan), and original TIFF images shown in each file were used to prepare four regional combined figures of S1 Fig after removing unrelated area. The sample ID in each lane was indicated on top of a gel image and correspond to the sample ID numbers in parentheses in S1 Fig. The amplified region and sample IDs contained in each gel were also indicated at the upper left in each file. M: lane with a size marker (Gene Ladder Wide 1). (PDF) [file pone.0240336.s016.pdf]

S1_raw_images

Below are the raw gel images for S1 Fig. The only modifications that occurred was cropping of images to exclude unrelated area.

Region 1 (no.01-16)

M 08 07 06 05 04 03 02 01 16 15 14 13 12 11 10 09

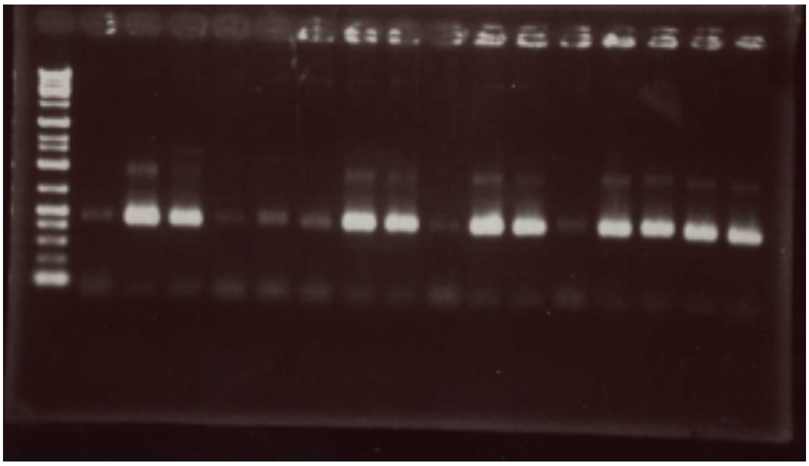

The above is the uncropped original image for the Region 1 panel (sample ID number 01-16) of S1 Fig. The image was cropped to all lanes including a size marker (M)(Gene Ladder Wide 1, Nippon Gene, Toyama, Japan).
Loading order: From left to right. Sample IDs in the gel correspond to the numbers (08-01, 16-09) in parentheses in S1 Fig.

In the PCR experiments, aliquots of each reaction mixture containing nematode DNA with the indicated sample ID number were subjected to 1% agarose gel electrophoresis (eight samples per group). The PCR products from the target regions in the gels were visualized using successive ethidium bromide staining. Fluorescent images of agarose gels were acquired using the FAS-III gel imaging system (Nippon Genetics Co., Tokyo, Japan) as a TIFF file. The sample ID in each lane was indicated on top of a gel image.

Region 1 (no.17-32)

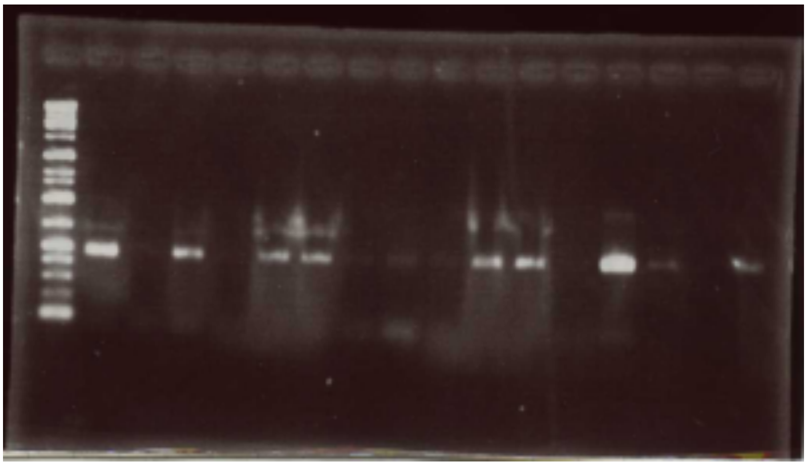

The above is the uncropped original image for the Region 1 panel (sample ID number 17-32) of S1 Fig. The all lanes were used after removing unrelated area. The sample IDs used for PCR were indicated on top of lanes. M: a size marker.

Loading order: From left to right. Sample IDs in the gel correspond to the numbers (24-17, 32-25) in parentheses in S1 Fig.

Region 1 (no.33-48)

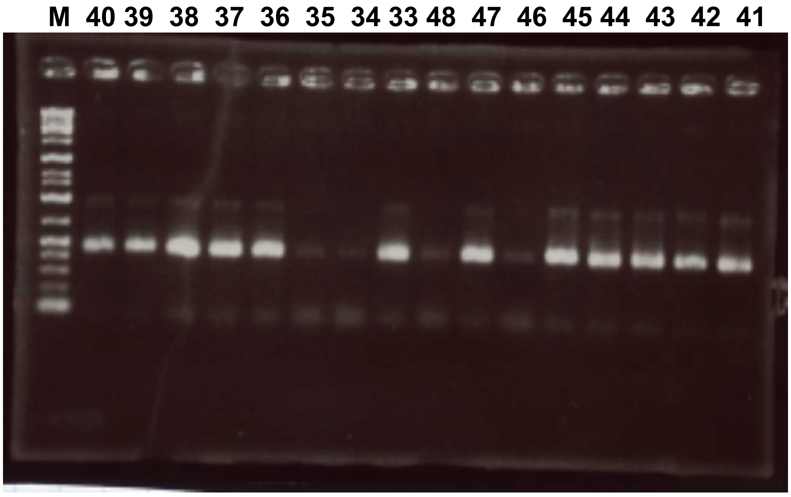

The above is the uncropped original image for the Region 1 panel (sample ID number 33-48) of S1 Fig. The all lanes were used after removing unrelated area. The sample IDs used for PCR were indicated on top of lanes. M: a size marker.
Loading order: From left to right. Sample IDs in the gel correspond to the numbers (40-33, 48-41) in parentheses in S1 Fig.

Region 1 (no.49-64)

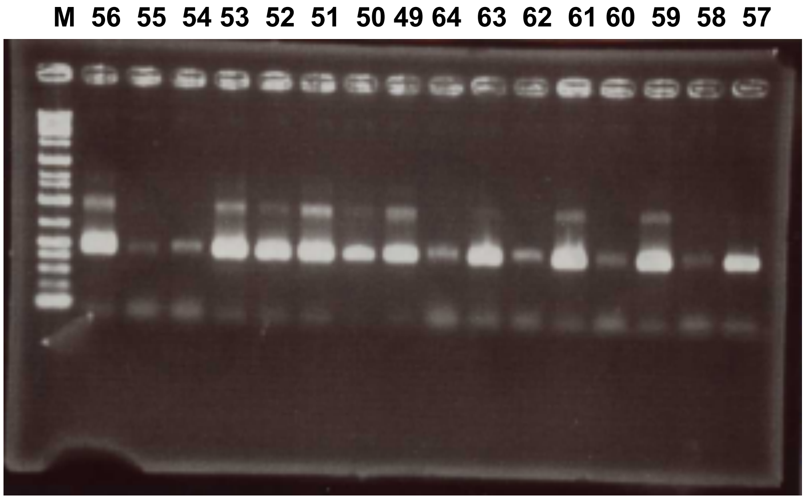

The above is the uncropped original image for the Region 1 panel (sample ID number 49-64) of S1 Fig. The all lanes were used after removing unrelated area. The sample IDs used for PCR were indicated on top of lanes. M: a size marker.
Loading order: From left to right. Sample IDs in the gel correspond to the numbers (56-49, 64-57) in parentheses in S1 Fig.

Region 1 (no.65-80)

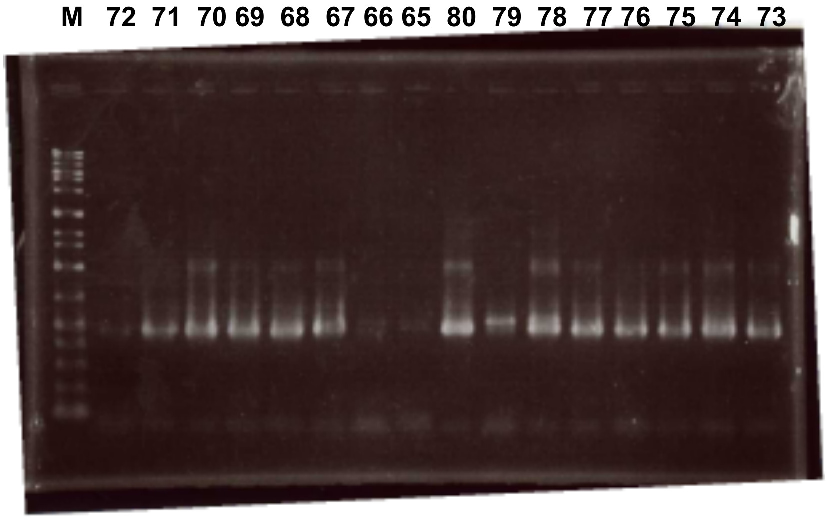

The above is the uncropped original image for the Region 1 panel (sample ID number 65-80) of S1 Fig. The all lanes were used after removing unrelated area. The sample IDs used for PCR were indicated on top of lanes. M: a size marker.
Loading order: From left to right. Sample IDs in the gel correspond to the numbers (72-65, 80-73) in parentheses in S1 Fig.

Region 1 (no.81-96)

M 88 87 86 85 84 83 82 81 96 95 94 93 92 91 90 89

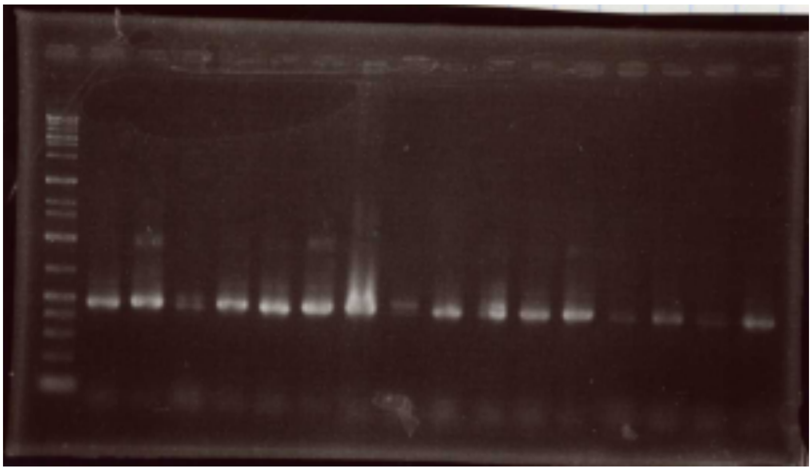

The above is the uncropped original image for the Region 1 panel (sample ID number 81-96) of S1 Fig. The all lanes were used after removing unrelated area. The sample IDs used for PCR were indicated on top of lanes. M: a size marker.
Loading order: From left to right. Sample IDs in the gel correspond to the numbers (88-81, 96-89) in parentheses in S1 Fig.

Region 2 (no.01-24)

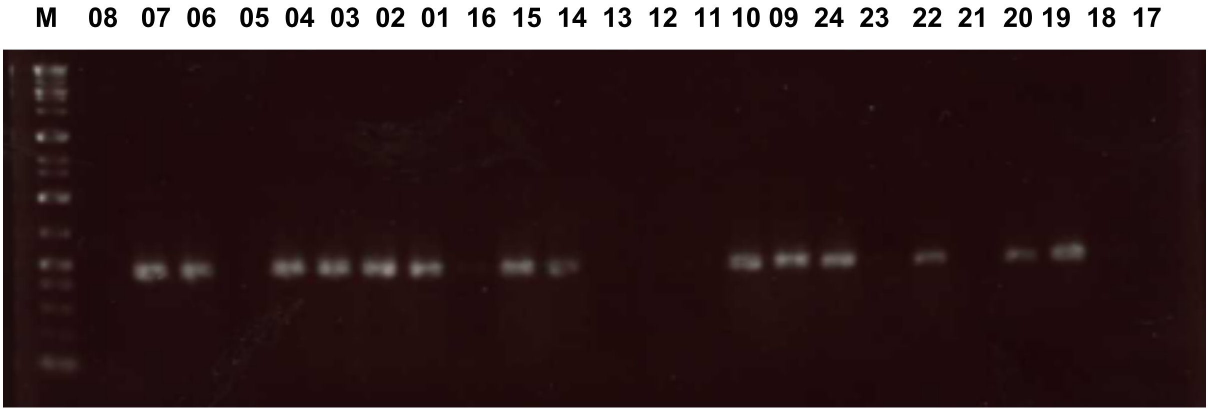

The above is the uncropped original image for the Region 2 panel (sample ID number 01-24) of S1 Fig. The all lanes were used after removing unrelated area. The sample IDs used for PCR were indicated on top of lanes. M: a size marker.

Loading order: From left to right. Sample IDs in the gel correspond to the numbers (08-01, 16-09, and 24-17) in parentheses in S1 Fig.

Region 2 (no.25-48)

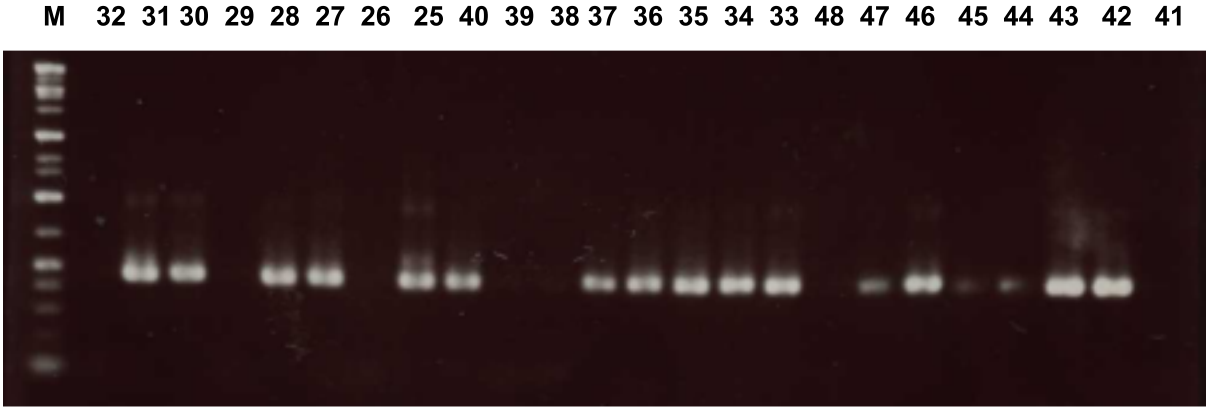

The above is the uncropped original image for the Region 2 panel (sample ID number 25-48) of S1 Fig. The all lanes were used after removing unrelated area. The sample IDs used for PCR were indicated on top of lanes. M: a size marker.
Loading order: From left to right. Sample IDs in the gel correspond to the numbers (32-25, 40-33, and 48-41) in parentheses in S1 Fig.

Region 2 (no.49-72)

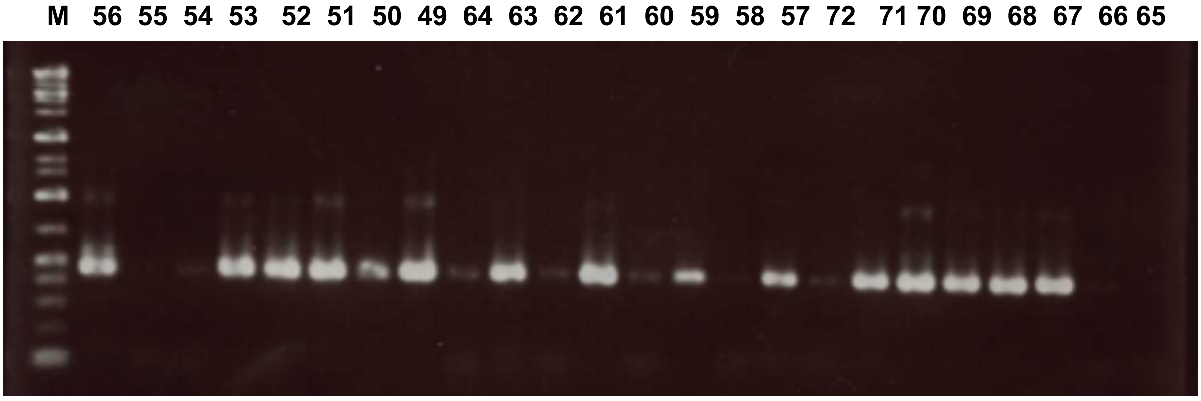

The above is the uncropped original image for the Region 2 panel (sample ID number 49-72) of S1 Fig. The all lanes were used after removing unrelated area. The sample IDs used for PCR were indicated on top of lanes. M: a size marker.
Loading order: From left to right. Sample IDs in the gel correspond to the numbers (56-49, 64-57, and 72-65) in parentheses in S1 Fig.

Region 2 (no.73-96)

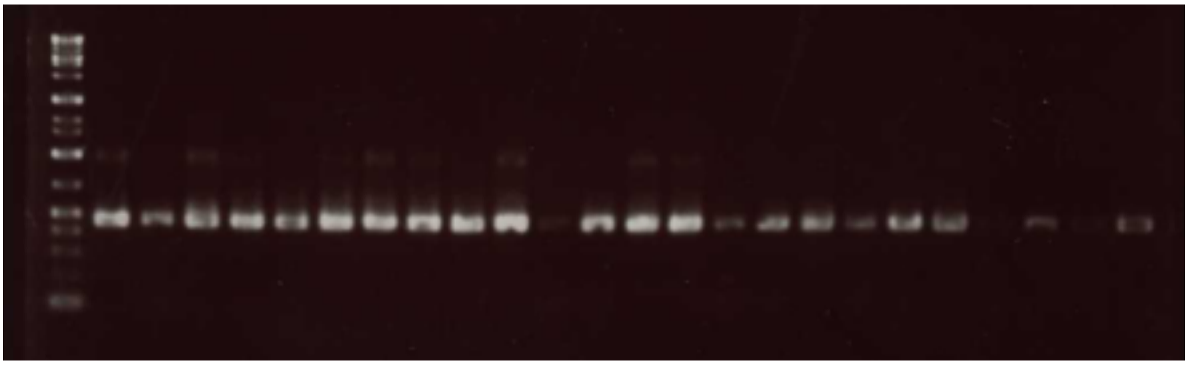

The above is the uncropped original image for the Region 2 panel (sample ID number 73-96) of S1 Fig. The all lanes were used after removing unrelated area. The sample IDs used for PCR were indicated on top of lanes. M: a size marker.
Loading order: From left to right. Sample IDs in the gel correspond to the numbers (80-73, 88-81, and 96-89) in parentheses in S1 Fig.

Region 3 (no.01-24)

M 08 07 06 05 04 03 02 01 16 1514 13 12 11 10 09 24 23 22 21 20 19 18 17

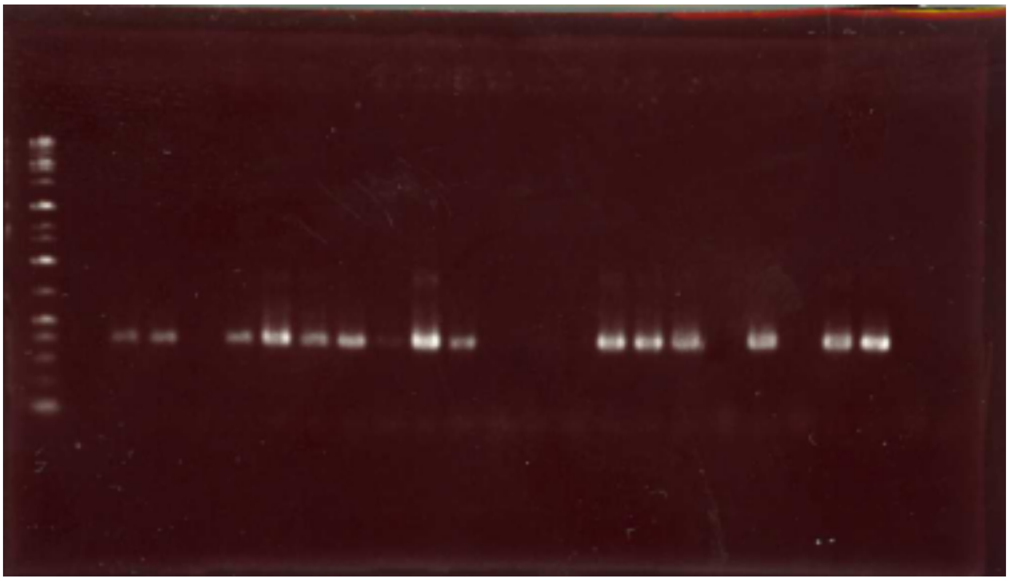

The above is the uncropped original image for the Region 3 panel (sample ID number 01-24) of S1 Fig. The all lanes were used after removing unrelated area. The sample IDs used for PCR were indicated on top of lanes. M: a size marker.
Loading order: From left to right. Sample IDs in the gel correspond to the numbers (08-01, 16-09, and 24-17) in parentheses in S1 Fig.

Region 3 (no.25-48)

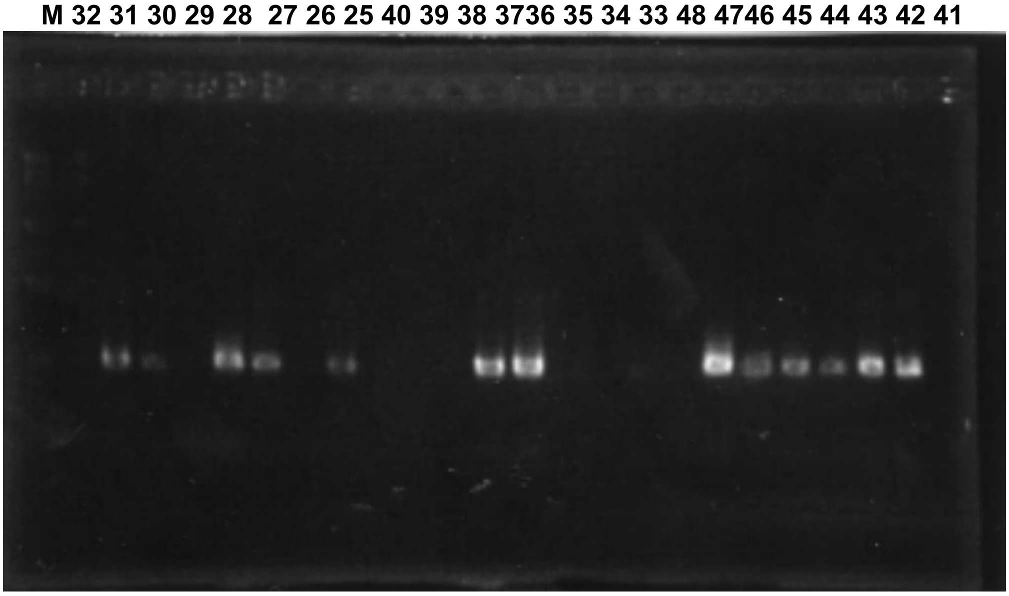

The above is the uncropped original image for the Region 3 panel (sample ID number 25-48) of S1 Fig. The all lanes were used after removing unrelated area. The sample IDs used for PCR were indicated on top of lanes. M: a size marker.
Loading order: From left to right. Sample IDs in the gel correspond to the numbers (32-25, 40-33, and 48-41) in parentheses in S1 Fig.
The marker is hardly visible due to leak from a lane, but visible in the lighter gel image in the last figure.

Region 3 (no.49-72)

M 56 55 54 53 52 51 50 49 64 63 62 61 60 59 58 57 72 71 70 69 68 67 66 65

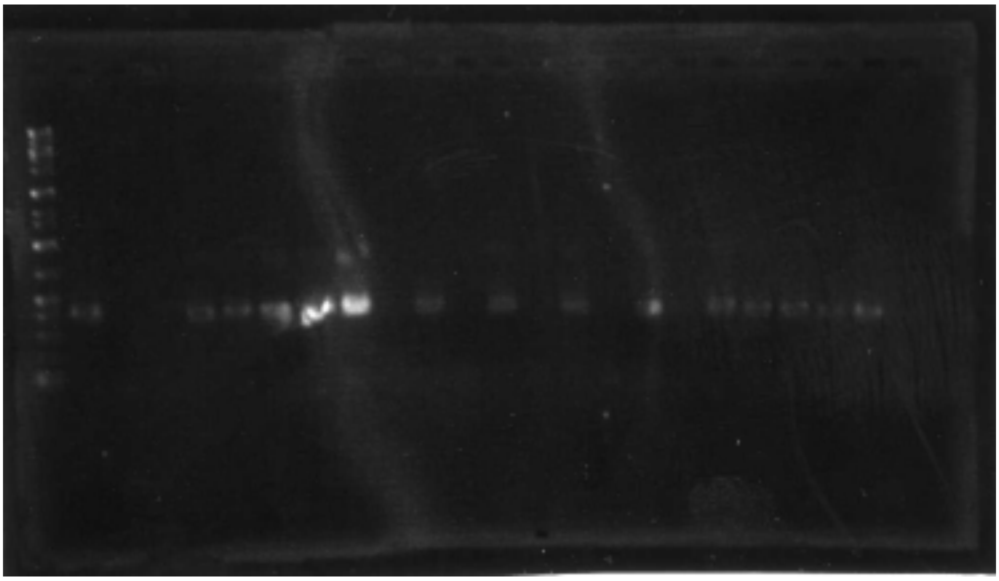

The above is the uncropped original image for the Region 3 panel (sample ID number 49-72) of S1 Fig. The all lanes were used after removing unrelated area. The sample IDs used for PCR were indicated on top of lanes. M: a size marker.
Loading order: From left to right. Sample IDs in the gel correspond to the numbers (56-49, 64-57, and 72-65) in parentheses in S1 Fig.

Region 3 (no.73-96)

M 80 79 78 77 76 75 74 73 88 8786 85 84 83 82 8196 95 9493 92 91 90 89

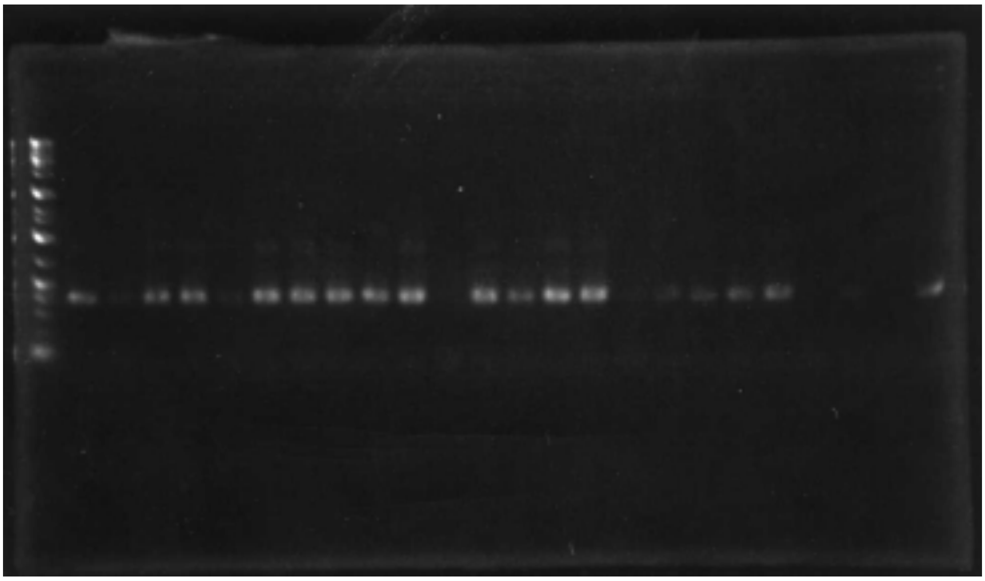

The above is the uncropped original image for the Region 3 panel (sample ID number 73-96) of S1 Fig. The all lanes were used after removing unrelated area. The sample IDs used for PCR were indicated on top of lanes. M: a size marker.
Loading order: From left to right. Sample IDs in the gel correspond to the numbers (80-73, 88-81, and 96-89) in parentheses in S1 Fig.

Region 4 (no.01-24)

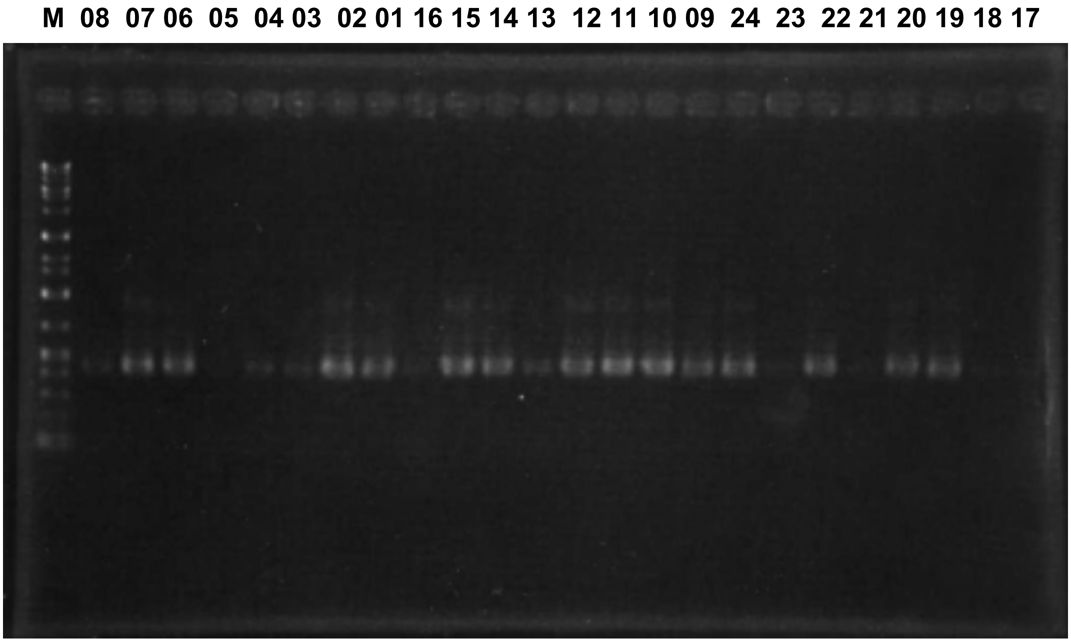

The above is the uncropped original image for the Region 4 panel (sample ID number 01-24) of S1 Fig. The all lanes were used after removing unrelated area. The sample IDs used for PCR were indicated on top of lanes. M: a size marker.
Loading order: From left to right. Sample IDs in the gel correspond to the numbers (08-01, 16-09, and 24-17) in parentheses in S1 Fig.

Region 4 (no.25-48)

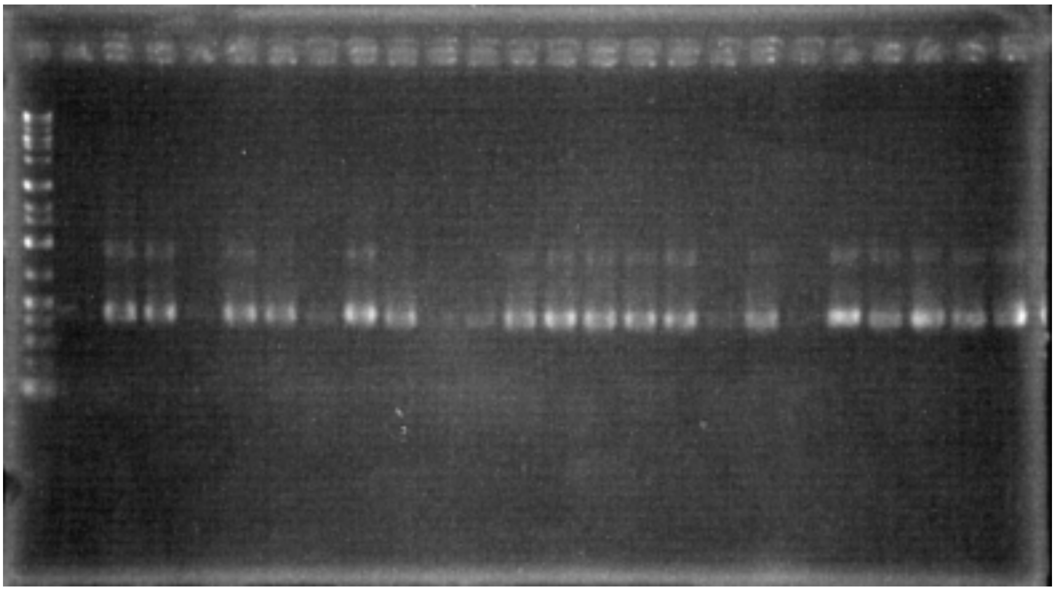

The above is the uncropped original image for the Region 4 panel (sample ID number 25-48) of S1 Fig. The all lanes were used after removing unrelated area. The sample IDs used for PCR were indicated on top of lanes. M: a size marker.
Loading order: From left to right. Sample IDs in the gel correspond to the numbers (32-25, 40-33, and 48-41) in parentheses in S1 Fig.

Region 4 (no.49-72)

M 56 55 54 53 52 51 50 49 64 63 62 61 60 59 58 57 72 71 70 69 68 67 66 65

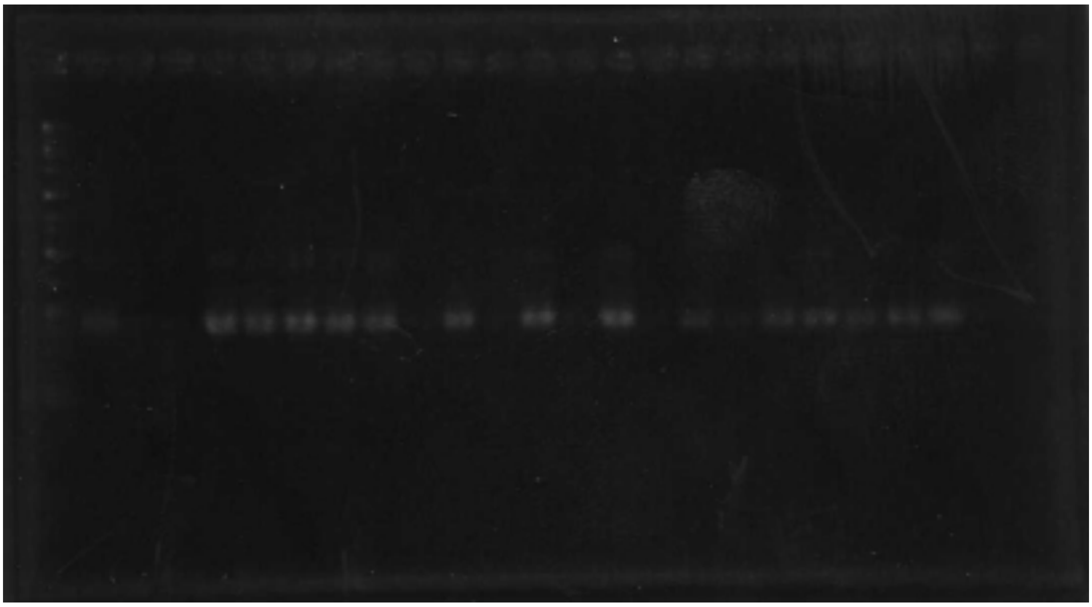

The above is the uncropped original image for the Region 4 panel (sample ID number 49-72) of S1 Fig. The all lanes were used after removing unrelated area. The sample IDs used for PCR were indicated on top of lanes. M: a size marker.
Loading order: From left to right. Sample IDs in the gel correspond to the numbers (56-49, 64-57, and 72-65) in parentheses in S1 Fig.
The marker is hardly visible, but visible in the lighter gel image in the last figure.

Region 4 (no.73-96)

M 80 79 78 77 76 75 74 73 88 87 86 85 84 83 82 81 96 95 94 93 92 91 90 89

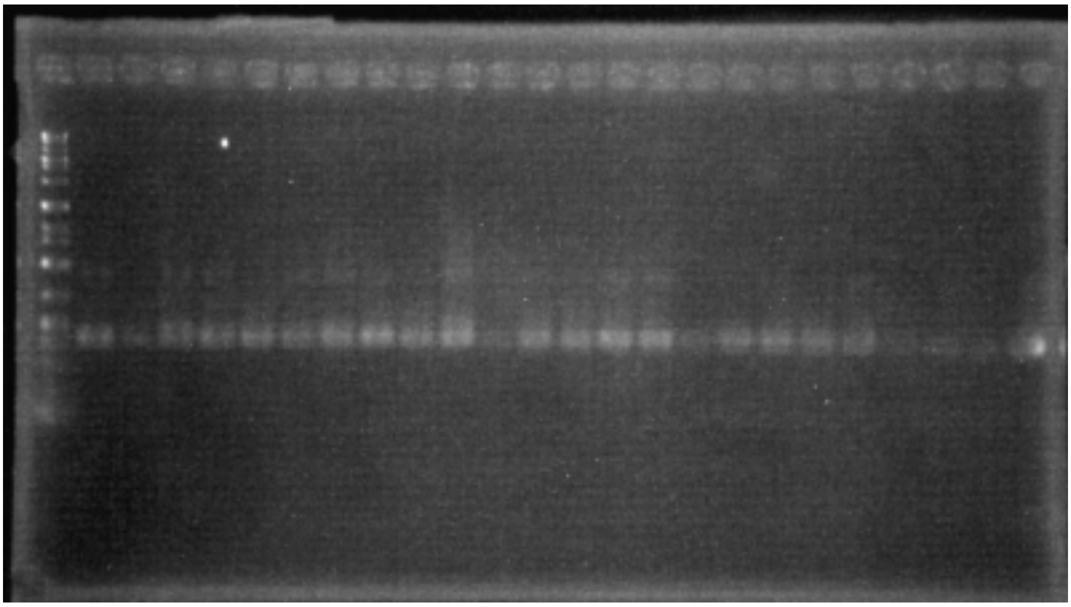

The above is the uncropped original image for the Region 4 panel (sample ID number 73-96) of S1 Fig. The all lanes were used after removing unrelated area. The sample IDs used for PCR were indicated on top of lanes. M: a size marker.

Loading order: From left to right. Sample IDs in the gel correspond to the numbers (80-73, 88-81, and 96-89) in parentheses in S1 Fig.

Region 3 (no.25-48)

M 32 31 30 29 28 27 26 25 40 39 38 3736 35 34 33 48 4746 45 44 43 42 41

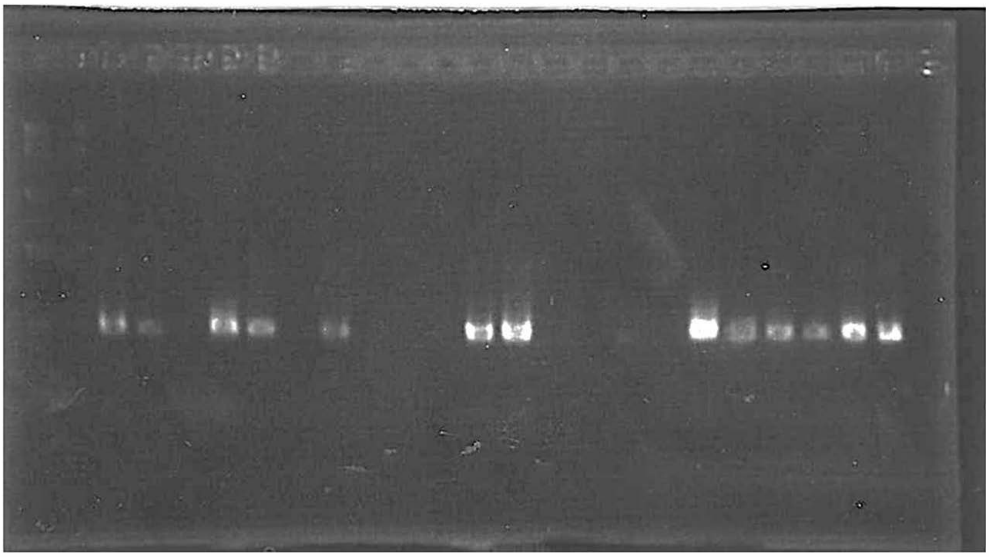

Region 4 (no.49-72)

M 56 55 54 53 52 51 50 49 64 63 62 61 60 59 58 57 72 71 70 69 68 67 66 65

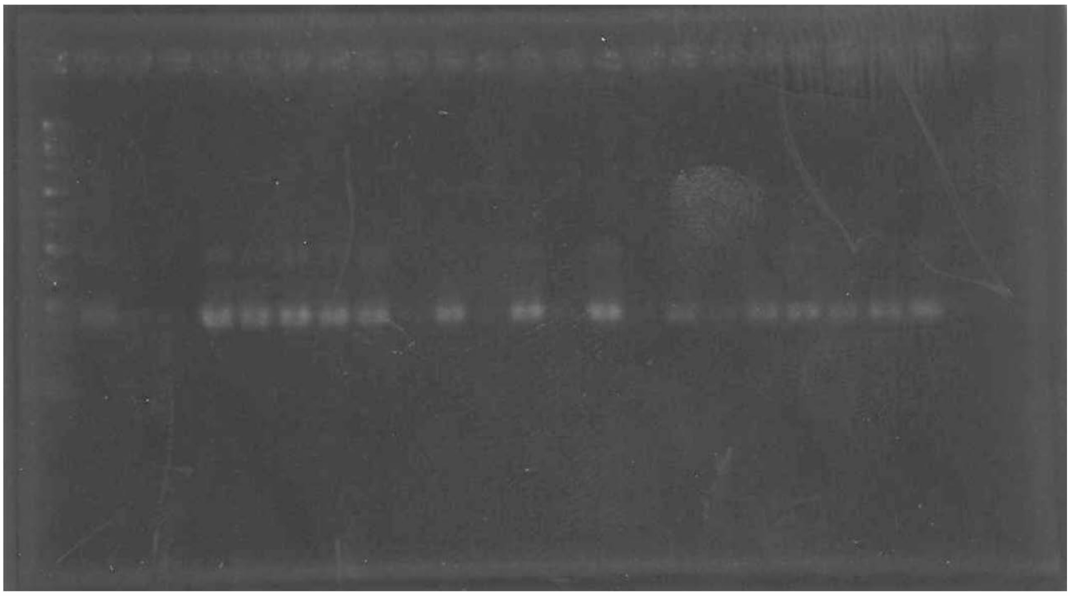

These lighter images were prepared from the original images to show size markers in the gels.
